# Gene replacement therapy in Bietti crystalline corneoretinal dystrophy: an open-label, single-arm, exploratory trial

**DOI:** 10.1038/s41392-024-01806-3

**Published:** 2024-04-24

**Authors:** Jinyuan Wang, Jinlu Zhang, Shicheng Yu, Hongyan Li, Shaohong Chen, Jingting Luo, Haibo Wang, Yuxia Guan, Haihan Zhang, Shiyi Yin, Huili Wang, Heping Li, Junle Liu, Jingyuan Zhu, Qiong Yang, Ying Sha, Chuan Zhang, Yuhang Yang, Xuan Yang, Xifang Zhang, Xiuli Zhao, Likun Wang, Liping Yang, Wenbin Wei

**Affiliations:** 1 Beijing Tongren Eye Center, Beijing Key Laboratory of Intraocular Tumor Diagnosis and Treatment, Beijing Ophthalmology & Visual Sciences Key Lab, Medical Artificial Intelligence Research and Verification Key Laboratory of the Ministry of Industry and Information Technology, Beijing Tongren Hospital, Capital Medical University, 100730 Beijing, China; 2 School of Clinical Medicine, Tsinghua University, 100084 Beijing, China; 3 Chigenovo Co., Ltd., 102206 Beijing, China; 4 Department of Ophthalmology, Peking University Third Hospital, Beijing Key Laboratory of Restoration of Damaged Ocular Nerve, Peking University Third Hospital, 100191 Beijing, China; 5 Department of Ophthalmology, The Third People’s Hospital of Dalian, 116091 Dalian, China; 6 Department of Biomedical Informatics, School of Basic Medical Sciences, Peking University Health Science Center, 100191 Beijing, China; 7 Institute of Systems Biomedicine, Department of Pathology, School of Basic Medical Sciences, Peking University Health Science Center, 100191 Beijing, China

**Keywords:** Translational research, Gene therapy

## Abstract

Bietti crystalline corneoretinal dystrophy is an inherited retinal disease caused by mutations in *CYP4V2*, which results in blindness in the working-age population, and there is currently no available treatment. Here, we report the results of the first-in-human clinical trial (NCT04722107) of gene therapy for Bietti crystalline corneoretinal dystrophy, including 12 participants who were followed up for 180–365 days. This open-label, single-arm exploratory trial aimed to assess the safety and efficacy of a recombinant adeno-associated-virus-serotype-2/8 vector encoding the human CYP4V2 protein (rAAV2/8-h*CYP4V2*). Participants received a single unilateral subretinal injection of 7.5 × 10^10^ vector genomes of rAAV2/8-h*CYP4V2*. Overall, 73 treatment-emergent adverse events were reported, with the majority (98.6%) being of mild or moderate intensity and considered to be procedure- or corticosteroid-related; no treatment-related serious adverse events or local/systemic immune toxicities were observed. Compared with that measured at baseline, 77.8% of the treated eyes showed improvement in best-corrected visual acuity (BCVA) on day 180, with a mean ± standard deviation increase of 9.0 ± 10.8 letters in the 9 eyes analyzed (*p* = 0.021). By day 365, 80% of the treated eyes showed an increase in BCVA, with a mean increase of 11.0 ± 10.6 letters in the 5 eyes assessed (*p* = 0.125). Importantly, the patients’ improvement observed using multifocal electroretinogram, microperimetry, and Visual Function Questionnaire-25 further supported the beneficial effects of the treatment. We conclude that the favorable safety profile and visual improvements identified in this trial encourage the continued development of rAAV2/8-h*CYP4V2* (named ZVS101e).

## Introduction

Bietti crystalline corneoretinal dystrophy (BCD) is an inherited retinal degenerative disorder characterized by the presence of yellow-white crystalline deposits in the retina, which are accompanied by the progressive atrophy of the retinal pigment epithelium (RPE), photoreceptors, and choriocapillaris. Patients with BCD commonly exhibit clinical symptoms in their 20s or 30s, which include progressive night blindness, reduced visual acuity, restricted visual field, and impaired color vision.^1^ According to a natural history study, the best-corrected visual acuity (BCVA) of patients with BCD typically declines by an average of 4.5 letters per year.^2^ By the age of 50–60 years, most individuals with BCD experience severe visual and visual field impairments, resulting in legal blindness.^3^ The worldwide prevalence of BCD is 1/576,000;^4^ however, the condition is more common in East Asia.^5^ In China, the prevalence of retinitis pigmentosa is 1/3784,^6^ with BCD accounting for 15% of these cases;^7^ thus, the prevalence of BCD in China is estimated to be ~1/25,000. Currently, no treatment is available for BCD.

BCD is a specific type of retinitis pigmentosa with autosomal recessive inheritance caused by mutations in *CYP4V2*.^8^ The CYP4V2 protein is produced in multiple body tissues but is particularly abundant in the RPE.^9,10^ CYP4V2 is an omega 3-polyunsaturated medium-chain fatty acid hydroxylase that hydrolyzes docosahexaenoic and eicosapentaenoic acid in the eye.^9^ The photoreceptor outer segment contains large amounts of lipids that can be esterified to form docosahexaenoic acid, eicosapentaenoic acid, and other fatty acids. Physiologically, the RPE engulfs the portion of the photoreceptor outer segment that is undergoing shedding, and the disc lipids are metabolized in the RPE and transferred to the inner segment for the biosynthesis of new discs. This process of lipid recycling promotes disc regeneration while maintaining photoreceptor function. *CYP4V2* mutations disrupt lipid metabolism and RPE-mediated lipid recirculation, thus impairing the regeneration of photoreceptor membrane discs, which leads to photoreceptor damage and, ultimately, retinal degeneration.^11,12^

*RPE65* gene replacement therapy was approved for marketing in 2017, as it represented a notable advance in clinical medicine and offered the potential to correct many other inherited retinal dystrophies (IRDs) caused by mutations that lead to functional impairment, including BCD. Adeno-associated virus (AAV)-mediated gene therapy has shown promising efficacy in several in vivo or in vitro BCD models.^12–15^ In particular, the recombinant AAV2/8–human *CYP4V2* (rAAV-h*CYP4V2*) vector, which produces the functional wild-type human CYP4V2 protein (patent no. CN113106124B), has been found to be efficient in an induced pluripotent stem cells-derived RPE from a patient with BCD as well as in a *Cyp4v3-*knockout mouse model,^14^ providing the basis for a clinical trial for this gene therapy.

Here, we report an investigator-initiated trial of gene replacement therapy for BCD. This is the first clinical trial on BCD worldwide with a focus on assessing the safety and preliminary clinical efficacy of rAAV2/8-h*CYP4V2*, which will offer unprecedented prospects for addressing unmet medical needs in the management of BCD.

## Results

### Participant characteristics and intervention

A total of 12 participants were enrolled in this study; the first 6 participants were followed up for 365 days, and the remaining 6 participants were followed up for 180 days. Their median age was 40 years (SD, 8.2 years; range, 27–50 years), and half of them were female (Table 1). One eye per patient was selected for treatment with rAAV2/8-h*CYP4V2*. The median baseline visual acuity letter score in the treated eyes was 30.6 (Snellen equivalent, ~20/200; Table 1). Most participants exhibited widespread RPE and choriocapillaris atrophy, as well as ellipsoid zone disruption and extensive loss (Supplementary Figs. 1 and 2). The investigational product (rAAV2/8-h*CYP4V2*, also known as ZVS101e*)* was successfully injected into the subretinal space through one or two injection sites (retinotomies) per participant, with the fovea included in the administration area in 3 participants (R004, R007, and R008; Supplementary Fig. 2). The injected viral particles were fully absorbed within 24 hours. In 2 participants, R004 and R008, macular holes developed 1 day after surgery and healed 15 days after surgery (Supplementary Figs. 3 and 4). Participant R005 developed cataracts 1 day after the surgery and underwent lens replacement surgery 9 months after the intervention. Participant R002 was excluded from the efficacy analysis due to an accidental head trauma that occurred 27 days after surgery.

**Table 1:** Demographic and baseline clinical characteristics of the 12 enrolled participants

Participants no.	Gender	Age	*CYP4V2* mutations	Treated eye	ETDRS letters	
					Right	Left
R001	Female	48	c.802-8_810del17insGC	Right	16	61
			c.802-8_810del17insGC			
R002	Female	50	c.802-8_810del17insGC	Right	26	42
			c.992A>C: p.His331Pro			
R003	Female	42	c.802-8_810del17insGC	Right	41	61
			c.1199G>A: p.Arg400His			
R004	Female	29	c.802-8_810del17insGC	Right	59	69
			c.992A>C: p.His331Pro			
R005	Male	36	c.992A>C: p.His331Pro	Right	34	61
			c.992A>C: p.His331Pro			
R006	Male	42	c.992A>C: p.His331Pro	Right	29	46
			c.1020G>A: p.Trp340Ter			
R007	Male	47	c.802-8_810del17insGC	Right	HM	HM
			c.1091-2A>G			
R008	Male	32	c.761A>G,p.His254Arg	Right	41	48
			c.992A>C: p.His331Pro			
R009	Male	27	c.802-8_810del17insGC	Left	57	53
			c.802-8_810del17insGC			
R010	Female	27	c.802-8_810del17insGC	Right	41	53
			c.283G>A, p.Gly95Arg			
R011	Male	42	c.802-8_810del17insGC	Right	9	40
			c.1091-2A>G			
R012	Female	38	c.992A>C: p.His331Pro	Right	44	67
			c.802-8_810del17insGC			

HM was recorded as 2.3 LogMAR according to the scale adopted by Lange et al.^32^ and was recorded as –30 letters in the calculation of the participants’ mean ETDRS letters

*HM* hand motion

### Product safety

73 treatment-emergent adverse events (TEAEs) were reported in the 12 participants, including 24 ocular TEAEs and 49 systemic TEAEs. Ocular TEAEs were consistent with vitrectomy and subretinal injection procedures (Table 2). 2 serious adverse events occurred in 2 participants: R002 experienced a decrease in visual acuity due to head trauma by an accidental fall, and R005 developed cataracts after subretinal injection and underwent standard lens replacement surgery. The most common adverse event was coronavirus disease (COVID-19), followed by hypercholesterolemia and leukocytosis. No suspected unexpected serious adverse reaction was observed, and most of the TEAEs were mild (94.5%) or moderate (4.1%). 5 TEAEs (6.8%), including hyperlipidemia (4/73, 5.5%) and hypercholesterolemia (1/73, 1.4%), were considered to be possibly related to both the investigational product and corticosteroids; 32 TEAEs (43.8%) were considered to be related to corticosteroids; and 23 TEAEs (31.5%) were considered to be related to the procedure. Therefore, our analysis demonstrated that a single unilateral subretinal rAAV2/8-h*CYP4V2* administration is safe and well-tolerated.

**Table 2:** Ocular and systemic TEAEs in the 12 enrolled participants

Parameter	Participants, *n*(%)	Number of events	Severity	Outcome	Related to drug
*Ocular TEAE*					
Conjunctival hyperemia	7(58.3%)	7	Mild	Recovered	Not probable
Anterior chamber flare	6(50.0%)	6	Mild	Recovered	Not probable
Keratic precipitates	3(25.0%)	3	Mild	Recovered	Not probable
Corneal edema	1(8.33%)	1	Mild	Recovered	Not probable
Macular hole	2(16.7%)	2	Mild	Recovered	Not probable
Cataract	1(8.33%)	2	Moderate	Recovered/Ongoing	Not probable
Ocular hypertension	2(16.7%)	2	Mild	Recovered	Not probable
Vision acuity decreased	1(8.33%)	1	Severe	Relieved	Not probable
*Systemic TEAE*					
Hypertriglyceridemia	7(58.3%)	7	Mild	Recovered/Ongoing	Possible/Not probable
Hypercholesteremia	8(66.7%)	10	Mild	Recovered	Possible/Not probable
Leukocytosis	7(58.3%)	9	Mild	Recovered	Not probable
Neutrophil count increased	7(58.3%)	8	Mild	Recovered	Not probable
Head trauma	1(8.33%)	1	Moderate	Recovered	Surely not
Wisdom tooth extraction	1(8.33%)	1	Mild	Resolved	Surely not
COVID-19 infection	11(91.7%)	11	Mild	Recovered	Surely not
Urinary tract infections	1(8.33%)	1	Mild	Recovered	Surely not
Blood urine	1(8.33%)	1	Mild	Recovered	Not probable

*TEAE* treatment-emergent adverse event, *COVID-19* coronavirus disease

### Vector shedding and immunogenicity

**Fig. 1:**
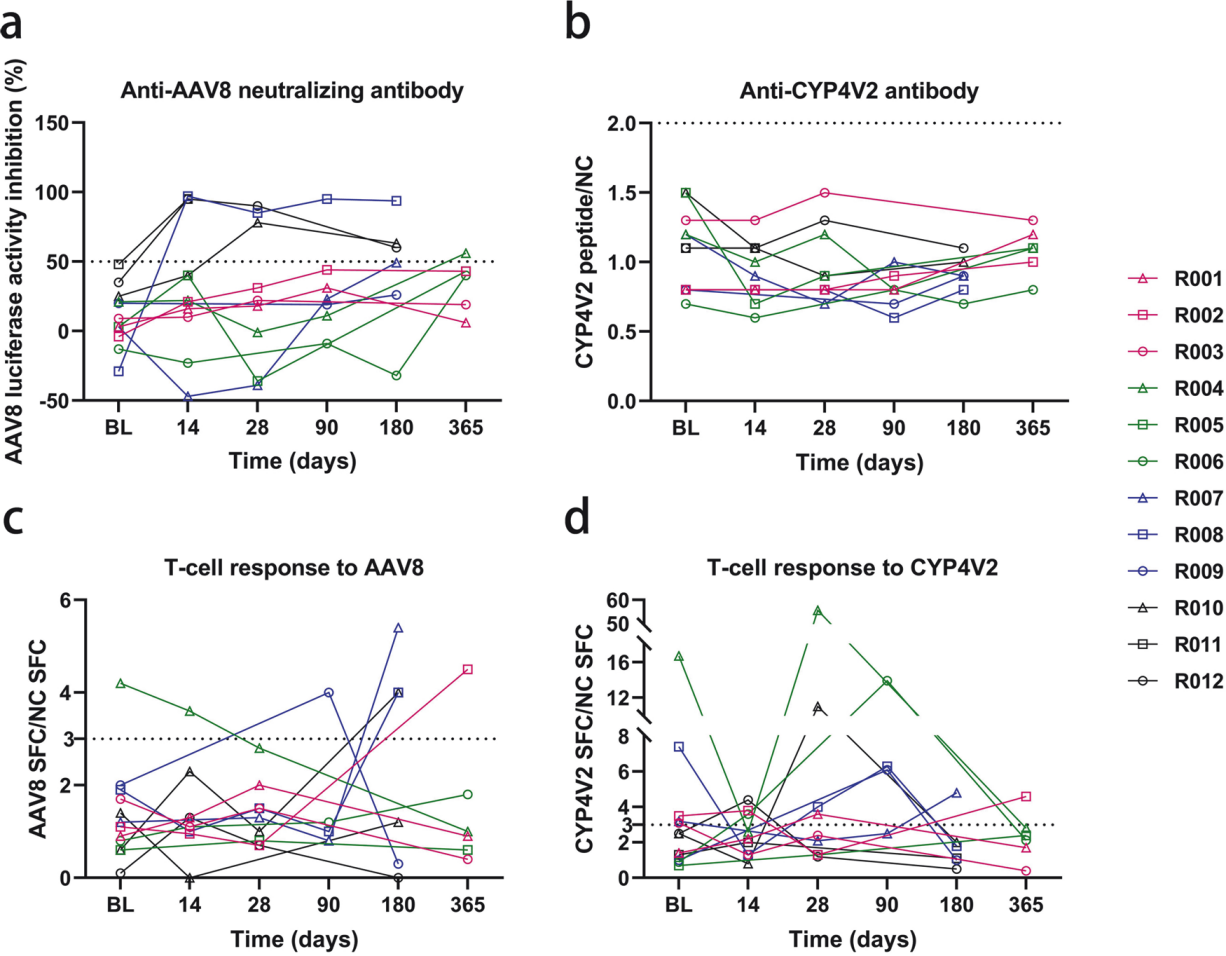
Humoral and T-cell immunity against rAAV2/8-h*CYP4V2.* **a** Serum-neutralizing antibodies against AAV8 before and after treatment. The *Y*-axis represents the 50% fluorescence inhibition rate at a dilution of 1:1000. The 5 participants with the highest neutralizing antibodies at baseline were R011, R012, R010, R004, and R009. **b** Serum anti-CYP4V2 antibody levels before and after treatment. The *Y*-axis represents the ratio of CYP4V2 antigen values detected using enzyme-linked immunosorbent assay (ELISA) to the negative control. **c** T-cell response to AAV8 before and after treatment. The *Y*-axis represents the ratio of AAV8 SFC detected using Elispot to that of the NC. **d** T-cell response to CYP4V2 before and after treatment. The *Y*-axis represents the ratio of CYP4V2 SFC detected using Elispot to that of the NC. The dotted line in the graph represents the positive cut-off value, with values greater than the cut-off value indicating a positive result. SFC spot-forming unit, NC negative control

Viral vector DNA sequences were detected transiently in tears in 5 participants (41.7%) immediately after surgery, and all participants tested negative on the day (D) 2 after surgery (Supplementary Table 1). 4 participants tested positive for vector DNA in the blood, with 2 participants still tested positive 1 year after surgery (Supplementary Table 1). However, the Good Laboratory Practice toxicological results of cynomolgus monkeys showed that the vector DNA in the blood peaked on D4, and by D92, the vector DNA in the blood of all animals was below the lower limit of quantification (Supplementary Fig. 5a). Furthermore, in contrast to the peak of neutralizing antibodies targeting AAV8 in cynomolgus monkeys detected at 15 days after administration, which then declined to baseline levels at 3 months, the trend in neutralizing antibodies varied between participants (Supplementary Fig. 5b–d and Supplementary Table 2). 5 participants showed positive humoral responses to AAV8 during the follow-up period (Fig. 1a, Supplementary Table 2), and 4 participants remained positive at the most recent follow-up (D180 or D365). This highlights the intrinsic variability in the humoral response patterns among patients. All participants showed a negative humoral response to CYP4V2 protein (Fig. 1b). 6 participants showed positive T-cell immune responses to AAV8 at certain visits after administration, with 1 participant (R004) showing positivity before treatment (Fig. 1c). 5 participants showed positive T-cell immune responses to CYP4V2 before treatment (Fig. 1d). This positivity may be due to a cross-reaction with another CYP4V2-like protein or could indicate that the participant may produce a dysfunctional but immunologically detectable CYP4V2 protein. Following treatment, T-cell responses to CYP4V2 tended to be elevated at 1–3 months postoperatively and then declined, with the majority of participants showing a negative T-cell response against CYP4V2 at the latest visit (Fig. 1d). Although some immunogenicity tests were positive, no local or systemic immunotoxic reactions were observed.

### Visual acuity

**Fig. 2:**
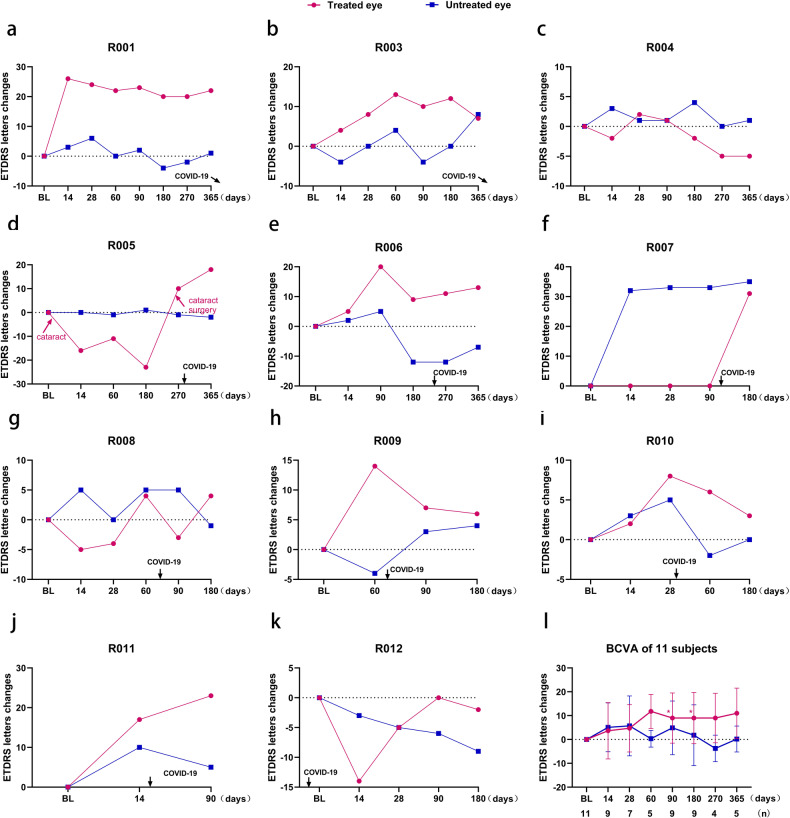
Best-corrected visual acuity (BCVA) changes from baseline in 11 participants. **a**–**k** Changes in early treatment of diabetic retinopathy study (ETDRS) chart letters from baseline at each study visit for participants R001 (**a**) and R003–R012 (**b**–**k**). l Average changes in the BCVA of the treated and untreated eyes from baseline of the 11 participants, excluding the BCVA data during the cataract period (D1-D180) for participant R005. At each visit, the number of treated eyes (*n*) with BCVA values is shown below the *x*-axis. Owing to the COVID-19 pandemic or poor compliance, some participants were unable to participate in the scheduled follow-up, resulting in changes in the number of treated eyes at each follow-up time. *p < 0.05. Data are presented as mean ± SD. BL baseline. Participants R001 and R003 were infected with COVID-19 after 1 year postoperatively, therefore, the arrows point lateral to the x-axis

11 participants were included in the efficacy analysis set, with data from R005 excluded owing to the development and treatment of cataracts (D1-D180) and R011 missing the D180 follow-up; therefore, a total of 9 treated eyes were available for analysis on D180, and 5 were available for analysis at D365. On D180, 77.8% (7/9) of the treated eyes showed significant improvement in BCVA compared with that measured at baseline, with an average gain of 9.0 ± 10.8 letters (mean ± SD) for these 9 eyes (*p* = 0.021) (Fig. 2, Supplementary Table 3). On D365, the BCVA of 80% (4/5) of the treated eyes increased, with an average gain of 11.0 ± 10.6 letters (*p* = 0.125) compared with that measured at baseline in these 5 eyes (Fig. 2, Supplementary Table 3), and 40% (2/5) of the treated eyes achieved a visual acuity improvement of ≥15 letters (Supplementary Table 3). Importantly, in R006, the treated eye that had poorer vision before injection (29 letters vs. 46 letters) showed considerable improvement after treatment, became the dominant eye and had better vision than the untreated eye (38 letters vs. 34 letters) (Supplementary Table 3).

Many participants reported a decline in visual acuity after contracting COVID-19. BCVA results revealed that among the 5 participants (R008–R012) who were infected with COVID-19 within 3 months after surgery or 2 weeks before surgery, 4 participants exhibited deteriorated treatment outcomes, especially participant R012 who contracted COVID-19 2 weeks before the procedure and experienced a significant decline in visual acuity 14 days post-surgery.

Based on the most recent follow-up results (D180 or D365), only 2 participants (R004 and R012) showed a decrease in visual acuity compared to their baseline values. Notably, 180 days after surgery, visual acuity in the treated eye of participant R012 decreased by 2 letters, whereas that in the untreated eye decreased by 9 letters (Fig. 1, Supplementary Table 3). Consequently, it can be inferred that this treatment delayed, to some extent, the visual loss progression and provided benefits to the participant.

Additionally, 54.5% (6/11) of the contralateral untreated eyes exhibited an improvement in vision during follow-up. Compared with that measured at baseline, the average improvement on D180 and D365 for all untreated eyes was 1.8 ± 12.8 letters (*p* = 0.125) and 0.2 ± 5.4 letters (*p* = 0.892), respectively. Notably, participant R007 displayed a significant improvement in the untreated eye as early as 14 days after surgery, with visual acuity improving from hand motion to 2 letters, which exceeded the improvement observed in the treated eye in terms of both timing and significance. Collectively, our results indicated that a significant improvement in visual acuity was achieved in the majority of the treated and contralateral eyes.

### Electroretinogram (ERG)

The dark-adapted (DA) 0.01 b-wave represents rod cell function, whereas the light-adapted (LA) 3.0 b-wave mainly represents cone cell function. The DA 0.01 b-wave was not recorded in 72.7% (8/11) of the treated eyes, and the LA 3.0 b-wave was not recorded in 63.6% (7/11) of the treated eyes at baseline, indicating severe retinal degeneration of these participants (Supplementary Table 4). Following treatment, no significant b-wave amplitude improvement was found in these 11 participants under various light stimulation intensities when compared with those measured at baseline (Supplementary Fig. 6).

### Multifocal ERG (mfERG)

**Fig. 3:**
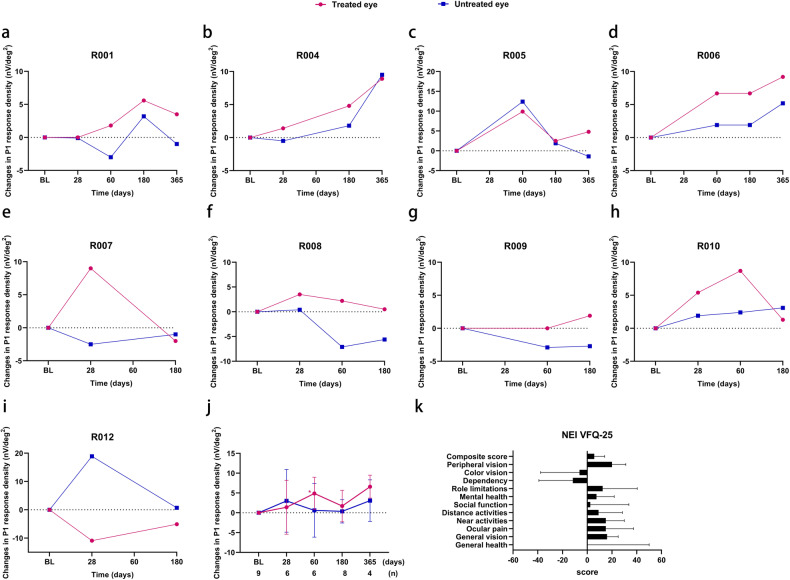
Multifocal electroretinogram (mfERG) and National Eye Institute Visual Function Questionnaire-25 (NEI VFQ-25) changes from baseline. **a**–**j** Changes from baseline in the P1-wave response density of ring 1 of participants R001 (**a**), R004 (**b**), R005 (**c**), R006 (**d**), R007 (**e**), R008 (**f**), R009 (**g**), R010 (**h**), R012 (**i**), and all participants (**j**), as monitored by mfERG. The number of participants with mfERG values (*n*) for each visit is shown below the *x*-axis. **k** Changes from baseline in the composite score and 11 subscale scores for NEI VFQ-25. Scores could not be calculated for the driving subscale because participants did not drive preoperatively or postoperatively. BL baseline. Data are presented as mean ± SD in (**j**) and (**k**)

Our mfERG results revealed an enhancement in the P1-wave response density in central ring 1 after treatment (Fig. 3a–j). Both eyes exhibited an elevation in the P1-wave response density, with more remarkable improvement observed in the treated eyes (Fig. 3a–j). Due to the lack of mfERG examination at baseline for participant R003, mfERG data were available for only 8 participants on D180 and for 4 participants on D365. No significant change was observed in the P1-wave response density of rings 2–5 on D180; however, an improvement was observed on D365 (Supplementary Fig. 7). This discrepancy can be primarily attributed to the fact that data for D365 were only available for 4 participants and the enhanced mfERG responses of these participants were not unique to ring 1.

On D180, 75% (6/8) of the participants displayed an elevation in the P1-wave response density in ring 1. In the treated eyes, the average increment was 1.7 ± 4.0 nV/deg^2^ (*p* = 0.313), whereas in the untreated eyes, it was 0.4 ± 2.9 nV/deg^2^ (*p* = 0.514) (Fig. 3a–j). By D365, 100% (4/4) of the participants exhibited an increase in the P1-wave response density in ring 1. The average increment of the treated eyes in ring 1 was 6.6 ± 2.9 nV/deg^2^ (*p* = 0.125), whereas it was only 3.1 ± 5.2 nV/deg^2^ for the untreated eyes (*p* = 0.625) (Fig. 3). Notably, on D60, the P1-wave response density in ring 1 of the treated eyes was significantly increased, with an increase of 4.9 ± 4.1 nV/deg^2^ compared with that measured at baseline (*p* = 0.043).

### Quality of life

6 participants completed the Visual Function Questionnaire-25 (VFQ-25), 5 of whom were followed up for 180 days after surgery (R007–R010 and R012). In general, a 4~6-point enhancement in the VFQ-25 composite score is considered clinically meaningful and is equivalent to a 15-letter improvement in visual acuity.^16^ On D180, the total composite score increased by 5.7 ± 8.3 points from that at baseline (*p* = 0.313) (Fig. 3k, Supplementary Table 5), indicating a remarkable improvement in self-reported visual function. From baseline to D180, improvement was detected on 8 of the 12 subscales of the questionnaire, including general vision, ocular pain, near activities, distance activities, social function, mental health, role limitations, and peripheral vision. Despite having questionnaire data from only 5 participants, statistically significant improvements were observed in 2 subscales following treatment: general vision (16.0 ± 8.9 points, *p* = 0.046) and peripheral vision (20.0 ± 11.2 points, *p* = 0.046).

### Humphrey visual field and microperimetry

All participants’ visual fields were severely impaired at baseline, with a mean deviation (MD) of the treated eyes of –27.61 ± 6.00 dB, according to the Humphrey visual field test. After treatment, no improvement in MD was observed in treated eyes (Supplementary Fig. 8). Moreover, 55.6% (5/9) of the treated eyes showed an increase in average retinal sensitivity within the 40° range of the retina when tested using microperimetry, whereas 66.7% (6/9) of the untreated eyes showed an improvement in retinal sensitivity (Supplementary Fig. 9). Given that the mfERG central ring showed the most significant improvement, we calculated the mean retinal sensitivity of the central 20° of the retina. On D180, the retinal sensitivity of the treated eyes had improved by 0.26 ± 0.37 dB (*p* = 0.065), that of the contralateral eyes improved by 0.15 ± 0.98 dB (*p* = 0.202), and 77.8% (7/9) of the participants showed an increase in mean retinal sensitivity in both eyes. On D365, the mean retinal sensitivity had increased in the treated eyes (0.11 ± 0.69 dB) compared with that at baseline (*p* = 0.892), whereas the retinal sensitivity of the untreated eye on the opposite side had decreased (–0.39 ± 1.25 dB, *p* = 1.000; Supplementary Table 6). These results suggest that rAAV2/8-h*CYP4V2* administration improves the sensitivity of the central retina.

### Fundus autofluorescence (FAF) and contrast sensitivity test

Owing to the severe RPE atrophy in the participants, resulting in its inability to emit sufficient signals in the FAF light, recording FAF images before and after treatment was challenging (Supplementary Fig. 10). However, we observed that FAF imaging could be more easily implemented in the eyes of participants R001–R007 after treatment. Participants could not read any of the images before and after treatment, indicating no change in contrast sensitivity.

## Discussion

To our knowledge, this study provides the first 1-year follow-up results of a gene therapy clinical trial for BCD, which is caused by mutations in *CYP4V2* as the most common disease-causing gene for IRDs in China.^17^ Subretinal injection of rAAV2/8-h*CYP4V2* showed a favorable safety profile, without inflammatory responses or treatment-related serious adverse events, and both subjective and objective measurements of visual function continued to improve within 1 year after administration.

Among most participants, visual acuity improved from as early as D14 and remained stable for at least 1 year. Compared with that measured at baseline, the mean change in BCVA was 11.0 letters in 5 participants on D365. Compared to the natural history of progression of BCD (–4.5 letters/year),^2^ the participants of this study showed an average improvement of 15.5 letters, indicating significant clinical improvement. There was also a slight improvement in BCVA detected in the untreated contralateral eye, which is consistent with reports from several previous clinical trials of IRD gene therapy and may be due to visual cortex activation, brain plasticity, or vector shedding in the contralateral eye.^18–23^ Unlike other participants, BCVA in participant R007’s treated eye improved from hand motion to 1 letter on D180. We hypothesize two reasons for this delayed improvement. First, this patient had the most severe retinal degeneration and the poorest BCVA among 12 participants at baseline. Therefore, the recovery time for the local injury caused by the surgery may have been relatively longer, resulting in a gradual improvement in BCVA that only became apparent 180 days after the procedure. Second, the participant reported improvement in vision and brightness of the eye after the surgery. However, the current lack of more sensitive testing measures makes it challenging to accurately assess such improvements in off-chart visual acuity; thus, it is likely that the improvement only becomes detectable when it reaches a certain threshold. According to the latest follow-up data, only 1 participant (R004) exhibited a lack of visual benefit from the intervention. There are three possible reasons for this observation. First, compared to other participants, participant R004 has a greater number of surviving photoreceptor cells and RPE cells, and the macular hole generated during surgery may have resulted in the leakage of vectors into the vitreous cavity, leading to insufficient vector delivery. Second, the macular hole caused structural damage to the fovea, with thinning of the foveal thickness persisting at 1 year postoperatively (Supplementary Fig. 3). Third, participant R004 had a pre-existing T-cell response against both AAV8 and CYP4V2, which was significantly higher than that in the other participants; thus, this immune response may have reduced or eliminated the AAV virus or CYP4V2 protein, resulting in retinal toxicity.^24,25^

Importantly, we found that COVID-19 infection and the presence of AAV8-neutralizing antibodies may have negative impacts on the vision benefit obtained with this gene therapy. COVID-19 infection resulted in a reduction in visual acuity in the treated eyes of several participants. However, the mechanism underlying this decline remains elusive. We speculate an involvement of non-specific immune memory following SARS-CoV-2 infection, which may have conferred a temporary boost in immune responses against pathogens other than SARS-CoV-2 that contributed to AAV8 clearance and inflammation in the rAAV2/8-h*CYP4V2* administration area.^26^ Consequently, the potential impacts of other systemic viral infections must be considered when selecting patients for future studies. Although all participants were negative for AAV8-neutralizing antibodies at baseline, 4 out of the 5 participants with the highest baseline values tested positive for neutralizing antibodies after surgery (Fig. 1a); only 1 of these 4 participants exhibited a visual acuity gain of more than 5 letters after treatment, implying a potential negative impact of neutralizing antibodies on efficacy. However, this possibility is not supported by the case of participant R011, who had the highest baseline neutralizing antibody level among the 12 participants but demonstrated a significant improvement in BCVA of 23 letters. Similar phenomena were also observed in a clinical trial of LUXTURNA, a commercially available gene therapy drug for *RPE65*-related IRDs, where one participant who showed high-titer neutralizing antibodies prior to treatment did, in fact, benefit from the treatment.^27^ These findings suggest that in ocular gene therapy trials with subretinal administration, it may be advisable to limit patients according to the baseline measured titer of AAV neutralizing antibodies and that the current titer of 1:1000 may, in fact, be too high and should be reduced appropriately. However, it should be noted that AAV-neutralizing antibodies do not necessarily preclude the potential benefits of the treatment for the participants.

Multifocal ERG is an objective measure of retinal function that primarily reflects the bioelectric activity of photoreceptors and bipolar cells.^28^ In this study, we observed a marked improvement in the P1-wave response density of ring 1. This finding strongly indicates an improved function of one or more cell populations within 10° of the macula centers. Moreover, this observation provides compelling evidence that the enhancement of BCVA cannot be attributed to a placebo effect. Consistent with the mfERG results, microperimetry examination showed that the improvement in retinal sensitivity was more pronounced within 20° of the macula than within 40°. The participants showed an improvement, albeit not significant, in mean retinal sensitivity compared with that at baseline. However, no significant improvement was observed in the mfERG rings 2–5, full-field ERG, or Humphrey visual field. This lack of improvement in the peripheral macular and retinal areas, which were already severely degenerated prior to treatment, suggests that gene therapy is ineffective in treating a retina lacking the target cells. These findings demonstrate the sensitivity of mfERG and microperimetry for measuring macular function and highlight its potential as an efficacy outcome in gene therapy clinical trials for IRDs.

Patient-reported outcomes have been increasingly recognized to have value in interpreting the results of clinical trials, and the VFQ-25 has shown a strong association with improvements in visual acuity. In our study, we noted an improvement in the VFQ-25 scores, with significant improvements observed in general and peripheral vision in particular. This improvement was notable because we only treated the participants’ poorer eyes and thus anticipated that improvement in visual acuity of the treated eyes would have less impact on subjective experiences, considering that the dominant eye with good vision usually governs daily activities. This phenomenon can be partly attributed to the augmentation of visual function in the contralateral eye, and it is also possible that the treated eye shifted to become the dominant eye, presumably owing to its improved function.

In this trial, 2 adverse events, hypertriglyceridemia and hypercholesterolemia, were possibly related to the treatment and corticosteroids. Patients with BCD have systemic lipid metabolism disorders, and metabolic studies on cells cultured from patients with BCD have shown abnormally high stores of triglycerides and cholesterol.^29,30^ In line with this notion, approximately 30% of the patients with BCD in our outpatient clinic exhibited elevated serum triglyceride or cholesterol levels. Therefore, we also suspect that these adverse events may be related to the fluctuation in patients’ serum lipids levels (Supplementary Table 7), although the baseline values were within the normal range. We do not consider that the observed lipid abnormalities are attributed to vector shedding in the blood, as vector DNA was not detected in the blood of most participants with hypertriglyceridemia or hypercholesterolemia.

This single-arm study bore some limitations. First, as this was a single-dose study, the efficacy and safety of administering additional doses were not investigated. Second, the limited number of participants prevented identifying which patients could benefit the most from the treatment. Thirdly, due to ethical considerations, sham injections were not employed in this study, and thus the treatment was conducted in an open-label manner rather than utilizing an optimal randomized controlled design. Finally, some efficacy evaluation indicators in the study design, such as contrast sensitivity tests, are not suitable for patients with late-stage BCD who were eligible for inclusion in this study.

In summary, the results of this study clearly demonstrate that subretinally administrated rAAV2/8-h*CYP4V2* is safe and efficient for patients with BCD. Our study provides the foundation for gene-therapy approaches for BCD treatment and encourages future exploration of the safety and efficacy of rAAV2/8-h*CYP4V2*, bringing hope to a vast number of patients with BCD.

## Materials and methods

### Trial implementation and supervision

This investigator-initiated trial was conducted at Beijing Tongren Hospital, Capital Medical University, China, under the approval of the Ethics Committee of Beijing Tongren Hospital, Capital Medical University, and was conducted in accordance with Good Clinical Practice guidelines and the tenets of the Declaration of Helsinki. Written informed consent was obtained from all the participants. The principal investigator supervised the trial and was responsible for data collection and analysis. All animal experiments and procedures were ethically approved by the Institutional Animal Care and Use Committee at Joinn Laboratories (No. ACU21-2906).

### Trial design

This was an open-label, single-arm clinical trial that involved 12 participants with BCD to assess the safety and preliminary efficacy of rAAV2/8-h*CYP4V2* gene replacement therapy (ClinicalTrials.gov identifier: NCT04722107). For each patient, the eye with the poorer function was selected for subretinal administration of rAAV2/8-h*CYP4V2*. Patients were evaluated at designated time points for up to 2 years. Adverse events and immune responses were monitored, and efficacy data for all evaluable participants were analyzed both individually and in a pooled manner.

### rAAV2/8-hCYP4V2 virus

The rAAV2/8-h*CYP4V2* vector, also known as ZVS101e, was developed by Chigenovo Co., Ltd., Beijing, China. This construct contains a recombinant AAV serotype-2/8 vector, which encodes the human *CYP4V2* gene. The vector genome comprises 3.3 kb of single-stranded DNA with AAV2 inverted terminal repeats at both ends and the target gene expression cassette in the middle. Gene expression is driven by the CAG promoter. The viral product is produced by Obio Technology Co., Ltd., Shanghai, China, in compliance with Good Manufacturing Practices. The virus was generated using triple-plasmid transfection and was purified through affinity chromatography and ion exchange chromatography to ensure the removal of impurities. Further details of viral and vector preparation were provided in the “Materials and methods” section of the Supplementary Materials.

### Inclusion and exclusion criteria

The recruited participants had to meet the following criteria: (1) late-stage BCD caused by *CYP4V2* biallelic mutations with an age of ≥18 years; (2) BCVA hand motion to 20/63; (3) the eye with poor visual function is selected as the study eye in each participant; and (4) agreement to use effective contraception during the trial for both participants and their spouses. The exclusion criteria included: (1) insufficient viable retinal cells or macular retinal thickness ≤100 μm; (2) prior intraocular surgery in the study eye, such as vitrectomy, photodynamic therapy, or other retinal laser therapies; (3) prior use of drugs that could affect the experimental observation within 6 months of screening, such as ranibizumab, bevacizumab, aflibercept, and conbercept; (4) being administered or requiring systemic drugs that may cause ocular toxicity, such as psoralen, risedronate, or tamoxifen; (5) AAV neutralizing antibody titer >1:1000; (6) participation in any drug or medical-device clinical trial within 3 months prior to enrollment; and (7) pregnant or lactating women.

### Procedures

To minimize the inflammation caused by surgery or potential immune responses to the vector/transgene, prednisone was administered at a dose of 1 mg/kg per day, starting 3 days before injection and continued for 3 days, followed by 0.5 mg/kg per day for an additional 7 days.

All participants underwent a standard 25-gauge, three-port, flat vitrectomy under general anesthesia and were administered rAAV2/8-h*CYP4V2* via subretinal injection. First, sterile physiological saline was injected using a 38-gauge cannula (3255, MedOne Surgical, Sarasota, FL, USA) to create local retinal detachments (pre-blebs). Subsequently, 150 μL of rAAV2/8-h*CYP4V2* (7.5 × 10^10^ viral genomes) was injected into the pre-bleb through the same retinotomy. Injection sites (retinotomies) were located near the superior temporal vascular arcades. 2 retinotomies were used in some participants to avoid high subretinal bleb. The injection pressure was controlled between 10 and 25 psi using a foot pedal. According to the routine clinical medication plan after vitrectomy, local glucocorticoids and antibiotics were administered to prevent inflammation and infection (see the “Materials and methods” section of the Supplementary Materials for further details). All procedures were performed by the same surgeon.

### Outcome measures

The primary objective of this study was to assess the safety and tolerability of a single subretinal injection of rAAV2/8-h*CYP4V2* in patients with BCD as measured by the incidence of TEAEs within 2 years. These assessments included vital signs, electrocardiographs, physical examination, clinical laboratory testing, immunogenicity monitoring, ophthalmic examination and vector shedding. The secondary objective was to evaluate the efficacy of rAAV2/8-h*CYP4V2* by calculating the change in efficacy indicators after treatment compared to those at baseline, including BCVA, FAF, optical coherence tomography (OCT), OCT angiography (OCTA), Humphrey visual field, microperimetry, ERG, mfERG, contrast sensitivity and color vision.

#### Vector shedding

To assess the level of AAV vector shedding after subretinal injection in 12 participants, 5–10 μL tears from the treated and untreated eyes and 2 mL whole blood were collected. Genomic DNA was extracted from these samples, and the concentration of the AAV8 vector was quantified using a real-time quantitative polymerase chain reaction assay. The detection limit was 400 genome copies/μL.

#### Immunogenicity

Serum samples were collected to evaluate the humoral responses to the AAV8 vector and CYP4V2. The anti-CYP4V2 antibody titer was quantified using an enzyme-linked immunoassay targeting CYP4V2 antigen (see the “Materials and methods” section of the Supplementary Materials for assay details). AAV8 neutralizing antibody titers were quantified using a cell-based assay. Prior to transduction into HEK293A cells, serum samples were diluted (1:20, 1:1000, and 1:10,000) and co-cultured with an AAV reporter (AAV8-Luciferase, see the Materials and Methods section of the Supplementary Materials for details). A positive result (AAV neutralizing antibody >1:1000) was defined when luciferase activity inhibition was ≥50% at a dilution of 1:1000. To assess T-cell responses to the AAV8 vector and the transgene *CYP4V2*, peripheral blood mononuclear cells (PBMCs) were obtained from the 12 participants and interferon-γ-secreting cells were quantified by comparing PBMCs co-cultured with either an AAV8 capsid peptide library or CYP4V2 peptide library, with negative controls being incubated solely with culture media. AAV8 peptide library (JPT Peptide Technologies, Q8TQF8) contains 182 peptides from the AAV8 capsid protein. The CYP4V2 peptide library (Genscript, C6730FL080) contains 261 peptides derived from the CYP4V2 protein. Results were considered positive when the ratio of the sample to be tested to the control sample was ≥3.

#### BCVA

After standardized refractive correction, visual acuity was measured using the ETDRS charts. The test was conducted at a distance of 4 metres or 1 metre. For each participant, the number of letters correctly read was recorded.

#### ERG and mfERG

ERG (RETI-port^®^, Roland, Wiesbaden, Germany) and mfERG (Diagnosys LLC, Lowell, MA, USA) recordings were conducted following the standards of the International Society for Clinical Electrophysiology of Vision. Both eyes were examined after pupillary dilation and dark adaptation. The participants underwent ERG testing under both dark-adapted and light-adapted conditions. Dark-adapted responses were assessed using stimuli of 0.01, 3.0, and 10.0 cd s/m^2^. Subsequently, the participants were exposed to a rod-saturating background of 30 cd/m^2^ for 10 min to achieve light adaptation. Light-adapted responses were then recorded at a stimulus intensity of 10.0 cd.s/m^2^. For mfERG, the stimulus consisted of an array of 103 hexagons scaled according to eccentricity, and the stimulus array was divided into 7 concentric rings around the central fovea at 0°, 2.7°, 6.3°, 11°, 16.8°, 23.3°, and 28.4°. The luminance of the white hexagons was 1000 cd/m^2^, and the P1-wave response density (nV/deg^2^) of each ring was recorded for each participant.

#### Humphrey visual field

All participants underwent a visual field test on Humphrey’s Field Analyzer 840 (Carl Zeiss-Humphrey Systems, Dublin, CA, USA) using the 24-2 SITA-Fast strategy. The visual fields were considered satisfactory if false-positive and false-negative errors did not exceed 33% and fixation errors did not exceed 20%.^31^

#### Microperimetry

Microperimetry assessments were conducted for both eyes in a dark room using the MP-3 system (NIDEK, Gamagori, Japan). All participants used a white Goldmann III stimulus with a duration of 200 ms, a white background luminance of 31.4 asb, a maximum stimulus luminance of 34 dB, and a 4-2 (fast) threshold strategy. The stimulus grid comprised 100 points covering the central 40° of the retina. The stimulus points measured at baseline were stored in the software and mapped to the same area of the retina in the follow-up test.

#### FAF

Ultra-wide field FAF images were obtained using a ZEISS CLARUS 500 system (Carl Zeiss Meditec, Jena, Germany). The tests were performed after pupil dilatation.

#### Quality of life

Quality of life was assessed using the National Eye Institute (NEI) VFQ-25. This questionnaire consists of 25 questions with 1 general health subscale and 11 vision subscales, including general vision, ocular pain, near and distance activities, social function, mental health, role limitations, dependency, driving, color vision, and peripheral vision. The mean scores of the 12 vision-related subscales and the overall composite score of NEI VFQ-25 were calculated upon completion. The overall composite score represented the average of the scores from the vision-related subscales, excluding the general health-rating question. Since quality of life had not been included for assessment in the original version of the protocol, the questionnaire was administered to participants R007–R012 only.

#### Contrast sensitivity

Contrast sensitivity was evaluated using the Optec FVA test (StereoOptical, Chicago, LL, USA) under mesopism (3.5 cd/m^2^) at spatial frequencies of 1.5, 3, 6, 12, and 18 cycles per degree.

### Statistical analysis

All participants receiving rAAV2/8-h*CYP4V2* treatment were included in the safety analysis. The incidence of various adverse events (ocular or system) was summarized. Changes in efficacy indicators relative to those at baseline were calculated for each visit after treatment. The Wilcoxon signed-rank test was used to evaluate the changes in BCVA, mfERG, ERG, Humphrey visual field, microperimetry, contrast sensitivity, VFQ-25 scores, and FAF relative to those measured at baseline at each time point after vector administration. A two-sided *p* ≤ 0.05 denoted statistical significance. All statistical analyses were performed using SPSS v25.0.

### Supplementary information

Supplementary Materials

Clinical Trial Protocol

## Data Availability

The individual de-identified participants data, which form the basis of the results of this report, are available in the article and Supplementary Materials. Some data supporting the results of this study can be obtained from Chigenovo Co., Ltd. All requests for raw and analytical data will be promptly reviewed by a Chigenovo delegate and trial organizer to determine if there are any intellectual property or confidentiality restrictions. Any data that can be shared will be released via a data use agreement.

## References

[1] Yuzawa M, Mae Y, Matsui M (1986). Bietti’s crystalline retinopathy. Ophthalmic Paediatr. Genet..

[2] Murakami Y (2021). Genotype and long-term clinical course of Bietti crystalline dystrophy in Korean and Japanese patients. Ophthalmol. Retina.

[3] Osman Saatci A, Can Doruk H (2014). An overview of rare and unusual clinical features of Bietti’s crystalline dystrophy. Med. Hypothesis Discov. Innov. Ophthalmol..

[4] Hanany M, Rivolta C, Sharon D (2020). Worldwide carrier frequency and genetic prevalence of autosomal recessive inherited retinal diseases. Proc. Natl Acad. Sci. USA.

[5] Ng DS, Lai TY, Ng TK, Pang CP (2016). Genetics of Bietti crystalline dystrophy. Asia Pac. J. Ophthalmol..

[6] Hu D (1987). Prevalence and mode of inheritance of major genetic eye diseases in China.pdf. J. Med. Genet..

[7] Gao FJ (2019). Genetic and clinical findings in a large cohort of Chinese patients with suspected retinitis pigmentosa. Ophthalmology.

[8] Li A (2004). Bietti crystalline corneoretinal dystrophy is caused by mutations in the novel gene CYP4V2. Am. J. Hum. Genet..

[9] Nakano M (2012). CYP4V2 in Bietti’s crystalline dystrophy: ocular localization, metabolism of ω-3-polyunsaturated fatty acids, and functional deficit of the p.H331P variant. Mol. Pharmacol..

[10] Jia R (2023). AAV-mediated gene-replacement therapy restores viability of BCD patient iPSC derived RPE cells and vision of Cyp4v3 knockout mice. Hum. Mol. Genet..

[11] Hata M (2018). Reduction of lipid accumulation rescues Bietti’s crystalline dystrophy phenotypes. Proc. Natl Acad. Sci. USA.

[12] Zhang Z (2020). PSCs reveal PUFA-provoked mitochondrial stress as a central node potentiating RPE degeneration in Bietti’s crystalline dystrophy. Mol. Ther..

[13] Qu B (2020). Treating Bietti crystalline dystrophy in a high-fat diet-exacerbated murine model using gene therapy. Gene Ther..

[14] Jia R (2022). AAV-mediated gene replacement therapy restores viability of BCD patient iPSC derived RPE cells and vision of Cyp4v3 knockout mice. Hum. Mol. Genet..

[15] Wang JH (2022). AAV2-mediated gene therapy for Bietti crystalline dystrophy provides functional CYP4V2 in multiple relevant cell models. Sci. Rep..

[16] Sun~er IJ (2009). Responsiveness of NEI VFQ-25 to changes in visual acuity in neovascular AMD: validation studies from two phase 3 clinical trials. Investig. Opthalmol. Visual Sci..

[17] Liu X (2021). Molecular diagnosis based on comprehensive genetic testing in 800 Chinese families with non-syndromic inherited retinal dystrophies. Clin. Exp. Ophthalmol..

[18] Calkins DJ (2021). Biodistribution of intravitreal (lenadogene) nolparvovec gene therapy in nonhuman primates. Mol. Ther. Methods Clin. Dev..

[19] Ashtari M (2011). The human visual cortex responds to gene therapy-mediated recovery of retinal function. J. Clin. Investig..

[20] MacLaren RE (2014). Retinal gene therapy in patients with choroideremia: initial findings from a phase 1/2 clinical trial. Lancet.

[21] Maguire AM (2009). Age-dependent effects of RPE65 gene therapy for Leber’s congenital amaurosis: a phase 1 dose-escalation trial. Lancet.

[22] Bainbridge JW (2015). Long-term effect of gene therapy on Leber’s congenital amaurosis. N. Engl. J. Med..

[23] Jacobson SG (2012). Gene therapy for Leber congenital amaurosis caused by RPE65 mutations: safety and efficacy in 15 children and adults followed up to 3 years. Arch. Ophthalmol..

[24] Vandenberghe LH (2011). Dosage thresholds for AAV2 and AAV8 photoreceptor gene therapy in monkey. Sci. Transl. Med..

[25] Chung SH (2021). Host immune responses after suprachoroidal delivery of AAV8 in nonhuman primate eyes. Hum. Gene Ther..

[26] Dan JM (2021). Immunological memory to SARS-CoV-2 assessed for up to 8 months after infection. Science.

[27] Bennett J (2016). Safety and durability of effect of contralateral-eye administration of AAV2 gene therapy in patients with childhood-onset blindness caused by RPE65 mutations: a follow-on phase 1 trial. Lancet.

[28] Hood DC, Frishman LJ, Saszik S, Viswanathan S (2002). Retinal origins of the primate multifocal ERG: implications for the human response. Investig. Ophthalmol. Visual Sci..

[29] Lai TY (2010). Alterations in serum fatty acid concentrations and desaturase activities in Bietti crystalline dystrophy unaffected by CYP4V2 genotypes. Investig. Ophthalmol. Visual Sci..

[30] Lee J (2001). The metabolism of fatty acids in human Bietti crystalline dystrophy. Investig. Ophthalmol. Visual Sci..

[31] Birt CM (1997). Analysis of reliability indices from Humphrey visual field tests in an urban glaucoma population. Ophthalmology.

[32] Lange C (2009). Resolving the clinical acuity categories “hand motion” and “counting fingers” using the Freiburg Visual Acuity Test (FrACT). Graefes Arch. Clin. Exp. Ophthalmol..

