# Supplementary material for: Gene replacement therapy in Bietti crystalline corneoretinal dystrophy: an open-label, single-arm, exploratory trial
Source: Signal Transduct Target Ther. 2024 Apr 24;9:95. doi: 10.1038/s41392-024-01806-3 (PMC11039457; doi:10.1038/s41392-024-01806-3)
Supplement: Supplementary file 1 — Supplementary Materials [file 41392_2024_1806_MOESM1_ESM.docx]

Supplementary Materials for

Gene Replacement Therapy in Bietti Crystalline Corneoretinal Dystrophy: An Open-label, Single-arm, Exploratory Trial

Jinyuan Wang^1,2^, Jinlu Zhang^3^, Shicheng Yu^4^, Hongyan Li^3^, Shaohong Chen^3^, Jingting Luo^1^, Haibo Wang^1,5^, Yuxia Guan^3^, Haihan Zhang^1^, Shiyi Yin^1^, Huili Wang^3^, Heping Li^3^, Junle Liu^3^, Jingyuan Zhu^1^, Qiong Yang^1^, Ying Sha^1^, Chuan Zhang^1^, Yuhang Yang^1^, Xuan Yang^1^, Xifang Zhang^1^, Xiuli Zhao^1^, Likun Wang^6, 7*^, Liping Yang^4*^, Wenbin Wei^1*^

Correspondence to: wanglk@pku.edu.cn, alexlipingyang@bjmu.edu.cn, [weiwenbintr@163.com](mailto:weiwenbintr@163.com)

**This PDF file includes:**

Materials and Methods

Discussion

Figures S1–S10

Tables S1–S7

References
**Other Supplementary Materials for this manuscript include the following:**

Data S1. T-cell immune responses to AAV8 and CYP4V2

# Materials and Methods

## Vector production

The virus was generated using triple-plasmid transfection of HEK293 cells and the culture supernatant was harvested 72 hours after transfection. To produce the virus, a 1/10 volume of lysate and nuclease was added and incubated for 2 hours at 37°C. Cellular debris was removed by sequential depth capsule filters. Affinity chromatography was employed to purify the AAV. The product underwent further purification using ion-exchange column chromatography with NaCl salt gradient elution. Finally, the AAV8 vector product was formulated in 10 mM phosphate buffer, 150 mM NaCl, and 0.001% P188 (pH 7.3), which was subjected to 0.22 µm sterile filtration to obtain the drug substance.

The drug substance was diluted to the target titer in a biosafety cabinet, filtered with 0.22 μm Millipak 20, and then packaged into the final product. The labeled product was placed in chilled cartons and stored in a freezer at ≤–70°C.

The purity of AAV full capsids was assessed using analytical ultra-centrifugation. The titer of vector genomes and vector particles was determined using quantitative polymerase chain reaction and enzyme-linked immunosorbent assay (ELISA), respectively. Quality control included assays for virus monomer purity, capsid protein purity, residual host cell protein, residual host cell DNA, sterility test, bacterial endotoxin, *Mycoplasma*, rcAAV, sub-visible particles, visible particles, and appearance. The results of virus quality control assays are shown in the table below:

| Vector genomes titer | 3.0 × 10^12^ vg/ml |
| --- | --- |
| Virus monomer purity | ≥90.0% |
| AAV full capsids purity (analytical ultra-centrifugation) | 55.6% |
| Residual host cell protein | ≤100 ng/dose |
| Residual host cell DNA | ≤10 ng/dose |
| Sterility | Conformed |
| *Mycoplasma* | Negative |
| rcAAV | Negative |
| Bacterial endotoxin | <2.5 EU/ml |

## Vector sequence

The ZVS101e (rAAV2/8-h*CYP4V2*) genome comprises approximately 3 kb of single-stranded DNA, including the AAV2 inverted terminal repeat at both ends and the target gene expression frame in between, which consists sequentially of the *CAG* promoter, Kozak sequence, coding region of the h*CYP4V2* gene, and the bGH transcriptional terminator, with the following sequence:

CCATTGACGTCAATAATGACGTATGTTCCCATAGTAACGCCAATAGGGACTTTCCATTGACGTCAATGGGTGGAGTATTTACGGTAAACTGCCCACTTGGCAGTACATCAAGTGTATCATATGCCAAGTACGCCCCCTATTGACGTCAATGACGGTAAATGGCCCGCCTGGCATTATGCCCAGTACATGACCTTATGGGACTTTCCTACTTGGCAGTACATCTACGTATTAGTCATCGCTATTACCATGGTCGAGGTGAGCCCCACGTTCTGCTTCACTCTCCCCATCTCCCCCCCCTCCCCACCCCCAATTTTGTATTTATTTATTTTTTAATTATTTTGTGCAGCGATGGGGGCGGGGGGGGGGGGGGGGCGCGCGCCAGGCGGGGCGGGGCGGGGCGAGGGGCGGGGCGGGGCGAGGCGGAGAGGTGCGGCGGCAGCCAATCAGAGCGGCGCGCTCCGAAAGTTTCCTTTTATGGCGAGGCGGCGGCGGCGGCGGCCCTATAAAAAGCGAAGCGCGCGGCGGGCGGGAGTCGCTGCGACGCTGCCTTCGCCCCGTGCCCCGCTCCGCCGCCGCCTCGCGCCGCCCGCCCCGGCTCTGACTGACCGCGTTACTCCCACAGGTGAGCGGGCGGGACGGCCCTTCTCCTCCGGGCTGTAATTAGCGCTTGGTTTAATGACGGCTTGTTTCTTTTCTGTGGCTGCGTGAAAGCCTTGAGGGGCTCCGGGAGGGCCCTTTGTGCGGGGGGAGCGGCTCGGGGCTGTCCGCGGGGGGACGGCTGCCTTCGGGGGGGACGGGGCAGGGCGGGGTTCGGCTTCTGGCGTGTGACCGGCGGCTCTAGAGCCTCTGCTAACCATGTTCATGCCTTCTTCTTTTTCCTACAGCTCCTGGGCAACGTGCTGGTTATTGTGCTGTCTCATCATTTTGGCAAAGAATTGGATCGGTACCGAGGAGATCTGCCACCATGGCGGGGCTCTGGCTGGGGCTCGTGTGGCAGAAGCTGCTGCTGTGGGGCGCGGCGAGTGCCCTTTCCCTGGCCGGCGCCAGTCTGGTCCTGAGCCTGCTGCAGAGGGTGGCGAGCTACGCGCGGAAATGGCAGCAGATGCGGCCCATCCCCACGGTGGCCCGCGCCTACCCACTGGTGGGCCACGCGCTGCTGATGAAGCCGGACGGGCGAGAATTTTTTCAGCAGATCATTGAGTACACAGAGGAATACCGCCACATGCCGCTGCTGAAGCTCTGGGTCGGGCCAGTGCCCATGGTGGCCCTTTATAATGCAGAAAATGTGGAGGTAATTTTAACTAGTTCAAAGCAAATTGACAAATCCTCTATGTACAAGTTTTTAGAACCATGGCTTGGCCTAGGACTTCTTACAAGTACTGGAAACAAATGGCGCTCCAGGAGAAAGATGTTAACACCCACTTTCCATTTTACCATTCTGGAAGATTTCTTAGATATCATGAATGAACAAGCAAATATATTGGTTAAGAAACTTGAAAAACACATTAACCAAGAAGCATTTAACTGCTTTTTTTACATCACTCTTTGTGCCTTAGATATCATCTGTGAAACAGCTATGGGGAAGAATATTGGTGCTCAAAGTAATGATGATTCCGAGTATGTCCGTGCAGTTTATAGAATGAGTGAGATGATATTTCGAAGAATAAAGATGCCCTGGCTTTGGCTTGATCTCTGGTACCTTATGTTTAAAGAAGGATGGGAACACAAAAAGAGCCTTCAGATCCTACATACTTTTACCAACAGTGTCATCGCTGAACGGGCCAATGAAATGAACGCCAATGAAGACTGTAGAGGTGATGGCAGGGGCTCTGCCCCCTCCAAAAATAAACGCAGGGCCTTTCTTGACTTGCTTTTAAGTGTGACTGATGACGAAGGGAACAGGCTAAGTCATGAAGATATTCGAGAAGAAGTTGACACCTTCATGTTTGAGGGGCACGATACAACTGCAGCTGCAATAAACTGGTCCTTATACCTGTTGGGTTCTAACCCAGAAGTCCAGAAAAAAGTGGATCATGAATTGGATGACGTGTTTGGGAAGTCTGACCGTCCCGCTACAGTAGAAGACCTGAAGAAACTTCGGTATCTGGAATGTGTTATTAAGGAGACCCTTCGCCTTTTTCCTTCTGTTCCTTTATTTGCCCGTAGTGTTAGTGAAGATTGTGAAGTGGCAGGTTACAGAGTTCTAAAAGGCACTGAAGCCGTCATCATTCCCTATGCATTGCACAGAGATCCGAGATACTTCCCCAACCCCGAGGAGTTCCAGCCTGAGCGGTTCTTCCCCGAGAATGCACAAGGGCGCCATCCATATGCCTACGTGCCCTTCTCTGCTGGCCCCAGGAACTGTATAGGTCAAAAGTTTGCTGTGATGGAAGAAAAGACCATTCTTTCGTGCATCCTGAGGCACTTTTGGATAGAATCCAACCAGAAAAGAGAAGAGCTTGGTCTAGAAGGACAGTTGATTCTTCGTCCAAGTAATGGCATCTGGATCAAGTTGAAGAGGAGAAATGCAGATGAACGCTAAACGCGTGGTTTATCCGATCCACCGGATCTAGATAAGATATCCGATCCACCGGATCTAGATAACTGATCATAATCAGCCATACCACATTTGTAGAGGTTTTACTTGCTTTAAAAAACCTCCCACACCTCCCCCTGAACCTGAAACATAAAATGAATGCAATTGGCGGCCGCCTCGAGCTGTGCCTTCTAGTTGCCAGCCATCTGTTGTTTGCCCCTCCCCCGTGCCTTCCTTGACCCTGGAAGGTGCCACTCCCACTGTCCTTTCCTAATAAAATGAGGAAATTGCATCGCATTGTCTGAGTAGGTGTCATTCTATTCTGGGGGGTGGGGTGGGGCAGGACAGCAAGGGGGAGGATTGGGAAGACAATAGCAGGCATGCTGGGGATGCGGTGGGCTCTATGG

## Local glucocorticoids and antibiotics treatment

For the treated eye, each participant was given tobramycin eye drops (5 ml; Tobrex, Alcon), pranoprofen eye drops (5 ml, 5 mg; Pranopulin, Senju), and prednisolone acetate eye drops (5 ml, 50 mg; Pred Forte, Allergan) (1 drop, qid, D3-D7) to prevent inflammation and infection. At the same time, each participant used atropine sulfate eye gel (2.5 g; Dishan, Xingqi; 0.2 g, bid, D3-D7) and tropicamide phenylephrine eye drops (10 ml; Meiduoli, Santen; 1 drop, bid, D3-D7) to prevent pupil adhesion. At the follow-up on D7, the researchers evaluated the status of the treated eyes to determine the following medication.

## ELISA to determine the humoral response to CYP4V2 protein

CYP4V2 antigen (Atlas APrEST72413) was diluted with phosphate-buffered saline (PBS) to a concentration of 2.8 μg/ml. Enhanced protein-binding ELISA plates (Costar #3590, Corning, NY, USA) were coated overnight at 4℃ with 50 μl of the diluted antigen. The plates were then washed (0.05% Tween-20 in calcium and magnesium-free PBS), blocked with 1% 1× ELISA Dilute (Invitrogen, 00-4202-56), and incubated with the test serum diluted 1:100 as a negative control or the sample diluent. CYP4V2 antibody diluted to 1:300, 1:900, 1:2700 with sample diluent was used as a positive control. After 1 hour of incubation at room temperature, the plates were washed and incubated with 100 μl of a 1:40,000 dilution of horseradish peroxidase-conjugated anti-human IgG (ABClonal) and a 1:1,0000 dilution of horseradish peroxidase-conjugated anti-rabbit antibody (CST) for 2 hours at room temperature. After washing, the color was developed using TMB substrate (Beyotime), followed by quenching with 25% sulfuric acid. Absorbance was read to obtain the optical density value at 450 nm (OD_450nm_) and the signal-to-noise ratio (S/N) was calculated as the mean OD_450nm_ value of the sample/mean OD_450nm_ value of the negative control. For ELISA, the mean OD difference between sample and control antigens is at least twice the background value. A positive result was defined as S/N > 2 when the sample was diluted 1:100.

## Introduction of AAV8-Luciferase used in the AAV8 neutralizing antibody assay

We constructed an AAV8-Luciferase vector harboring the same capsid protein as the study agent that is capable of autonomously expressing luciferase. HEK293A cells were infected with the AAV8-Luc construct, and the infection was evaluated by chemiluminescence detection using Bright-Lumi substrate and the luciferase reaction. AAV8-Luc was pre-incubated with a gradient dilution of the participant’s serum before infecting the cells. The anti-AAV8 neutralizing antibody in the serum blocked the AAV8-Luc infection of the cells, resulting in an attenuated fluorescence signal. Therefore, a decreased luciferase signal reflects the level of AAV8 neutralizing antibody.

# Discussion

All 12 participants showed significant variations in the immunogenicity test, except for the similar humoral immunity against CYP4V2. This phenomenon has also been observed in clinical trials of gene therapy for Duchenne’s muscular dystrophy and Leber’s congenital amaurosis.^1, 2^ However, the specific cause of these variations remains unclear. Since all participants received the same dosage of the virus, it is speculated that factors such as surgical procedures, the degree of blood-retinal barrier damage, and differences in Toll-like receptor 9 (TLR9) expression levels in the retina may contribute to this discrepancy. Notably, 2 participants with macular holes (R004 and R008) tested positive in multiple immunogenicity tests, which suggests that the virus may have entered the vitreous cavity through the hole. The breakdown of the blood-retinal barrier could potentiate pro-inflammatory peripheral immune activation against AAV and cause infiltration of the peripheral immune cells into the ocular tissue. Furthermore, TLR9 reacts to viral DNA with an unmethylated CpG motif, leading to the up-regulation of inflammatory cytokines and stronger adaptive immunity.^3-5^ However, accurately assessing the degree of blood-retinal barrier damage and TLR9 expression levels proved to be challenging, indicating the need for further research.

# Supplementary figures and tables


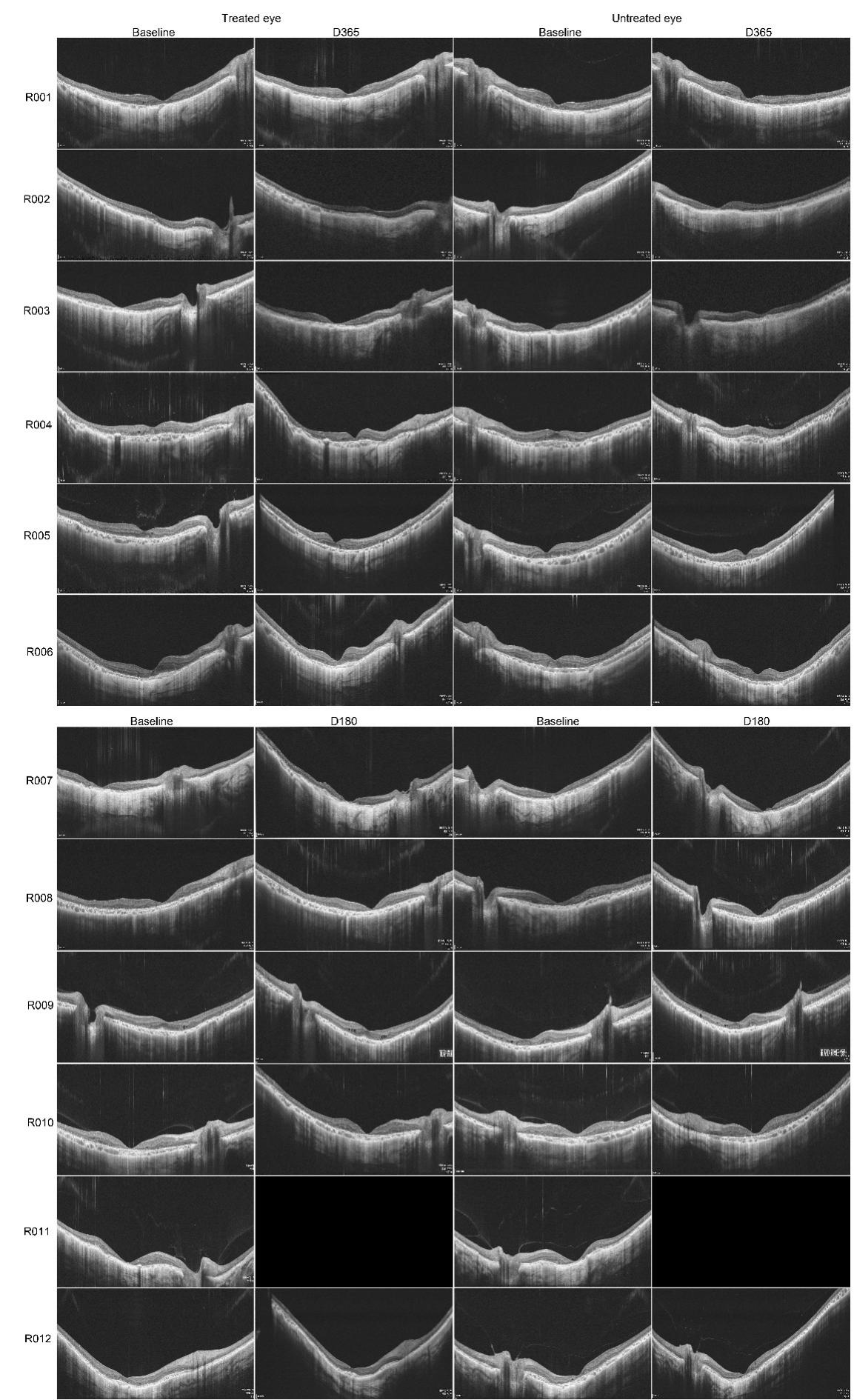


Figure S1. SS-OCT images of the 12 enrolled participants.

SS-OCT images of R001-R006 at baseline and D365, and R007-R012 at baseline and D180. Thinning of the fovea was observed in R004, which may be related to the postoperative macular hole. No obvious morphological changes were observed in the remaining participants. R011 did not visit the hospital on D180 owing to poor compliance. SS-OCT, swept-source optical coherence tomography; D, day.


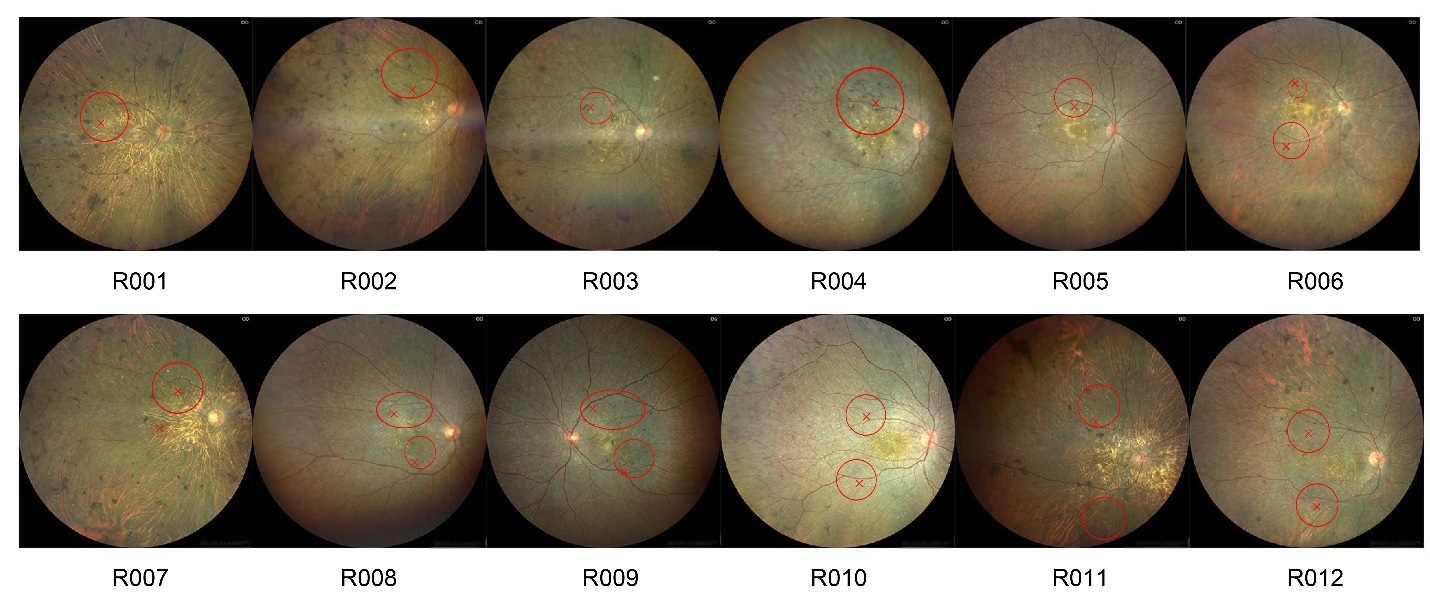


Figure S2. Retinotomies and subretinal blebs in the treated eyes of the 12 enrolled participants.

Retinotomies (red crosses) are located near the temporal vascular arcades. Subretinal blebs (red circles) extend to the macula or laterally to the vascular arcades. Despite injecting the same volume of viral particles, variations in bleb size were observed among the participants, probably due to individual differences in the retinal structure.


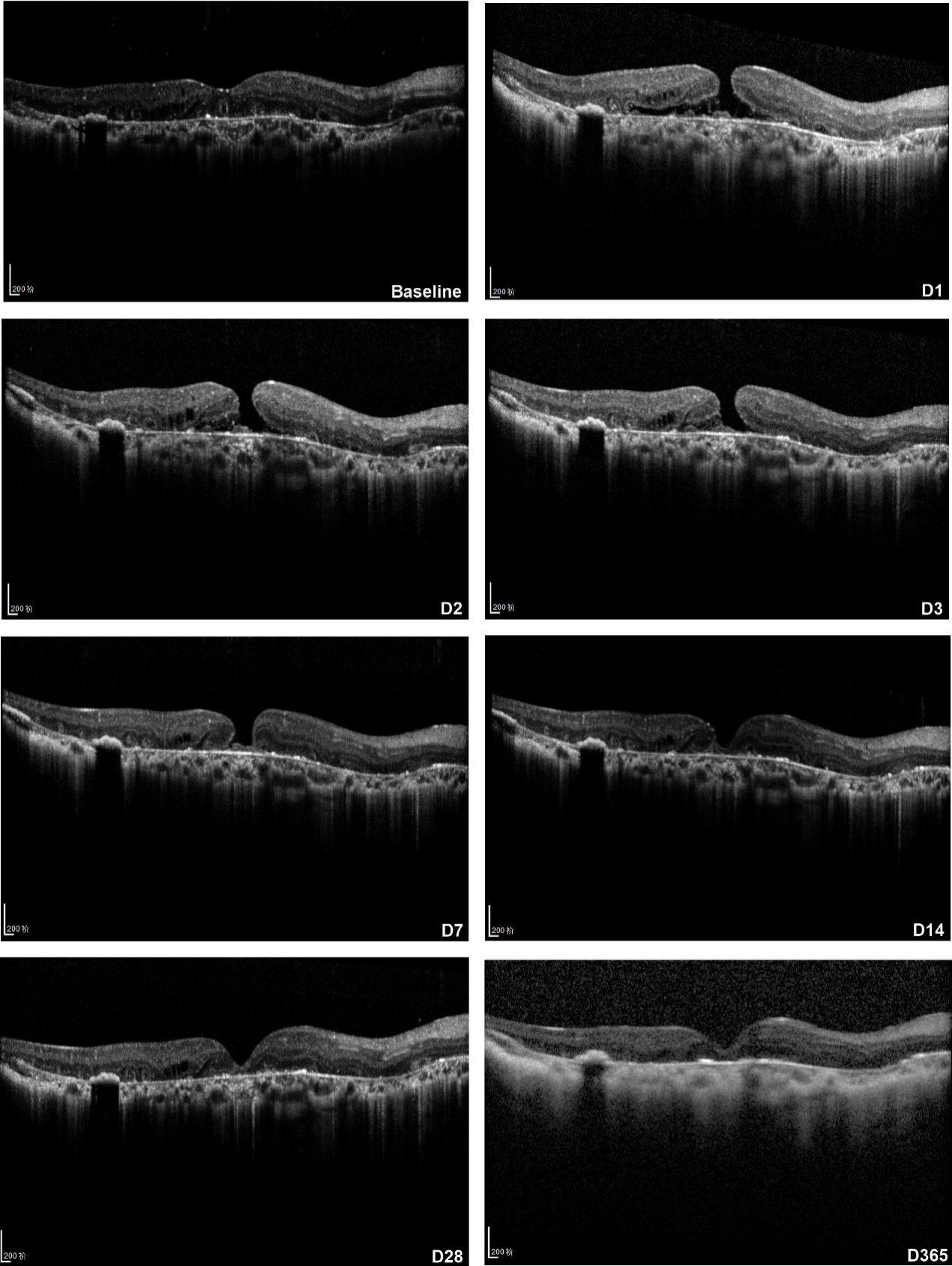


Figure S3. SD-OCT images through the fovea of R004.

A full-thickness macular hole was found in R004 on D1 after surgery, which healed on D14 without treatment. SD-OCT, spectral-domain optical coherence tomography; D: day.


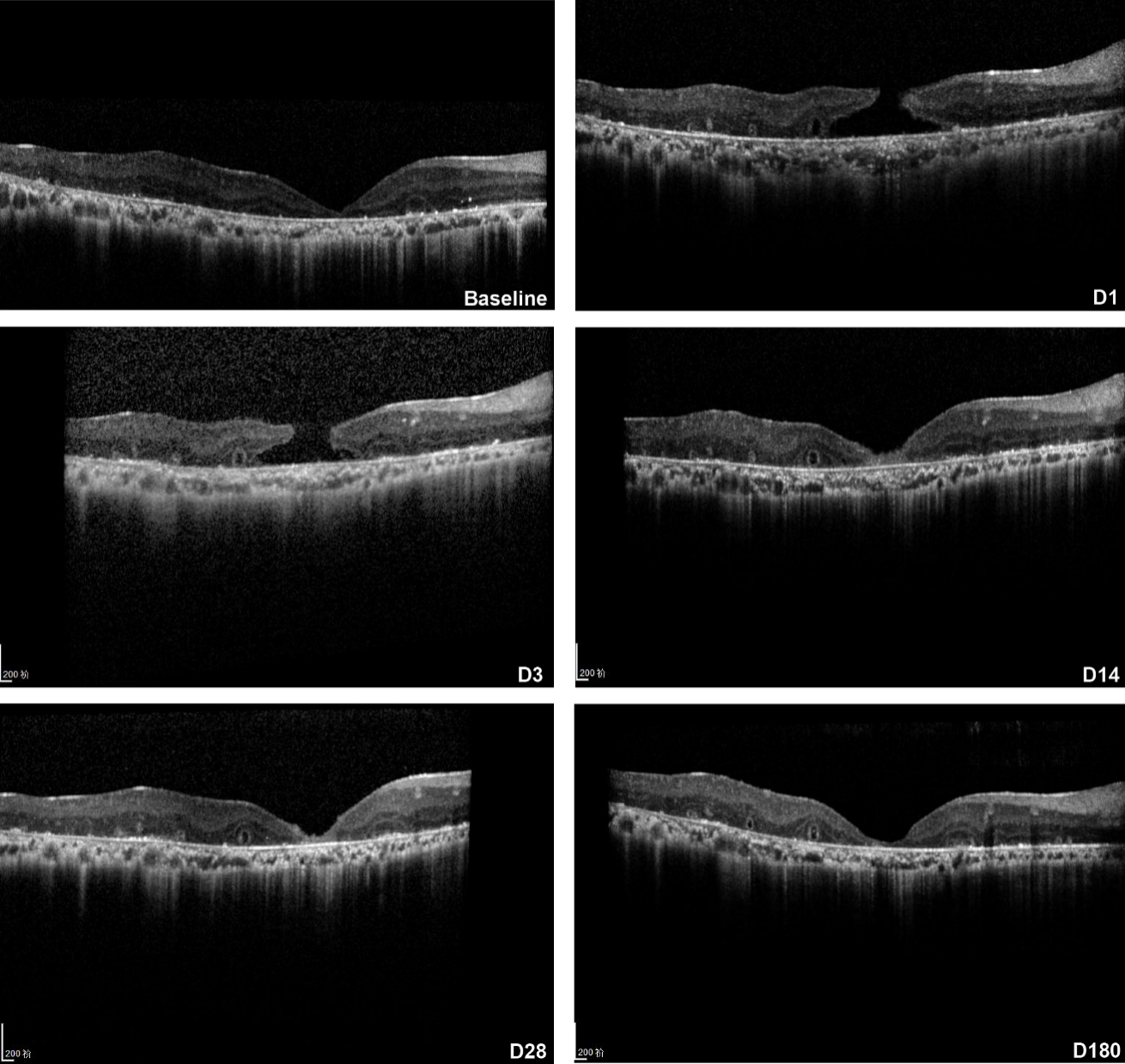


Figure S4. SD-OCT images through the fovea of R008.

A full-thickness macular hole was found in R008 on D1 after surgery, which healed on D14 without treatment. SD-OCT, spectral-domain optical coherence tomography; D: day.


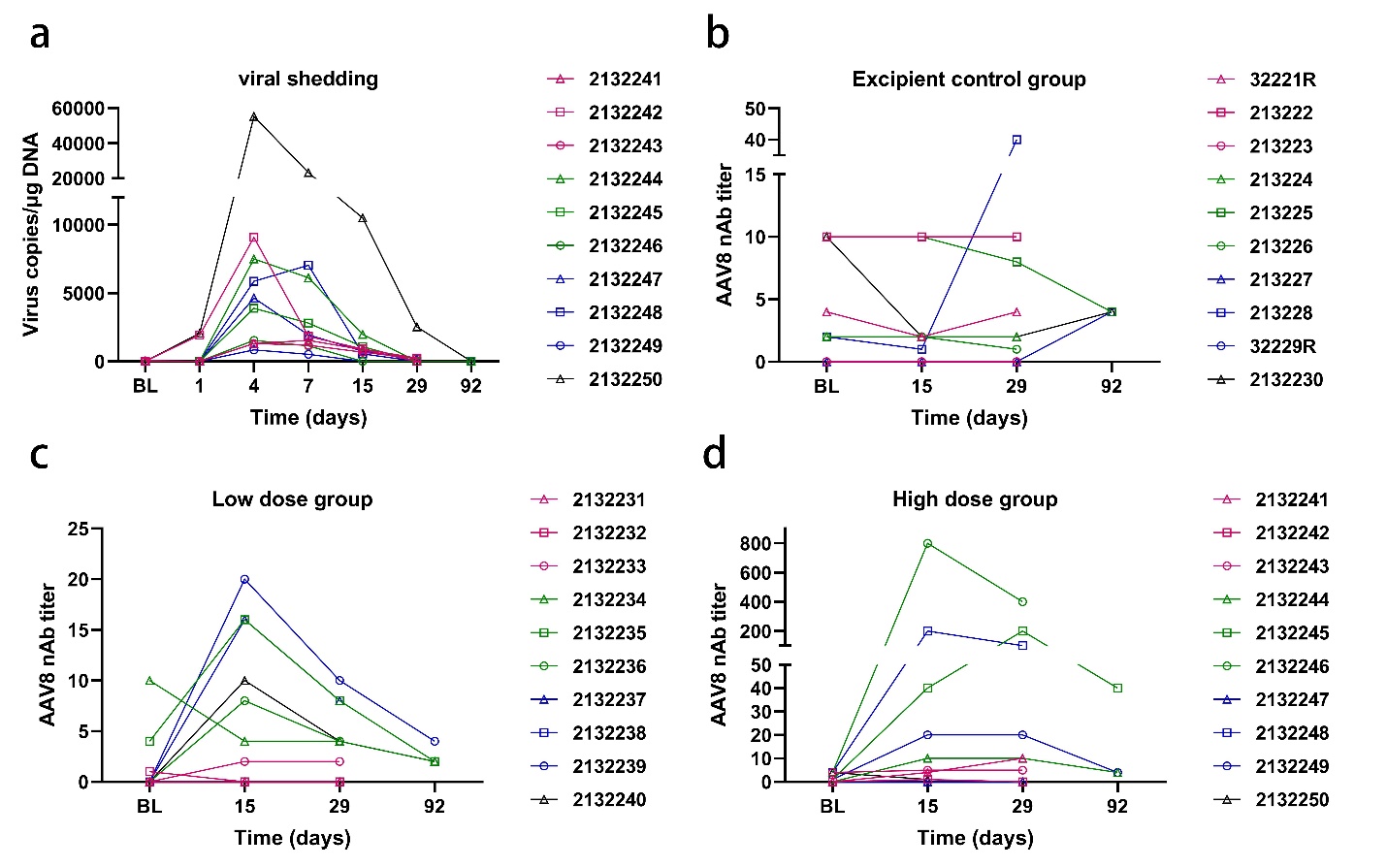


Figure S5. Vector shedding and neutralizing antibodies to AAV8 in cynomolgus monkey blood.

(a) Viral shedding in the blood of the high-dose group of cynomolgus monkeys. The lower limit of quantification was 5 copies/µg. (b) Neutralizing antibodies against AAV8 in the blood of cynomolgus monkeys in the excipient control group. (c) Neutralizing antibodies against AAV8 in the blood of cynomolgus monkeys in the low-dose group. (d) Neutralizing antibodies against AAV8 in the blood of cynomolgus monkeys in the high-dose group. Each monkey was dosed in both eyes and divided into 3 groups of 10 monkeys each, in which the low-dose group was dosed at 2.2 × 10^10^ vg/eye and the high-dose group was dosed at 2.9 × 10^11^ vg/eye. Each line represents one monkey, with the identification number for each monkey presented on the right side of each panel. In (b), (c), and (d), the AAV8 neutralizing antibody (nAb) titer on the *y*-axis represents the dilution at which the fluorescence inhibition is greater than 50% (e.g., a vertical coordinate equal to 10 represents a sample dilution ratio of 1:10).

.
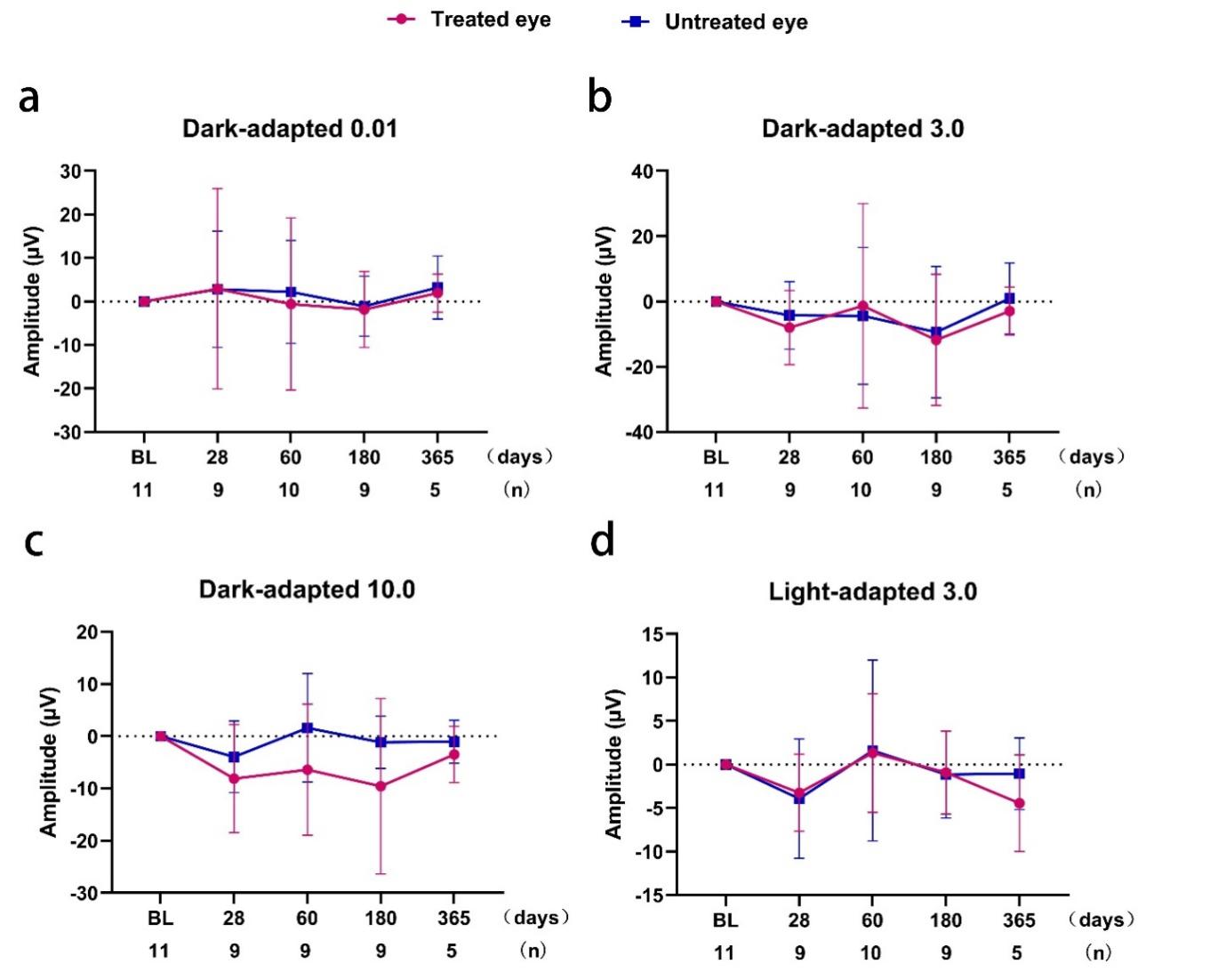


Figure S6. Changes in the b-wave amplitude of the 11 participants.

Dark-adapted b-wave amplitude at a flash strength of 0.01 cd.s/m^2^ (a), 3.0 cd.s/m^2^ (b), and 10 cd.s/m^2^ (c). (d) Light-adapted b-wave amplitude at a flash strength of 3.0 cd.s/m^2^. The number of treated eyes at each visit (n) is shown on the *x*-axis. ERG data during the cataract period (D1-D180) were not included for R005. ERG, electroretinogram; BL, baseline.


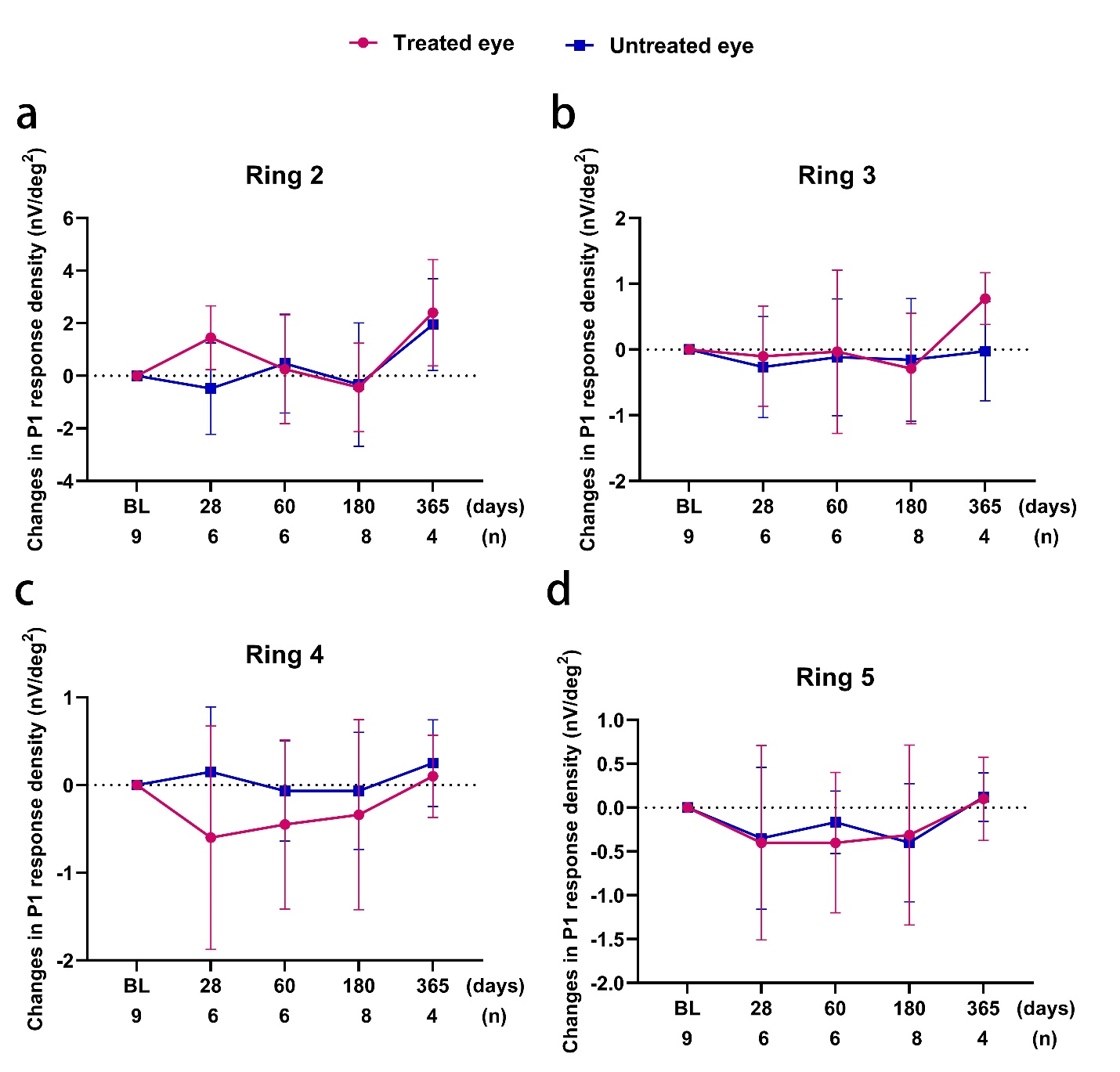


Figure S7. Changes in the P1-wave response density of rings 2-5 in mfERG.

Changes from baseline in the P1-wave response density of ring 2 (a), ring 3 (b), ring 4 (c), and ring 5 (d) in mfERG. This analysis did not include data for R003 and R011 because there was a lack of baseline measurements for R003 and follow-up for R011. The mfERG data during the cataract period (D1-D180) were not included for R005. At each visit, the number of participants with mfERG values (n) is shown on the *x*-axis. mfERG, multifocal electroretinogram; BL, baseline.


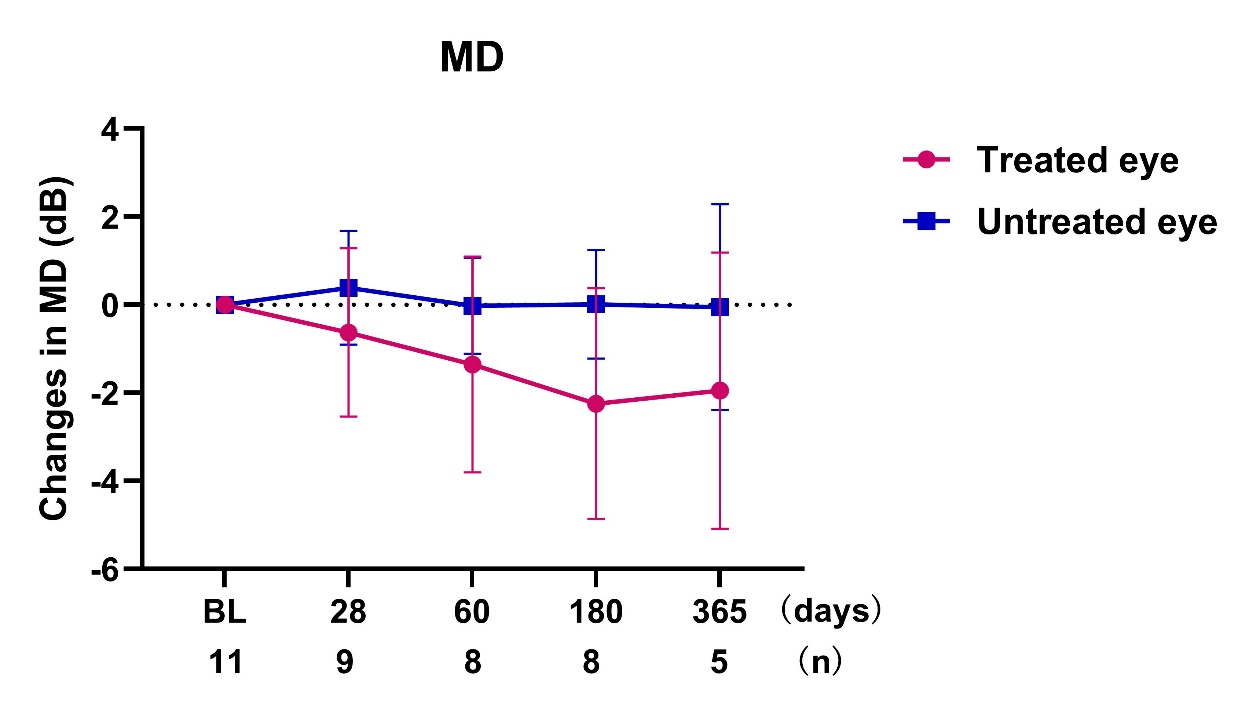


Figure S8. Changes from baseline in Humphrey visual fields.

Changes from baseline in mean deviation (MD) measured by Humphrey’s visual field before and after treatment. The number of subjects and MD or retinal sensitivity values at each visit (n) are shown below the *x*-axis. The visual field data during the cataract period (D1-D180) were not included for R005. This analysis did not include data for R011, owing to a lack of follow-up.


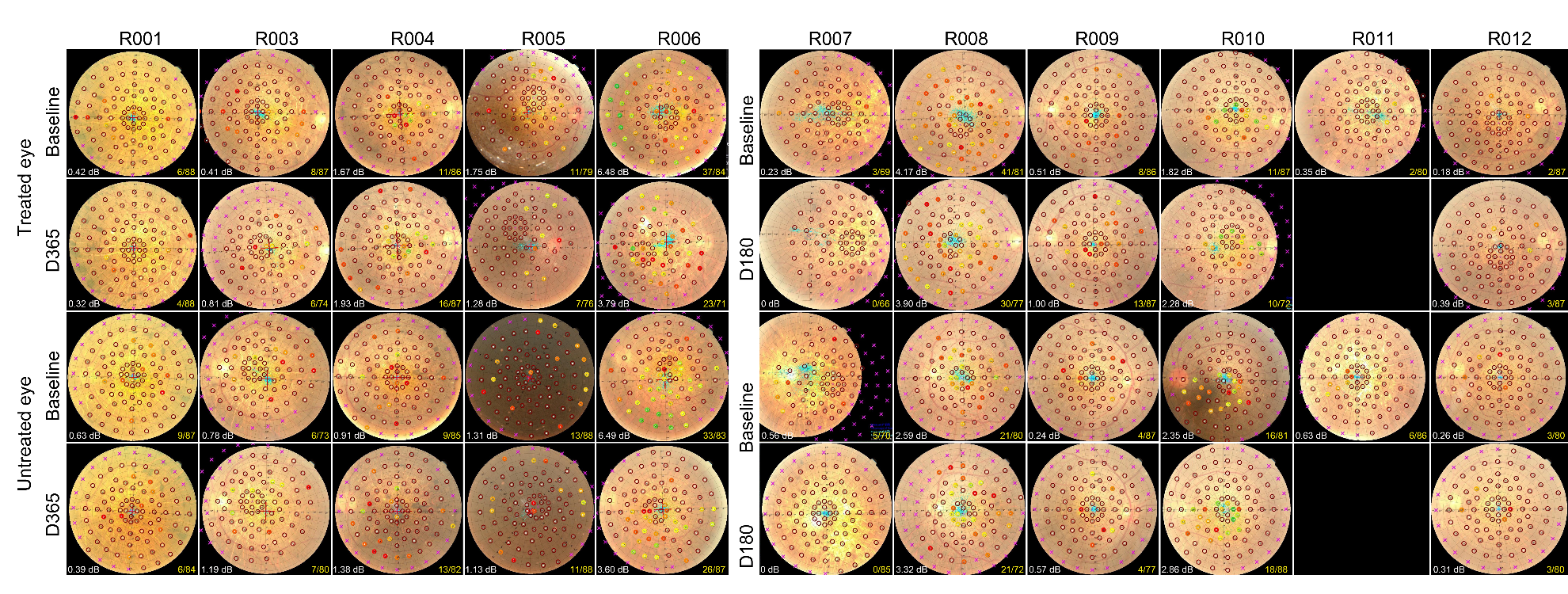


Figure S9. Participants’ retinal sensitivity and responding point numbers (40°).

The average retinal sensitivity of all detected points is noted in white typeset in each image (lower left corner) and the number of responding points divided by the number of all detected points is presented in yellow typeset (lower-right corner). Some of the loci in the outer rings could not be detected due to eyelash obstruction, unstable fixation, or other reasons. Retinal sensitivity at each visit is compared to the first visit shown directly above. The D28 visits of R005, D28 and D60 visits of R006, and D60 visits of R007 were all conducted at a local hospital rather than at the investigation site. The D28 visits of R008, D60 and D180 visits of R011, and the D60 visit of R012 are missing because of coronavirus disease 2019 pandemic or poor compliance.


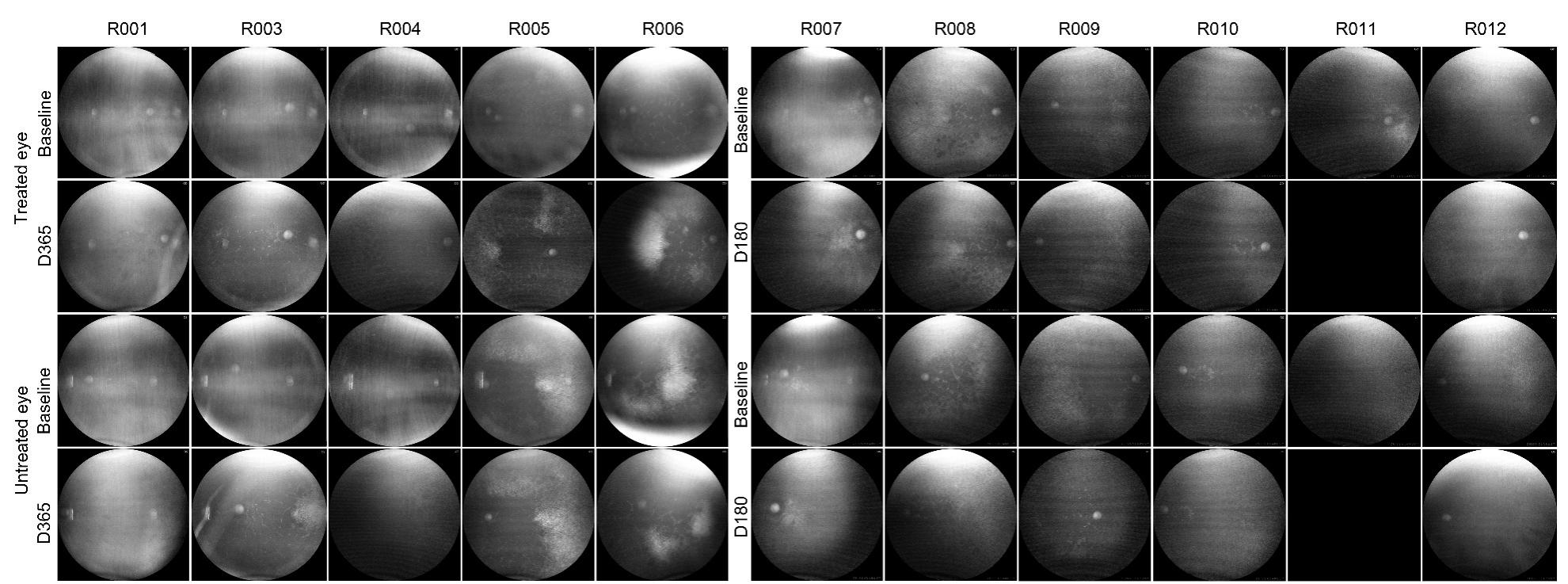


Figure S10. Participants’ FAF images.

FAF images of R001-R006 (baseline and D365) and R007-R012 (baseline and D180). R011 did not visit the hospital on D180 due to poor compliance. FAF, fundus autofluorescence; D, day.

Table S1. Biodistribution of the vector in the tears and blood (vector shedding).

The detection of vectors in blood samples presents certain challenges. The initial test results showed that vectors were detected in the blood samples of R002, R003 and R006 after 1 year of vector administration, prompting a re-test. However, all blood samples of the remaining subjects were lost, except for R006’s sample. The re-test of R006’s blood sample was negative, suggesting that the initial test results may not be reliable.

|  | Visit | Eye | R001 | R002 | R003 | R004 | R005 | R006 | R007 | R008 | R009 | R010 | R011 | R012 |  |
| --- | --- | --- | --- | --- | --- | --- | --- | --- | --- | --- | --- | --- | --- | --- | --- |
| Tear | Baseline | Treated | - | - | - | - | - | - | - | - | - | - | - | - |  |
|  |  | Untreated | - | - | - | - | - | - | - | - | - | - | - | - |  |
|  | 3 h | Treated | - | + | - | + | + | - | / | / | / | / | / | / |  |
|  |  | Untreated | - | - | - | - | - | - | / | / | / | / | / | / |  |
|  | D1 | Treated | - | - | - | + | - | - | - | + | - | - | - | - |  |
|  |  | Untreated | - | - | - | - | - | + | - | - | - | - | - | - |  |
|  | D2 | Treated | - | - | - | - | - | - | / | / | / | / | / | / |  |
|  |  | Untreated | - | - | - | + | - | - | / | / | / | / | / | / |  |
|  | D3 | Treated | - | - | - | - | - | - | / | / | / | / | / | / |  |
|  |  | Untreated | - | - | - | - | - | - | / | / | / | / | / | / |  |
|  | D7 | Treated | - | - | - | - | - | - | - | - | - | - | - | - |  |
|  |  | Untreated | - | - | - | - | - | - | - | - | - | - | - | - |  |
|  | D14 | Treated | / | / | / | / | / | / | - | - | - | - | - | - |  |
|  |  | Untreated | / | / | / | / | / | / | - | - | - | - | - | - |  |
|  | D28 | Treated | / | / | / | / | / | / | - | - | - | - | - | - |  |
|  |  | Untreated | / | / | / | / | / | / | - | - | - | - | - | - |  |
|  | D365 | Treated | - | - | - | - | - | - |  |  |  |  |  |  |  |
|  |  | Untreated | - | - | - | - | - | - |  |  |  |  |  |  |  |
| Blood | Baseline | | - | - | - | - | - | - | - | - | - | - | - | - |  |
|  | 3 h | | - | - | - | + | - | - | / | / | / | / | / | / |  |
|  | D1 | | - | + | + | - | - | - | - | - | - | - | - | - |  |
|  | D7 | | - | + | + | - | - | - | - | - | - | - | - | - |  |
|  | D14 | | / | / | / | / | / | / | - | - | - | - | - | - |  |
|  | D28 | | / | / | / | / | / | / | - | NA | - | - | - | - |  |
|  | D365 | | - | + | + | - | - | - |  |  |  |  |  |  |  |
| NA: Due to the COVID-19 or other reasons, no samples were collected; /: According to the clinical trial protocol, no sample collection was required for this visit. | | | | | | | | | | | | | | | |

Table S2. Humoral immune responses to AAV8.

| Patient | R001 | R002 | R003 | R004 | R005 | R006 | R007 | R008 | R009 | R010 | R011 | R012 |
| --- | --- | --- | --- | --- | --- | --- | --- | --- | --- | --- | --- | --- |
| Baseline | 1:20-1:1000 | <1:20 | 1:20-1:1000 | 1:20-1:1000 | 1:20-1:1000 | 1:20-1:1000 | <1:20 | <1:20 | 1:20-1:1000 | 1:20-1:1000 | 1:20-1:1000 | 1:20-1:1000 |
| D14 | 1:20-1:1000 | <1:20 | 1:20-1:1000 | 1:20-1:1000 | 1:20-1:1000 | 1:20-1:1000 | 1:20-1:1000 | >1:10000 | NA | 1:20-1:1000 | >1:10000 | 1:1000-1:10000 |
| D28 | 1:20-1:1000 | <1:20 | 1:20-1:1000 | 1:20-1:1000 | <1:20 | NA | 1:20-1:1000 | 1:1000-1:10000 | NA | 1:1000-1:10000 | NA | 1:1000-1:10000 |
| D90 | 1:20-1:1000 | 1:20-1:1000 | NA | 1:20-1:1000 | NA | 1:20-1:1000 | 1:20-1:1000 | 1:1000-1:10000 | 1:20-1:1000 | NA | NA | NA |
| D180 | / | / | / | / | / | 1:20-1:1000 | 1:20-1:1000 | 1:1000-1:10000 | 1:20-1:1000 | 1:1000-1:10000 | NA | 1:1000-1:10000 |
| D365 | 1:20-1:1000 | 1:20-1:1000 | 1:20-1:1000 | 1:1000-1:10000 | 1:20-1:1000 | 1:20-1:1000 |  |  |  |  |  |  |

NA: Due to the COVID-19 pandemic or other reasons, no samples were collected.

/: According to the clinical trial protocol, no sample collection was required for this visit.

Table S3. ETDRS visual acuity changes in the treated and untreated eyes.

| Eye | Visit | R001 | R003 | R004 | R005 | R006 | R007 | R008 | R009 | R010 | R011 | R012 | Mean | SD | *p* |
| --- | --- | --- | --- | --- | --- | --- | --- | --- | --- | --- | --- | --- | --- | --- | --- |
| Treated eye | BL | 0 | 0 | 0 | 0 | 0 | 0 | 0 | 0 | 0 | 0 | 0 |  |  |  |
|  | D14 | 26 | 4 | -2 | -16 | 5 | 0 | -5 | NA | 2 | 17 | -14 | 3.7 | 11.8 | 0.400 |
|  | D28 | 24 | 8 | 2 | NA | NA | 0 | -4 | NA | 8 | NA | -5 | 4.7 | 9.9 | 0.248 |
|  | D60 | 22 | 13 | NA | -11 | NA | NA | 4 | 14 | 6 | NA | NA | 11.8 | 7.2 | 0.063 |
|  | D90 | 23 | 10 | 1 | NA | 20 | 0 | -3 | 7 | NA | 23 | 0 | 9.0 | 10.5 | 0.042 |
|  | D180 | 20 | 12 | -2 | -23 | 9 | 31 | 4 | 6 | 3 | NA | -2 | 9.0 | 10.8 | 0.021 |
|  | D270 | 20 | NA | -5 | 10 | 11 |  |  |  |  |  |  | 9.0 | 10.4 | 0.250 |
|  | D365 | 22 | 7 | -5 | 18 | 13 |  |  |  |  |  |  | 11.0 | 10.6 | 0.125 |
| Untreated eye | BL | 0 | 0 | 0 | 0 | 0 | 0 | 0 | 0 | 0 | 0 | 0 |  |  |  |
|  | D14 | 3 | -4 | 3 | 0 | 2 | 32 | 5 | NA | 3 | 10 | -3 | 5.1 | 10.2 | 0.120 |
|  | D28 | 6 | 0 | 1 | NA | NA | 33 | 0 | NA | 5 | NA | -5 | 5.7 | 12.6 | 0.176 |
|  | D60 | 0 | 4 | NA | -1 | NA | NA | 5 | -4 | -2 | NA | NA | 0.3 | 3.5 | 0.786 |
|  | D90 | 2 | -4 | 1 | NA | 5 | 33 | 5 | 3 |  | 5 | -6 | 4.9 | 11.3 | 0.212 |
|  | D180 | -4 | 0 | 4 | 1 | -12 | 35 | -1 | 4 | 0 | NA | -9 | 1.8 | 12.8 | 0.944 |
|  | D270 | -2 | NA | 0 | -1 | -12 |  |  |  |  |  |  | -3.8 | 5.6 | 0.109 |
|  | D365 | 1 | 8 | 1 | -2 | -7 |  |  |  |  |  |  | 0.2 | 5.4 | 0.892 |

NA: not available. The BCVA of the treated eye of R005 from D1 to D180 was excluded from the statistical analysis. This was due to the development of cataracts related to the surgery and the absence of cataract surgery within 270 days post-administration, which had a significant impact on the assessment of visual acuity.

Table S4. B-wave amplitudes of the 11 participants before and after treatment.

| Eyes | Items | Data Type | Time | R001 | R003 | R004 | R005 | R006 | R007 | R008 | R009 | R010 | R011 | R012 | M | SD | *p* |
| --- | --- | --- | --- | --- | --- | --- | --- | --- | --- | --- | --- | --- | --- | --- | --- | --- | --- |
| TE | DA 0.01 | Actual value | BL | 0 | 0 | 0 | 0 | 22.7 | 0 | 37.7 | 76.7 | 0 | 0 | 0 |  |  |  |
|  |  | Changes from BL | BL | 0 | 0 | 0 | 0 | 0 | 0 | 0 | 0 | 0 | 0 | 0 |  |  |  |
|  |  |  | D28 | 0 | 0 | 0 | 40 | -94 | 0 | 5.6 | NA | 0 | NA | 0 | -5.4 | 35.8 | 1.000 |
|  |  |  | D60 | 0 | 0 | 62 | 0 | 5.7 | 79 | 2.7 | -3.5 | 0 | NA | 0 | 18.2 | 32.7 | 0.138 |
|  |  |  | D180 | 0 | 0 | 0 | 0 | 3 | 0 | -7 | 0.9 | 0 | NA | 0 | -0.5 | 3.4 | 1.000 |
|  |  |  | D365 | 0 | 0 | 0 | 0 | -5.3 |  |  |  |  |  |  | -1.3 | 2.7 | 0.317 |
|  | DA 3.0 | Actual value | BL | 0 | 11.5 | 0 | 23.2 | 28.4 | 0 | 65.2 | 196.8 | 0 | 0 | 0 |  |  |  |
|  |  | Changes from BL | BL | 0 | 0 | 0 | 0 | 0 | 0 | 0 | 0 | 0 | 0 | 0 | 0 | 0 |  |
|  |  |  | D28 | 0 | -48 | 0 | -2.5 | -44 | 0 | -18.6 | NA | 0 | NA | 0 | -12.6 | 19.9 | 0.068 |
|  |  |  | D60 | 0 | -48 | 55 | 0.6 | -44 | 20.5 | 15.6 | -9.4 | 0 | NA | 0 | -1.2 | 33.8 | 0.866 |
|  |  |  | D180 | 0 | -48 | 0 | -10.1 | 4.7 | 0 | -27 | -0.3 | 0 | NA | 0 | -10 | 19.6 | 0.273 |
|  |  |  | D365 | 0 | -48 | 0 | 4.7 | 3.3 |  |  |  |  |  |  | -9.9 | 25.2 | 1.000 |
|  | DA 10 | Actual value | BL | 0 | 11.6 | 0 | 20.7 | 25.7 | 0 | 64.9 | 180.4 | 0 | 0 | 0 |  |  |  |
|  |  | Changes from BL | BL | 0 | 0 | 0 | 0 | 0 | 0 | 0 | 0 | 0 | 0 | 0 | 0 | 0 |  |
|  |  |  | D28 | 0 | -50 | 0 | -53.7 | -45 | 0 | -5.6 | NA | 0 | NA | 0 | -17.1 | 24.5 | 0.068 |
|  |  |  | D60 | 0 | -50 | 0 | -9.1 | -45 | 0 | 2.6 | -10 | 0 | NA | 0 | -13.9 | 21.2 | 0.080 |
|  |  |  | D180 | 0 | -50 | 0 | -11.3 | 7.6 | 0 | -17.9 | -1.2 | 0 | NA | 0 | -8.8 | 19.7 | 0.273 |
|  |  |  | D365 | 0 | -50 | 0 | -5.3 | 0.3 |  |  |  |  |  |  | -13.7 | 24.3 | 0.285 |
|  | LA 3.0 | Actual value | BL | 0 | 12 | 0 | 22.5 | 8.1 | 0 | 19.6 | 35.9 | 0 | 0 | 0 |  |  |  |
|  |  | Changes from BL | BL | 0 | 0 | 0 | 0 | 0 | 0 | 0 | 0 | 0 | 0 | 0 | 0 | 0 |  |
|  |  |  | D28 | 0 | -40 | 0 | 0.1 | -40 | 0 | -38.7 | NA | 0 | NA | 0 | -13.2 | 19.9 | 0.141 |
|  |  |  | D60 | 0 | -40 | 32 | 1.7 | -3.6 | 21.7 | 3.6 | -1.8 | 0 | NA | 0 | 1.7 | 21.1 | 0.800 |
|  |  |  | D180 | 0 | -40 | 0 | -12.4 | -1.7 | 0 | -35.8 | 0.3 | 0 | NA | 0 | -11.1 | 18.5 | 0.144 |
|  |  |  | D365 | 0 | -40 | 0 | 3.2 | -3.5 |  |  |  |  |  |  | -10.1 | 20.2 | 0.285 |
| UE | DA 01 | Actual value | BL | 0 | 0 | 0 | 0 | 5.1 | 0 | 28.4 | 75.1 | 0 | 0 | 0 |  |  |  |
|  |  | Changes from BL | BL | 0 | 0 | 0 | 0 | 0 | 0 | 0 | 0 | 0 | 0 | 0 | 0 | 0 |  |
|  |  |  | D28 | 0 | 0 | 0 | 40 | -95 | 0 | 7.7 | NA | 0 | NA | 0 | -5.2 | 36.1 | 1.000 |
|  |  |  | D60 | 0 | 0 | 40 | 0 | -14 | 32.6 | 1.2 | -0.3 | 0 | NA | 0 | 7.4 | 18.6 | 0.345 |
|  |  |  | D180 | 0 | 0 | 0 | 0 | -0.3 | 0 | -6.7 | 3.5 | 0 | NA | 0 | -0.4 | 2.8 | 0.593 |
|  |  |  | D365 | 0 | 0 | 0 | 0 | -5.3 |  |  |  |  |  |  | -1.3 | 2.7 | 0.317 |
|  | DA 3.0 | Actual value | BL | 0 | 22.1 | 0 | 26.4 | 22.4 | 0 | 59.5 | 200.1 | 0 | 0 | 0 |  |  |  |
|  |  | Changes from BL | BL | 0 | 0 | 0 | 0 | 0 | 0 | 0 | 0 | 0 | 0 | 0 | 0 | 0 |  |
|  |  |  | D28 | 0 | -50 | 0 | -5.5 | -47 | 0 | -10.8 | NA | 0 | NA | 0 | -14.1 | 21.4 | 0.068 |
|  |  |  | D60 | 0 | -50 | 34 | -2.9 | -47 | 20.5 | 17.6 | -7.3 | 0 | NA | 0 | -4.3 | 30.3 | 0.735 |
|  |  |  | D180 | 0 | 7.3 | 0 | 0.9 | 3.2 | 0 | -2.8 | 0.3 | 0 | NA | 0 | 1.1 | 3.0 | 0.225 |
|  |  |  | D365 | 0 | 2.9 | 0 | 1.5 | 0.6 |  |  |  |  |  |  | 1.3 | 1.3 | 0.109 |
|  | DA 10 | Actual value | BL | 0 | 30.4 | 0 | 32.5 | 15.3 | 0 | 65.8 | 200.1 | 0 | 0 | 0 |  |  |  |
|  |  | Changes from BL | BL | 0 | 0 | 0 | 0 | 0 | 0 | 0 | 0 | 0 | 0 | 0 | 0 | 0 |  |
|  |  |  | D28 | 0 | -49 | 0 | -52.5 | -45 | 0 | 1.9 | NA | 0 | NA | 0 | -18.1 | 25.6 | 0.144 |
|  |  |  | D60 | 0 | -49 | 0 | -6.4 | -45 | 0 | 4.4 | -7.6 | 0 | NA | NA | -14.8 | 22.5 | 0.080 |
|  |  |  | D180 | 0 | -0.6 | 0 | 2.1 | 8.5 | 0 | -3 | -1.2 | 0 | NA | 0 | 0.7 | 3.4 | 0.893 |
|  |  |  | D365 | 0 | -1.7 | 0 | -2 | 3.8 |  |  |  |  |  |  | 0 | 2.7 | 1.000 |
|  | LA 3.0 | Actual value | BL | 0 | 18.8 | 0 | 21.1 | 6.1 | 0 | 15.4 | 39.2 | 0 | 0 | 0 |  |  |  |
|  |  | Changes from BL | BL | 0 | 0 | 0 | 0 | 0 | 0 | 0 | 0 | 0 | 0 | 0 | 0 | 0 |  |
|  |  |  | D28 | 0 | -43 | 0 | -3.3 | -39 | 0 | -39.6 | NA | 0 | NA | 0 | -15.6 | 20.7 | 0.068 |
|  |  |  | D60 | 0 | -43 | 35 | 0.9 | -2.4 | 21.7 | -2.6 | -2.7 | 0 | NA | 0 | 0.9 | 22.5 | 0.735 |
|  |  |  | D180 | 0 | -2.1 | 0 | 4.4 | -0.6 | 0 | -37.9 | -0.6 | 0 | NA | 0 | -4.6 | 13.6 | 0.343 |
|  |  |  | D365 | 0 | -5.9 | 0 | 5 | 0.6 |  |  |  |  |  |  | -0.1 | 4.5 | 1.000 |

BL: baseline; DA: dark-adapted; LA: light-adapted; M: mean; NA: not available. TE: treated eyes; UE: untreated eyes. The ERG of the treated eye of R005 from D1 to D180 was excluded from the statistical analysis owing to the development of cataracts related to the surgery and the absence of cataract surgery within 270 days post administration, which had a significant impact on the assessment of visual function.

Table S5. Change from baseline in VFQ-25 score at 180 days for 5 participants.

| Scale | R007 | R008 | R009 | R010 | R012 | Mean | SD | *p*-value |
| --- | --- | --- | --- | --- | --- | --- | --- | --- |
| Composite scores | 0.2 | 1.8 | 16.7 | 12.3 | -2.5 | 5.7 | 8.3 | 0.313 |
| General health | -25.0 | -25.0 | 75.0 | 25.0 | -50.0 | 0 | 50.0 | 0.891 |
| General vision | 20.0 | 0 | 20.0 | 20.0 | 20.0 | 16.0 | 8.9 | 0.046 |
| Ocular pain | -12.5 | 0 | 37.5 | 37.5 | 12.5 | 15.0 | 22.4 | 0.194 |
| Near activities | 16.7 | 16.7 | 33.3 | 16.7 | -8.3 | 15.0 | 14.9 | 0.074 |
| Distance activities | 4.2 | -8.3 | 41.7 | 8.3 | 0.0 | 9.2 | 19.2 | 0.357 |
| Social function | -50.0 | 25.0 | 12.5 | 25.0 | 0.0 | 2.5 | 31.1 | 0.713 |
| Mental health | 25.0 | 12.5 | 12.5 | -12.5 | 0.0 | 7.5 | 14.3 | 0.257 |
| Role limitations | 12.5 | -25.0 | 25.0 | 50.0 | 0.0 | 12.5 | 28.0 | 0.355 |
| Dependency | -41.7 | 16.7 | 16.7 | -16.7 | -33.3 | -11.7 | 27.4 | 0.336 |
| Driving | NA | NA | NA | NA | NA | NA | NA | NA |
| Color vision | NA | 0 | -50.0 | 25.0 | 0.0 | -6.3 | 31.5 | 0.655 |
| Peripheral vision | 25.0 | 25.0 | 0 | 25.0 | 25.0 | 20.0 | 11.2 | 0.046 |

NA: not applicable

R011 completed the questionnaire at baseline but was not followed up in hospital at the time of the D180 visit. Therefore, this table does not include data for R011.

Driving: R007 had hand motion vision at baseline but drove occasionally and was instructed by the investigator to stop driving, so he no longer drove postoperatively, therefore no driving score change could be calculated. R008 and R009 did not drive before surgery and occasionally drove after surgery and could not calculate a change in score. R010 and R011 never drove and therefore did not have this score.

R007 had a color vision question answer of 6 at baseline recorded as missing and therefore score change could not be calculated.

Table S6. Retinal light sensitivity of the central 20° of the retina before and after treatment in 11 participants.

| Eye | Data Type | Visit | R001 | R003 | R004 | R005 | R006 | R007 | R008 | R009 | R010 | R011 | R012 | Mean | SD | *p* |
| --- | --- | --- | --- | --- | --- | --- | --- | --- | --- | --- | --- | --- | --- | --- | --- | --- |
| Treated eyes | Actual  value | BL | 0.70 | 0.38 | 3.03 | 1.68 | 1.75 | 0.5 | 2.95 | 0.35 | 0.70 | 3.55 | 0.40 |  |  |  |
|  | Change from BL | BL | 0 | 0 | 0 | 0 | 0 | 0 | 0 | 0 | 0 | 0 | 0 |  |  |  |
|  |  | D30 | 0.25 | 0.13 | -0.20 | NA | NA | 08 | 0.58 | NA | 0.20 | NA | -05 | 0.14 | 0.25 | 0.150 |
|  |  | D60 | 0.10 | 0.28 | NA | -0.95 | NA | NA | 0.48 | 0 | 0.70 | NA | NA | 0.31 | 0.28 | 068 |
|  |  | D180 | -0.33 | 0.43 | 0.58 | -1.20 | 0.05 | -0.05 | 0.90 | 0.50 | 0.05 | NA | 0.25 | 0.26 | 0.37 | 065 |
|  |  | D365 | -0.28 | 0.23 | 0.23 | -0.75 | 1.10 |  |  |  |  |  |  | 0.11 | 0.69 | 0.892 |
| Untreated eyes | Actual value | BL | 0.90 | 1.18 | 0.85 | 0.15 | 6.10 | 0.5 | 3.40 | 0.40 | 0.95 | 3.48 | 0.38 |  |  |  |
|  | Change from BL | BL | 0 | 0 | 0 | 0 | 0 | 0 | 0 | 0 | 0 | 0 | 0 | 0 | 0 |  |
|  |  | D30 | -0.85 | 0.15 | 0.40 | NA | NA | NA | 1.23 | NA | 1.08 | NA | -0.10 | 0.32 | 0.77 | 0.313 |
|  |  | D60 | -0.80 | 0.15 | NA | 0.20 | NA | NA | 0.38 | -08 | 1.88 | NA | NA | 0.29 | 0.88 | 0.438 |
|  |  | D180 | -0.23 | 0.55 | 0.55 | 0.35 | -2.28 | -0.05 | 0.93 | 0.23 | 1.40 | NA | 05 | 0.15 | 0.98 | 0.202 |
|  |  | D365 | -0.63 | 0.65 | 0.35 | 0.15 | -2.45 |  |  |  |  |  |  | -0.39 | 1.25 | 1.000 |

Unit: dB; BL: baseline; NA: not available. The microperimetry of the treated eye of R005 from D1 to D180 was excluded from the statistical analysis owing to the development of cataracts related to the surgery and the absence of cataract surgery within 270 days post-administration, which had a significant impact on the assessment of visual function.

Table S7. Serum cholesterol and triglycerides levels of the 12 enrolled participants.

| Indicator | Visit | R001 | R002 | R003 | R004 | R005 | R006 | R007 | R008 | R009 | R010 | R011 | R012 |
| --- | --- | --- | --- | --- | --- | --- | --- | --- | --- | --- | --- | --- | --- |
| Cholesterol (mmol/L) | BL | 4.15 | 4.36 | 4.99 | **6.09** | 4.3 | **6.58** | 4.28 | **5.74** | 4.75 | 4.85 | 4.81 | 3.77 |
|  | D1 | 4.56 | 5.07 | 4.65 | **5.86** | 4.34 | **5.57** | 5.15 | **6.51** | 4.99 | **5.4** | **5.93** | 5.01 |
|  | D7 | **5.83** | **5.59** | 4.83 | **6.63** | 4.34 | **6.71** | 5.13 | **7.31** | **6.01** | 4.76 | **5.22** | **5.45** |
|  | D14 | **5.97** | 4.72 | 4.77 | **5.93** | 4.35 | **7.31** | **5.47** | **6.27** | **6.52** | **5.5** | **5.53** | **5.67** |
|  | D28 | **5.35** | 4.65 | **5.28** | **5.82** | 4.16 | NA | 4.58 | **5.71** | 5.10 | 4.87 | 4.4 | 4.67 |
|  | D60 | 4.2 | 4.26 | 4.83 | NA | 3.95 | NA | 4.04 | **5.25** | **5.24** | 4.56 | 5.05 | NA |
|  | D180 | 5.12 | 3.85 | **5.22** | **5.93** | 3.45 | **6.97** | 4.19 | **5.49** | 4.8 | 4.44 | NA | 4.65 |
|  | D365 | 5 | 4.2 | 5.21 | **5.84** | 4.06 | **6.51** |  |  |  |  |  |  |
| Triglyceride (mmol/L) | BL | 0.9 | 0.53 | 0.66 | 0.94 | 0.98 | 1.12 | 1.11 | 0.92 | 1.15 | 1.08 | 1.66 | 0.41 |
|  | D1 | **1.87** | 0.68 | 1.06 | **2.72** | 1.45 | 0.72 | 0.48 | 0.88 | 0.81 | 1.11 | 1.58 | 0.74 |
|  | D7 | **2.99** | 0.82 | 1.17 | 1.63 | 1.55 | 0.62 | 1.32 | 1.26 | 1.01 | **2.64** | 1.53 | 0.71 |
|  | D14 | **3.39** | 1.25 | 0.78 | 0.86 | 1.12 | **1.73** | **1.95** | 1.42 | **1.76** | **1.77** | **2.87** | 0.61 |
|  | D28 | **1.92** | 0.78 | 0.8 | 1.49 | 1.07 | NA | 0.79 | 1.12 | 1.38 | 1.23 | 1.20 | 0.47 |
|  | D60 | **2.59** | 1.38 | 0.63 | NA | 0.76 | NA | 0.53 | 1.15 | **2.01** | 1.14 | **1.84** | NA |
|  | D180 | **1.73** | 0.63 | 0.59 | 1.08 | 0.81 | 1.28 | 1.12 | 0.73 | **2.28** | 0.99 | NA | 0.62 |
|  | D365 | **2.17** | 0.62 | 0.59 | 1.54 | 1.04 | 0.89 |  |  |  |  |  |  |

The normal range for cholesterol is 2.1-5.17 mmol/L and that for triglycerides is 0.56-1.70 mmol/L.

NA: not available; Bold face: above normal.

# References

1. Mendell, J.R., et al., *Dystrophin Immunity in Duchenne's Muscular Dystrophy.* New England Journal of Medicine, 2010. **363**(15): 1429-1437.

2. Maguire, A.M., et al., *Age-dependent effects of RPE65 gene therapy for Leber's congenital amaurosis: a phase 1 dose-escalation trial.* Lancet, 2009. **374**(9701): 1597-605.

3. Blasius, A.L. and B. Beutler, *Intracellular toll-like receptors.* Immunity, 2010. **32**(3): 1097-4180

4. Rogers GL, S.M., Zolotukhin I, Markusic DM, Morel LM, Lee B, Ertl HC, Herzog RW. , *Unique Roles of TLR9- and MyD88-Dependent and -Independent Pathways in Adaptive Immune Responses to AAV-Mediated Gene Transfer.* J Innate Immun, 2015. **7**(3): 1662-8128

5. Mohan, K.V., et al., *Immunological consequences of compromised ocular immune privilege accelerate retinal degeneration in retinitis pigmentosa.* Orphanet Journal of Rare Diseases, 2022. **17**(1).

Data S1. T-cell immune responses to AAV8 and CYP4V2.

| Antigen | Visit | R001 | R002 | R003 | R004 | R005 | R006 | R007 | R008 | R009 | R010 | R011 | R012 |
| --- | --- | --- | --- | --- | --- | --- | --- | --- | --- | --- | --- | --- | --- |
| AAV8 | Baseline | 0.9 | 1.1 | 1.7 | 4.2 | 0.6 | 0.8 | 1.2 | 1.9 | 1.5 | 0.6 | 1.4 | 0.7 |
|  | D14 | 1.3 | 0.95 | 1.1 | 3.6 | NA | 1.1 |  | 1 | NA | 2.3 | 0 | 1.3 |
|  | D28 | 2 | 0.7 | 1.5 | 2.8 | 0.8 | NA | 1.3 | 1.5 | NA | 1 | NA | 0.7 |
|  | D90 | / | / | / | / | / | 1.2 | 0.8 | 1 | 4 | NA | NA |  |
|  | D180 | / | / | / | / | / |  | 5.4 | 4 | 0.3 | 4 | 1.2 | 0.5 |
|  | D365 | 0.9 | 4.5 | 0.4 | 1 | 0.6 | 1.8 | 0 | 4 |  |  |  |  |
| CYP4V2 | Baseline | 1.4 | 3.5 | 3.1 | 16.7 | 0.7 | 0.9 | 3.2 | 7.4 | 1 | 2.5 | 1.3 | 2.5 |
|  | D14 | 2.2 | 3.8 | 1.3 | 2.6 | NA | 3.6 |  | 1.3 | NA | 0.8 | 2 | 4.4 |
|  | D28 | 3.6 | 1.3 | 2.4 | 55.6 | 1.3 | NA | 2.1 | 4 | NA | 11 | NA | 1.2 |
|  | D90 | / | / | / | / | / | 13.9 | 2.5 | 6.3 | 6.1 | NA | NA |  |
|  | D180 | / | / | / | / | / |  | 4.8 | 1.8 | 1 | 2 | 1.1 | 0.5 |
|  | D365 | 1.7 | 4.6 | 0.4 | 2.8 | 2.4 | 2.13 | 0 | 6 |  |  |  |  |

NA: Due to the COVID-19 pandemic or other reasons, no samples were collected.

/: According to the clinical trial protocol, no sample collection was required for this visit.
