# Supplementary material for: Gene replacement therapy in Bietti crystalline corneoretinal dystrophy: an open-label, single-arm, exploratory trial
Source: Signal Transduct Target Ther. 2024 Apr 24;9:95. doi: 10.1038/s41392-024-01806-3 (PMC11039457; doi:10.1038/s41392-024-01806-3)
Supplement: Supplementary file 2 — Clinical Trial Protocol [file 41392_2024_1806_MOESM2_ESM.docx]

A Safety Study of a Single Subretinal Injection of rAAV2/8-hCYP4V2 Gene Replacement Therapy in Patients with Bietti's Crystalline Dystrophy (BCD)

Study Protocol

Protocol Number: ZVS101e-01

Version No.: V6.0

Version Date: November 01, 2021

Principal Investigator: Prof. Wenbin Wei, Director Xiuli Zhao

Sponsor: Beijing Tongren Hospital, Capital Medical University

Cooperative Unit: Chigenovo Co., Ltd.

| **Protocol Signature Page** |
| --- |
| **1. Principal Investigator** |
| I will conscientiously perform the responsibilities of the investigator.  I will be responsible for making clinically relevant medical decisions to ensure that subjects are promptly and properly treated in case of adverse events during the study, and I will record these events as required.  I guarantee that the data will be truly, accurately, completely, timely and legally recorded in the case report form to ensure the quality of the clinical study.  I will provide a curriculum vitae before the study initiation, which will be submitted to the Ethics Committee. |
|  |
| Sponsor: Beijing Tongren Hospital, Capital Medical University |
| Principal Investigator (Signature): |
| Date of Signature: |
| Contact Number: |

| **Protocol Signature Page** |
| --- |
| **2. Principal Investigator** |
| I will conscientiously perform the responsibilities of the investigator.  I will be responsible for making clinically relevant medical decisions to ensure that subjects are promptly and properly treated in case of adverse events during the study, and I will record these events as required.  I guarantee that the data will be truly, accurately, completely, timely and legally recorded in the case report form to ensure the quality of the clinical study.  I will provide a curriculum vitae before the study initiation, which will be submitted to the Ethics Committee. |
|  |
| Sponsor: Beijing Tongren Hospital, Capital Medical University |
| Principal Investigator (Signature): |
| Date of Signature: |
| Contact Number: |

| **Protocol Signature Page** |
| --- |
| **3. Cooperative Unit** |
| We will earnestly fulfill the relevant responsibilities of partner as specified in the protocol, be responsible for funding and monitoring this clinical study, and assisting the sponsor Beijing Tongren Hospital of Capital Medical University to carry out the clinical study according to the protocol. |
|  |
| Cooperative Unit: Chigenovo Co., Ltd. |
| Project Leader (Signature): |
| Date of Signature:  Contact Number: |
|  |

**Table of Contents**

[Protocol Synopsis 7](#_Toc159422927)

[Table 1. Study Flow Chart 12](#_Toc159422928)

[1. Study Background 15](#_Toc159422929)

[1.1 Disease Background and Treatment Status 15](#_Toc159422930)

[1.2 Investigational Drug Background 17](#_Toc159422931)

[2. Study Objectives 18](#_Toc159422932)

[3. Study Design 19](#_Toc159422933)

[3.1 Overall Design 19](#_Toc159422934)

[3.2 Rationale for Dose Setting 19](#_Toc159422935)

[3.3 Definition of End of Study 20](#_Toc159422936)

[4. Study Population 21](#_Toc159422937)

[4.1 Inclusion Criteria 21](#_Toc159422938)

[4.2 Exclusion Criteria 21](#_Toc159422939)

[4.3 Withdrawal Criteria 22](#_Toc159422940)

[5. Study Intervention 23](#_Toc159422941)

[5.1 Investigational Drug 23](#_Toc159422942)

[5.1.1 Investigational drug information 23](#_Toc159422943)

[5.1.2 Dosing regimen 23](#_Toc159422944)

[5.1.3 Packaging and labeling 23](#_Toc159422945)

[5.1.4 Drug storage and management 23](#_Toc159422946)

[5.2 Concomitant Therapies 24](#_Toc159422947)

[6. Study Flow and Study Procedure 25](#_Toc159422948)

[6.1 Study Flow 25](#_Toc159422949)

[6.1.1 Screening baseline visit 25](#_Toc159422950)

[(V1, 1 week pre-op) 25](#_Toc159422951)

[6.1.2 Observation period 26](#_Toc159422952)

[6.1.3 Follow-up period 31](#_Toc159422953)

[6.2 Study Procedure 32](#_Toc159422954)

[6.2.1 Sign informed consent form 32](#_Toc159422955)

[6.2.2 Collection of demographic data 33](#_Toc159422956)

[6.2.3 Collection of medical history and treatment history 33](#_Toc159422957)

[6.2.4 Verification of inclusion and exclusion criteria 33](#_Toc159422958)

[6.2.5 See Appendix 3 for the test items during the study. 33](#_Toc159422959)

[6.2.6 See Sample Management Manual for sample collection, processing and shipment procedures during the study in details. 33](#_Toc159422960)

[6.2.7 See Sample Management Manual for biological sample collection time windows in details. 33](#_Toc159422961)

[6.2.8 Documentation of concomitant medications 33](#_Toc159422962)

[7. Study Evaluation 34](#_Toc159422963)

[7.1 Safety Evaluation 34](#_Toc159422964)

[7.2 Preliminary Efficacy Exploratory Measures 34](#_Toc159422965)

[8. Adverse Events and Serious Adverse Events 35](#_Toc159422966)

[8.1 Adverse Events 35](#_Toc159422967)

[8.2 Serious Adverse Event 37](#_Toc159422968)

[9. Statistical Considerations 37](#_Toc159422969)

[9.1 Statistical Hypothesis and Sample Size Estimation 37](#_Toc159422970)

[9.1.1 Statistical hypothesis 37](#_Toc159422971)

[9.1.2 Sample size estimation 37](#_Toc159422972)

[9.2 Analysis Populations 37](#_Toc159422973)

[9.3 Statistical Analysis 38](#_Toc159422974)

[9.3.1 General methods 38](#_Toc159422975)

[9.3.2 Description of baseline conditions 38](#_Toc159422976)

[9.3.3 Safety 38](#_Toc159422977)

[9.3.4 Efficacy 38](#_Toc159422978)

[10. Data Management 39](#_Toc159422979)

[11. Study Quality Control and Assurance 39](#_Toc159422980)

[12. Ethical Principles 39](#_Toc159422981)

[13. References 40](#_Toc159422982)

# Abbreviations

| Abbreviation | Full names |
| --- | --- |
| rAAV | Recombinant adeno-associated virus |
| vg | Viral genome |
| AE | Adverse events |
| APTT | Activated partial thromboplastin time |
| BCVA | Best-corrected visual acuity |
| Cr | Creatinine |
| CNV | Choroidal neovascularisation |
| eCRF | Electronic case report form |
| ELISA | Enzyme-linked immunosorbent assay |
| ETDRS Chart | Early Treatment of Diabetic Retinopathy Study |
| FAS | Full Analysis Set |
| GCP | Good Clinical Practice |
| HbsAg | Hepatitis B surface antigen |
| HCV-Ab | Hepatitis C virus antibody |
| HIV-Ab | Human immunodeficiency virus antibody |
| IOP | Intraocular pressure |
| NMPA | National Medical Products Administration |
| PT | Prothrombin time |
| SAE | Serious adverse events |
| SS | Safety Set |
| TBIL | Total bilirubin |
| TC | Total cholesterol |
| TG | Triglycerides |
| OCT | Optical coherence tomography |

# Protocol Synopsis

| **Sponsor:** Beijing Tongren Hospital, Capital Medical University |
| --- |
| **Cooperative Unit:** Chigenovo Co., Ltd. |
| **Study Phase:** Investigator-initiated trial |
| **Study Title:** A Safety Study of a Single Subretinal Injection of rAAV2/8-hCYP4V2 Gene Replacement Therapy in Patients with Bietti's Crystalline Dystrophy (BCD) |
| **Indication: Bietti's crystalline dystrophy (BCD)** |
| **Study Objectives:**  Primary objective: To evaluate the safety of a single subretinal injection of rAAV2/8-hCYP4V2 in patients with Bietti's crystalline dystrophy (BCD).  Secondary objective: To preliminarily explore the clinical efficacy of rAAV2/8-hCYP4V2 gene replacement therapy. |
| **Investigational Drug:** rAAV2/8-hCYP4V2 Ophthalmic Injection, provided by Chigenovo Co., Ltd. and manufactured by OBiO Technology (Shanghai) Corp., Ltd. (The internal code is ZVS101e in Chigenovo Co., Ltd., and C035 Injection in OBiO Technology (Shanghai) Corp., Ltd.)  **Strength:** 2×10^12^ vg/mL, 0.2 mL/vial, diluted to 5×10^8^ vg/uL before use  **Injection Volume:** 150 uL  **Dose of Administration:** 7.5×10^10^ vg  **Method of administration:** Subretinal injection |
| **Rationale for Dose Design**  The investigational drug rAAV2/8-hCYP4V2 Ophthalmic Injection is a gene replacement therapy drug. In preliminary safety clinical study (ClinicalTrials No.: NCT04722107), enrollment, administration, and 3-month safety and efficacy observation have been completed in 3 subjects. During the observation period, no drug- or treatment-related serious adverse reactions occurred in any of the 3 subjects; meanwhile, 2 of the subjects showed significant improvements in visual acuity and Multi-Luminance Mobility Test (MLMT) score. It can be seen that the clinical dose of 5×10^8^ vg/uL has good safety and certain efficacy. Preclinical pharmacodynamic experiment has preliminarily demonstrated that 1 uL of the following concentrations of rAAV2/8-hCYP4V2 were injected into the subretinal space of CYP4V2 knockout mice: CYP4V2 protein was expressed in 1×10^8^, 5×10^8^, 1×10^9^, 3×10^9^, 6×10^9^ and 1×10^10^ (unit: vg/uL), and the 6-month in vivo observation at following three doses of 3×10^9^ vg, 6×10^9^ vg and 1×10^10^ vg showed that 3×10^9^ vg had a significant therapeutic effect without obvious toxicities, and 6×10^9^ vg and 1×10^10^ vg had efficacy in the early stage, but obvious retinal toxicity in the later stage.  Referring to Luxturna (produced by Spark, intended for LCA patients carrying RPE65 gene mutation), the only gene replacement therapy marketed in the field of inherited retinal degeneration, the dose escalation concentration of Luxturna is designed as: 1×10^8^ (150 uL), 3.2×10^8^ (150 uL) and 5×10^8^ (300 uL) (unit: vg/uL). The dose escalation study results showed that all of the above three concentrations had therapeutic effect without obvious toxicities. The recommended Phase 3 clinical trial concentration of Luxturna was 5×10^8^ vg/uL (300 uL), which was the maximum concentration tested in vivo.  Referring to the clinical study data of RPGR gene replacement therapy drug published in Nature Medicine in February 2020, the dose escalation concentrations were designed as six doses: 5×10^7^ (40-100 uL), 1×10^8^ (40-100 uL), 5×10^8^ (30-100 uL), 1×10^9^ (60-100 uL), 2.5×10^9^ (50-150 uL) and 5×10^9^ (30-80 uL) (unit: vg/uL). The results showed that 5×10^7^ vg/uL and 1×10^8^ vg/uL groups had no significant improvement in visual function, 5×10^8^ vg/uL group had significant improvement, and 7 of 9 subjects in the three high-dose groups (1×10^11^ vg, 2.5×10^11^ vg and 5×10^11^ vg) showed mild inflammatory reaction, which could be effectively managed after hormone therapy.  Based on the preclinical results of the investigational drug and comparison with similar drugs, and referring to the dose design for Phase 1 dose escalation study and recommended clinical dose of the similar marketed drug Luxturna, the concentration gradient selected in this project is: 1×10^8^ vg/uL, 5×10^8^ vg/uL, 1×10^9^ vg/uL and 3×10^9^ vg/uL, and the injection dose is 150 uL. In this preliminary experiment, the concentration selected was 5×10^8^ vg/uL, 150 uL, so the injection dose was 7.5×10^10^ vg. |
| **Study Design**  This is a single-center, open-label, single-arm and single-dose study and aimed to evaluate the safety and preliminary efficacy of the investigational drug in patient with BCD.  **Selection Criteria for Target Eye**  The target eye must meet the following requirements:  1) Best-corrected visual acuity is counting finger to 0.3 (equivalent to Snellen visual counting finger to 20/63); able to perform visual examination and retinal function test; photoreceptor layer, that is, the outer nuclear layer can be observed in macular area under standard optical coherence tomography (OCT);  2) The eye with poorer visual acuity in both eyes of the candidate subject is the target eye. Note: For all subjects, only one eye is designated as the "target eye" (i.e., treated eye). If both eyes of patients meet the inclusion criteria, the target eye will be medically determined by the investigator.  **Safety** **observation**  The investigational product rAAV2/8-hCYP4V2 Ophthalmic Injection is a gene replacement therapy product. In preliminary safety clinical study (ClinicalTrials No.: NCT04722107), enrollment, administration, and 3-month safety and efficacy observation have been completed in 3 subjects. During the observation period, no drug- or treatment-related serious adverse reactions occurred in any of the 3 subjects; meanwhile, 2 of the subjects showed significant improvements in visual acuity and MLMT score. It can be seen that the clinical dose of 5×10^8^ vg/uL has good safety and certain efficacy. In this clinical study, additional 9 subjects (12 subjects in total) are planned to be enrolled, and a total of 1 dose group, 7.5×10^10^ vg, is set. After one subject is enrolled first and no significant toxic and side effects are observed within 14 days after administration, the study in the second subject may be initiated. After the second subject completes administration and no significant toxic and side effects are observed within 14 days, the study in the third subject may be initiated; and so on. While conducting safety assessment, tear and blood samples are collected from subjects to detect DNA of rAAV2/8 virus and immune responses against the therapeutic vector.  **Preliminary Exploratory observation**  Clinical efficacy is preliminarily assessed by best-corrected visual acuity (BCVA, ETDRS chart), macular microperimetry, mfERG, color vision, dark-adapted contrast sensitivity, and MLMT. |
| Study Population  Inclusion Criteria  Subjects who meet all of the following inclusion criteria are considered eligible for participation in this study.  1) Age ≥ 18 years (inclusive) at the time of informed consent;  2) Patients with clinically confirmed Bietti's crystalline dystrophy (BCD);  3) Carrying two causative mutations in CYP4V2 gene confirmed by genetic testing;  4) Meet selection criteria for target eye;  5) The subject and his/her spouse agree to take effective contraceptive measures during the study and within 1 year after administration;  6) Voluntarily participate in this clinical study and have signed informed consent form.  Exclusion Criteria  Subjects meeting any of the following criteria will not be eligible to participate in this study.  1) The patient lacks sufficient viable retinal photoreceptor cells, has less than 1 disc area of retina photoreceptor cells or has a retinal thickness of less than 100 μm.  2) Active choroidal neovascularisation (CNV) lesion secondary to BCD in the target eye judged by the investigator; or other ocular disorders that would preclude surgery or interfere with interpretation of the study endpoints;  3) Medications within 3 months prior to screening that may affect study observation (e.g., Lucentis, Avastin, aflibercept, conbercept, triamcinolone acetonide, steroids, etc);  4) The target eye has received the following intraocular surgical treatments (PDT, vitrectomy, cataract surgery and retinal laser therapy required during the clinical study, periocular vascular bypass surgery, etc.);  5) Currently taking or may require systemic medications that can cause ocular toxicity, such as Psoralen, Risedronate, or Tamoxifen;  6) Hypersensitivity to any component of the investigational drug, or allergic constitution (e.g., allergy to two or more drugs or food);  7) Abnormal findings of physical examination, vital signs, laboratory tests (hematology, urinalysis, blood biochemistry, coagulation function, immunological examination, female blood pregnancy test), 12-lead ECG, chest X-ray judged by the investigator as clinically significant;  8) Having any past or present medical history that may affect the safety of the study or the metabolic process of the drug, especially the medical history of cardiovascular, hepatic, renal, endocrine, gastrointestinal, pulmonary, neurological, hematological, oncologic, immunological or metabolic disorders and others that are thought clinically significant by the investigator;  9) Participation in any clinical study of drug or medical device within 3 months prior to screening;  10) Patients with rAAV neutralizing antibody titer greater than 1:1000 detected by immunogenicity test;  11) Females who are pregnant or lactating;  12) Subjects who are not suitable for participation in this clinical study due to other reasons judged by the investigator. |
| **Sample Size:** 3 + 9 expected |
| **Study Procedure:**  Eligible subjects will be administered prednisone 3 days (Day -3) prior to dosing for 10 days at a dose of 1 mg/kg/day for the first 3 days. On the day of ZVS101e administration, vitrectomy is performed on the target eye according to the clinical practice, and 150 uL of the ZVS101e is injected into the subretinal space through a sterile injection needle for single use in strict accordance with the package insert of the drug. After surgery, routine treatment after vitrectomy is performed and prednisone is orally administered at 0.5 mg/kg/day for an additional 7 days without any treatment in the fellow eye. Patients have to lie supine for at least 14 hours following treatment (according to the clinical study results of Luxturna, 150 uL of the treatment vector was completely absorbed approximately 14 hours after local injection).  On D1, D2, D3, D7, D14, D28, D60, D90, D180, D270 and D365 after injection and at 15 months after the end of observation period, safety assessments were performed at corresponding time points, adverse events and concomitant medications are recorded for safety evaluation.  According to the blood collection points, biological samples are collected for relevant safety and immunogenicity evaluation.  Ocular functional tests (BCVA- ETDRS chart, macular microperimetry, mfERG, dark-adapted contrast sensitivity, and MLMT) and morphological test (color fundus photography, fundus autofluorescence (FAF) and OCT) are performed at visit points during the study to preliminarily evaluate the efficacy of the vector. |
| **Biological sample collection and testing:**  **Collection Points for rAAV Vector DNA Detection in Lacrimal Fluid:**  Tear is collected from subjects at pre-dose (baseline period), 3 h (D0), D1, D2, D3, D7, and D365 post-dose, and 15 months after the end of observation period to extract DNA. DNA samples are used to test the DNA concentration of rAAV vector and quantified using a validated real-time quantitative PCR (q-PCR).  **Collection Points for rAAV Vector DNA Detection in Peripheral Blood:**  Two mL of blood samples are collected from the upper limb vein of subjects and put into EDTA-anticoagulated blood collection tube (blood collection tube with purple cap) to separate leukocytes and extract DNA at each point at pre-dose (baseline period), 3 h (D0), D1, D7 and D365 post-dose, and 15 months after the end of observation period. DNA samples are used to test the DNA concentration of rAAV vector and quantified using a validated real-time quantitative PCR (q-PCR).  **Immunogenicity Blood Collection Points:**  Ten mL of blood samples are collected from the upper limb vein of subjects at each point at pre-dose (baseline period), D14, D28 and D365 post-dose, and 15 months after the end of observation period, and placed in BD 362761 peripheral blood mononuclear cell (PBMC) preparation tubes (CPT) to isolate PBMCs. PBMCs are used to test T cell immune response against rAAV vectors and T cell immune response against CYP4V2 protein, which are quantified using a validated ELISpot method.  Two mL of blood samples are collected from the upper limb vein of subjects and put into EDTA-anticoagulated blood collection tube (blood collection tube with purple cap) to obtain serum samples at each collection point at pre-dose (baseline period), D14, D28, D90 and D365 post-dose, and 15 months after the end of observation period. Plasma samples are used to detect rAAV vector neutralizing antibodies and quantified using a validated enzyme-linked immunosorbent assay (ELISA).  Two mL of blood samples are collected from the upper limb vein of subjects and put into blood collection tube containing coagulant (blood collection tube with orange-yellow cap) to obtain serum samples at each collection point at pre-dose (baseline period), D14, D28, D90 and D365 post-dose, and 15 months after the end of observation period Serum samples are used to detect humoral immune responses against CYP4V2 protein and quantified using a validated enzyme-linked immunosorbent assay (ELISA). |
| **Safety** **Evaluation:**  1) Adverse events: Ocular hypertension (excluding intraocular pressure slightly higher within postoperative 24 hours), endophthalmitis, vitreous hemorrhage, subretinal hemorrhage, retinal detachment (excluding within postoperative 14 hours), macular hole;  2) Ophthalmological examination:BCVA, intraocular pressure test (IOP, intraocular pressure in both eyes), slit-lamp examination, ophthalmoscopy, color fundus photography, FAF and OCT;  3) Vital signs, physical examination, chest X-ray and ECG;  4) General laboratory tests: Blood routine and urine routine, liver function and renal function tests, Serum virology tests, coagulation function test;  5) Vector shedding: Each subject was evaluated according to the concentration of rAAV vector DNA detected in tear and peripheral blood leukocytes.  6) Immunogenicity-: Each subject was evaluated based on cellular and humoral immune response against the therapeutic vector in the blood. |
| **Preliminary Exploratory Measures of Efficacy**  1. Functional assessments:  1) BCVA: Early Treatment Diabetic Retinopathy Study Visual Acuity Scale (ETDRS Chart) is used to examine visual acuity at pre-dose (baseline period), D1, D2, D3, D14, D28, D60, D90, D180, D270 and D365 post-dose, and 15 months after the end of observation period;  2) Color vision test: Atlas for colorblindness is used to assess color vision at pre-dose (baseline period), D28, D60, D180 and D365 post-dose, and 15 months after the end of observation period;  3) Dark-adapted contrast sensitivity test: This test is used to compare the changes in scotopic contrast sensitivity at pre-dose (baseline period), D28, D60, D180 and D365 post-dose, and 15 months after the end of observation period;  4) Macular microperimetry and mfERG: This examination is used to compare the changes in macular retinal function and macular microperimetry at pre-dose (baseline period), D28, D60, D180 and D365 post-dose, and 15 months after the end of observation period;  2. Behavioral assessments:  MLMT is used to test the behavioral changes of subjects at pre-dose (baseline period), D28, D60, D180 and D365 post-dose, and 15 months after the end of observation period;  3. Morphological assessments:  Color fundus photography, fundus autofluorescence (FAF) and OCT are used to examine changes of retinal morphology and retinal cells in each layer at pre-dose (baseline period), D1, D2, D3, D14, D28, D60, D90, D180, D270 and D365 post-dose, and 15 months after the end of observation period. |
| **Data Processing and Statistical Analysis:**  **1.** Safety parameters will be calculated based on Winnonlin software. All other statistical analyses will be performed using SAS 9.4 or above version. Significance level is set as two-sided 0.05.  **2.** Descriptive statistics will be performed on basic data such as subject characteristics, number of subjects enrolled, and dropouts.  **3.** Safety analysis: The incidences of various adverse events (ocular or non-ocular) in subjects are statistically analyzed, and descriptive statistical analysis is performed for vital signs, ophthalmological examination, laboratory test and other safety measures.  Descriptive statistical analysis is performed on concentrations in tear and plasma and pharmacokinetic parameters following a single subretinal injection at this dose level.  Descriptive statistical analyses are performed for cellular immune responses in blood PBMCs, concentrations of rAAV antibodies and CYP4V2 protein antibodies in blood following a single subretinal injection at this dose level.  **4.** Efficacy analysis: Descriptive statistical analysis is performed for each efficacy measure by time, and the changes from baseline in efficacy measures at each visit after administration are calculated. Whether there are significant differences in the changes of best-corrected visual acuity, color vision, dark-adapted contrast sensitivity, macular microperimetry, mfERG and behavior from baseline at each time point after administration is tested based on the analysis of covariance model. |

# Table 1. Study Flow Chart

|  | Screening/Baseline period | Observation period | | | | | | | | | | | | | Follow-up period |
| --- | --- | --- | --- | --- | --- | --- | --- | --- | --- | --- | --- | --- | --- | --- | --- |
| Time of visit | V1 | V2 | V3 | V4 | V5 | V6 | V7 | V8 | V9 | V10 | V11 | V12 | V13 | V14 | V15 |
|  | B1  (1w pre-op) | D-3 | D0 | D1 | D2 | D3 | D7 | D14  ±2d | D28  ±5d | D60  ±5d | D90  ±5d | 180  ±30d | D270  ±30d | D365^8^  ±30d | 15 months ± 30 d after the end of observation period |
| Sign informed consent form | X |  |  |  |  |  |  |  |  |  |  |  |  |  |  |
| Demographic data | X |  |  |  |  |  |  |  |  |  |  |  |  |  |  |
| Medical and treatment history | X |  |  |  |  |  |  |  |  |  |  |  |  |  |  |
| Verification of inclusion/exclusion criteria | X |  |  |  |  |  |  |  |  |  |  |  |  |  |  |
| Physical examination | X |  |  | X |  |  |  |  | X |  | X |  |  | X | X |
| Serum pregnancy test^1^ | X |  |  |  |  |  |  |  |  |  |  |  |  |  |  |
| Start of prednisone administration |  | X |  |  |  |  |  |  |  |  |  |  |  |  |  |
| Surgical administration |  |  | X |  |  |  |  |  |  |  |  |  |  |  |  |
| End of prednisone administration |  |  |  |  |  |  | X |  |  |  |  |  |  |  |  |
| Vital signs ^2^ | X |  | X | X | X | X | X | X | X | X | X | X | X | X | X |
| ECG | X |  | X | X |  |  | X | X | X | X | X | X | X | X | X |
| Chest X-ray | X |  |  |  |  |  |  |  | X |  |  |  |  | X | X |
| Blood routine^3^ | X |  |  | X |  |  | X | X | X | X |  | X |  | X | X |
| Urine routine^3^ | X |  |  | X |  |  | X | X | X | X |  | X |  | X | X |
| Blood chemistry^3^ | X |  |  | X |  |  | X | X | X | X |  | X |  | X | X |
| Serum virology tests^3^ | X |  |  | X |  |  | X |  | X |  |  |  |  | X | X |
| Coagulation function test^3^ | X |  |  | X |  |  | X |  | X |  |  |  |  | X | X |
| Collection of lacrimal fluid | X |  | X | X | X | X | X |  |  |  |  |  |  | X | X |
| Collection of peripheral blood leukocytes | X |  | X | X |  |  | X |  |  |  |  |  |  | X | X |
| Collection of peripheral blood mononuclear cells^4^ | X |  |  |  |  |  |  | X | X |  |  |  |  | X | X |
| rAAV vector neutralizing antibodies in peripheral blood plasma^5^ | X |  |  |  |  |  |  | X | X |  | X |  |  | X | X |
| CYP4V2 protein antibodies in peripheral blood serum^5^ | X |  |  |  |  |  |  | X | X |  | X |  |  | X | X |
| General ophthalmological examination ^6^ | X |  |  | X | X | X |  | X | X | X | X | X | X | X | X |
| Best-corrected visual acuity^7^ | X |  |  | X | X | X |  | X | X | X | X | X | X | X | X |
| Color fundus photography | X |  |  | X | X | X |  | X | X | X | X | X | X | X | X |
| OCT | X |  |  | X | X | X |  | X | X | X | X | X | X | X | X |
| Color vision | X |  |  |  |  |  |  |  | X | X |  | X |  | X | X |
| Macular microperimetry | X |  |  |  |  |  |  |  | X | X |  | X |  | X | X |
| Contrast sensitivity | X |  |  |  |  |  |  |  | X | X |  | X |  | X | X |
| mfERG | X |  |  |  |  |  |  |  | X | X |  | X |  | X | X |
| MLMT | X |  |  |  |  |  |  |  | X | X |  | X |  | X | X |
| Adverse events |  | X | X | X | X | X | X | X | X | X | X | X | X | X | X |
| Concomitant medications, treatments |  | X | X | X | X | X | X | X | X | X | X | X | X | X | X |

Comments:

1. Blood pregnancy test should be performed within 1 week before administration.

2. Vital signs are measured at the following time points within 24 h after administration: Pre-dose (within 1 h) and 1 h ± 15 min and 3 h ± 30 min post-dose.

3. Laboratory tests (blood routine, urine routine, blood chemistry, Serum virology tests and coagulation function test) should be repeated if it is performed more than 1 week prior to administration.

4. PBMC immunoreactivity is detected by ELISpot method after isolation of PBMC cells from venous blood of patients.

5. Detection of rAAV vector neutralizing antibody and CYP4V2 protein antibody in peripheral blood: Collect venous blood from patients, separate plasma or serum and detect by ELISA. The subsequent detection is stopped if the detection is negative on two consecutive occasions.

6. General ophthalmological examination includes intraocular pressure, slit-lamp examination and ophthalmoscopy (direct ophthalmoscopy, indirect ophthalmoscopy, or preset lens).

7. Best-corrected visual acuity is measured using the ETDRS chart.

8. Patients should be followed up annually after the end of this study.

9. Test items judged as adverse reactions need to be repeated at subsequent visits until recovery or stabilization.

# 1. Study Background

## 1.1 Disease Background and Treatment Status

Bietti's crystalline dystrophy (BCD) is a special type of retinal degeneration with typical change of yellow-white flare crystalline deposits in the retina with atrophy of retinal pigment epithelium and choriocapillaris, which seriously jeopardizes visual acuity. Patients with BCD are rare in the West, with more patients in China and Japan, with a population prevalence of 1/24,000 ^[1]^, based on which it is estimated that there are 70,000 patients who are blind due to BCD in China. It is an important cause of blindness in working-age population ^[2]^ and currently there is no effective therapy.

BCD is inherited as an autosomal recessive trait and is caused by CYP4V2 mutations^[3]^. CYP4V2 gene is one of the proteins in the cytochrome P450 (CYP) superfamily, which is mainly distributed on the endoplasmic reticulum and mitochondrial inner membrane and is a terminal oxygenase involved in the synthesis and metabolism of a variety of endogenous and exogenous substances, as well as an omega-fatty acid hydroxylase that plays an important role in intracellular lipid metabolism ^[4, 5, 6]^. The mechanism of action is shown in Figure 1. CYP4V2 is a selective of omega-3 polyunsaturated medium-chain fatty acid hydroxylase and exhibits unique chain length selectivity with substrate specificity of myristate (C14) > lauric acid (C16) > palmitic acid (C12) ^[7]^; the endogenous substrate is omega-3 polyunsaturated fatty acids, mainly docosahexaenoic acid (DHA) and eicosapentaenoic acid (PA) in the eyes ^[8]^. CYP4V2 mRNA is widely expressed, mainly in retinal pigment epithelium (RPE) and photoreceptor cells in the eyes ^[8]^. Mutations in the CYP4V2 gene in patients with BCD lead to abnormal function of their encoded enzymes, which affects lipid metabolism and causes disease ^[6,7,8,9]^. RPE cells play an important role in the development and visual function of the eye and have important functions such as secreting growth factors, anti-oxidation, participating in visual circulation metabolism, maintaining the blood-retinal barrier, and phagocytosing outer segment membrane discs shed by photoreceptor cells ^[10]^. Lesions in RPE cells lead to retinal photoreceptor cell damage, resulting in retinal degeneration and other diseases.


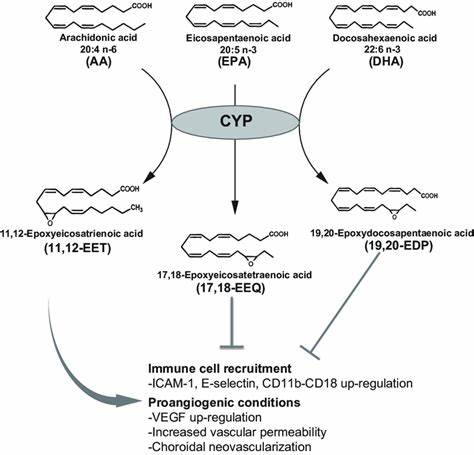


Figure 1. CYP proteins are involved in lipid metabolism of omega-3 polyunsaturated medium-chain fatty acids ^[5]^

Currently, molecular genetic studies have identified multiple mutation sites in the CYP4V2 gene ^[11-15]^. As of August 2019, 86 pathogenic mutations of CYP4V2 gene are currently known, including 56 nonsense mutations, 12 splicing mutations, 11 deletions, 3 insertions, 2 deletions, and 1 large fragment deletion, see Table 2 (The Human Gene Mutation Database, http://www.hgmd.cf.ac.uk/ac/index.php). There is no effective treatment available for BCD worldwide.

Table 2. Summary of CYP4V2 gene mutation sites

| **Exon** | **Mutations** | **Effect** | **Allele frequency in 74 probands** |
| --- | --- | --- | --- |
| E1 | c.65T>A | p.L22H | 1 |
|  | c.214+1G>A | splicing site | 1 |
| E2 | c.215-2A>G | splicing site | 1 |
|  | c.219T>A | p.F73L | 5 |
|  | c.283G>A | p.G95R | 6 |
| E3 | c.335T>G | p.L112* | 2 |
| E4 | c.414-4_414del4bpinsA | splicing site | 1 |
|  | c.518T>G | p.L173W | 1 |
| E6 | c.681_4delTGAG | p.S227Rfs*1 | 1 |
| E7 | c.802-8_810del17bpinsGC | splicing site | 72 |
|  | c.802-8_810del17bpinsGT | splicing site | 5 |
|  | c.958C>T | p.R320* | 2 |
|  | c.965_7delAAG | p.321delE | 1 |
| E8 | c.992A>C | p.H331P | 15 |
|  | c.992A>G | p.H331P | 6 |
|  | c.994G>A | p.D332N | 1 |
|  | c.1062insA | p.V355Sfs*3 | 1 |
|  | c.1062-1063insA | p.V355Sfs*2 | 2 |
| E9 | c.1168C>T | p.R390C | 1 |
|  | c.1169G>A | p.R390H | 5 |
|  | c.1199G>A | p.R400H | 1 |
|  | c.1091-2A>G | splicing site | 15 |
| E10 | c.1126-6_1135del16bp | splicing site | 1 |
| E11 | c.1544T>G | p.I515S | 1 |

In recent years, significant progress has been made in gene therapy for inherited retinal degeneration. On December 20, 2017, FDA approved Luxturna, a RPE65 gene replacement therapy, for marketing. As the first gene replacement therapy drug for hereditary diseases in the history of the United States, Luxturna has opened a new era of gene therapy, brought new hope for the treatment of such diseases, and provided reference for the treatment of other single-gene hereditary diseases. Mutations result in loss of function of the protein encoded by target gene, and complementary DNA (cDNA) of the target gene is packaged into a vector and injected into the subretinal space, which has been found to partially improve visual function in patients in clinical studies ^[16-18]^. In terms of vector selection, recombinant adeno-associated virus vector (rAAV) has become the most commonly used and the most effective gene therapy vector in ophthalmology due to its advantages of good safety, low immunogenicity, ability to infect non-dividing phase cells, expression of exogenous genes for a long time in vivo, and specific tissue affinity for retinal photoreceptor cells. rAAV-mediated gene replacement therapy has demonstrated good safety and clinical effects in foreign clinical studies.

Mutations in the CYP4V2 gene result in loss of protein function and gene replacement therapy is theoretically safe and effective. In the previous study, our company successfully constructed CYP4V2 gene replacement therapy vector. The patient iPSC-RPE cells in vitro and CYP4V2 gene mutation mouse model were used to verify the efficacy and safety at cellular level of replacement therapy vector in mice, all of which achieved significant therapeutic effect without significant toxic and side effects. At present, intellectual property protection has been applied for this product. There are no clinical studies on replacement therapy for BCD worldwide, and the use of rAAV2/8-hCYP4V2 Ophthalmic Injection will fill this gap and bring good news to patients.

Preliminary study results are detailed in the Investigator's Brochure.

## 1.2 Investigational Drug Background

Drug name: rAAV2/8-hCYP4V2 Ophthalmic Injection, developed by Chigenovo Co., Ltd. and provided by OBiO Technology (Shanghai) Corp., Ltd. The internal code is ZVS101e in Chigenovo Co., Ltd., and C035 Injection in OBiO Technology (Shanghai) Corp., Ltd.

rAAV2/8-hCYP4V2 Ophthalmic Injection is a recombinant adeno-associated virus type 8 vector expressing human CYP4V2 protein. The genome includes the ITR of rAAV2 adeno-associated virus and human CYP4V2 gene expression frame, and is 3 kb in length. rAAV2/8-hCYP4V2 vector has a specific tropism for retinal cells, expresses human CYP4V2 protein and normally exerts physiological functions.

rAAV2/8-hCYP4V2 Ophthalmic Injection is prepared by transfection of multiple plasmids into suspended human embryonic kidney cells, large-scale fermentation, and high purification. It contains suitable stabilizers, and no preservatives and antibiotics. Clinical samples are manufactured under current Good Manufacturing Practice (GMP) conditions. The specification is based on the Technical Guidelines for Human Gene Therapy Studies and Drug Product Quality Control and Chinese Pharmacopoeia 2015, and is set with reference to the marketed gene therapy drug Luxturna or other rAAV vectors. Sample testing met specification.

After subretinal injection of rAAV2/8-hCYP4V2 Ophthalmic Injection in patients with BCD, recombinant adeno-associated virus rAAV2/8-hCYP4V2 can efficiently infect retinal cells and express CYP4V2 protein in photoreceptor cells, and the expressed CYP4V2 protein can compensate for the function of the deleted protein, thus playing a huge therapeutic role in the functional recovery of retina.

At present, preclinical studies, including in vitro pharmacodynamics, in vivo pharmacodynamics, pharmacokinetics and toxicology, have been completed for rAAV2/8-hCYP4V2 adeno-associated virus Ophthalmic Injection, and the efficacy and safety of rAAV2/8-hCYP4V2 gene replacement therapy have been preliminarily demonstrated, as detailed in the Investigator's Brochure.

Based on these preclinical study results and clinical practical needs, we intend to enroll patients with BCD to evaluate the safety and preliminary efficacy of rAAV2/8-hCYP4V2 gene replacement therapy and preliminarily explore the large-scale application of rAAV2/8-hCYP4V2 gene replacement therapy technology.

# 2. Study Objectives

**Primary Objective:** To evaluate the safety of a single subretinal injection of rAAV2/8-hCYP4V2 in patients with Bietti's crystalline dystrophy (BCD).

**Secondary Objective:** To preliminarily explore the clinical efficacy of rAAV2/8-hCYP4V2 gene replacement therapy.

# 3. Study Design

## 3.1 Overall Design

This is a single-center, open-label, single-arm and single-dose study and aimed to evaluate the safety and preliminary efficacy of the investigational drug in patient with BCD.

**Selection Criteria for Target Eye**

The target eye must meet the following requirements:

1) BCVA is counting finger to 0.3 (equivalent to Snellen visual counting finger to 20/63); able to perform visual examination and retinal function test; macular photoreceptor layer, that is, the outer nuclear layer can be observed in macular area under standard optical coherence tomography (OCT);

2) The eye with poorer visual acuity in both eyes of the candidate subject is the target eye. Note: For all subjects, only one eye is designated as the "target eye" (i.e., treated eye). If both eyes of patients meet the inclusion criteria, the target eye will be medically determined by the investigator.

**Safety Study**

The investigational product rAAV2/8-hCYP4V2 Ophthalmic Injection is a gene replacement therapy product. In preliminary safety clinical study (ClinicalTrials No.: NCT04722107), enrollment, administration, and 3-month safety and efficacy observation have been completed for 3 subjects. During the observation period, no drug- or treatment-related serious adverse reactions occurred in any of the 3 subjects; meanwhile, 2 of the subjects showed significant improvements in visual acuity and MLMT score. It can be seen that the clinical dose of 5×10^8^ vg/uL has good safety and reflects certain efficacy. In this clinical study, additional 9 subjects are planned to be enrolled, and a total of 1 dose group, 7.5×10^10^ vg, is set. After one subject is enrolled first and no significant toxic and side effects are observed within 14 days after administration, the study in the second subject may be initiated. After the second subject completes administration and no significant toxic and side effects are observed within 14 days, the study in the third subject may be initiated; and so on. While conducting safety study, tear and blood samples are collected from subjects to detect DNA of rAAV2/8 virus and immune responses against the therapeutic vector.

**Preliminary Exploratory Study of Efficacy**

Clinical efficacy is preliminarily assessed by BCVA (ETDRS chart), macular microperimetry, mfERG, color vision, dark-adapted contrast sensitivity, and MLMT.

## 3.2 Rationale for Dose Setting

The investigational drug rAAV2/8-hCYP4V2 Ophthalmic Injection is a gene replacement therapy. In preliminary safety clinical study (ClinicalTrials No.: NCT04722107), enrollment, administration, and 3-month safety and efficacy observation have been completed for 3 subjects. During the observation period, no drug- or treatment-related serious adverse reactions occurred in any of the 3 subjects; meanwhile, 2 of the subjects showed significant improvements in visual acuity and MLMT score. It can be seen that the clinical dose of 5×10^8^ vg/uL has good safety and reflects certain efficacy.

Preclinical pharmacodynamic experiment has preliminarily demonstrated that 1 uL of the following concentrations of rAAV2/8-hCYP4V2 were injected into the subretinal space of CYP4V2 knockout mice: CYP4V2 protein was expressed in 1×10^8^, 5×10^8^, 1×10^9^, 3×10^9^, 6×10^9^ and 1×10^10^ (unit: vg/uL), and the 6-month in vivo observation at following three doses of 3×10^9^ vg, 6×10^9^ vg and 1×10^10^ vg showed that 3×10^9^ vg had a significant therapeutic effect without obvious toxicities, and 6×10^9^ vg and 1×10^10^ vg had evident efficacy in the early stage, but obvious retinal toxicity in the later stage.

Referring to Luxturna (produced by Spark, intended for LCA patients carrying RPE65 gene mutation), the only gene replacement therapy marketed in the field of inherited retinal degeneration, the dose escalation concentration of Luxturna is designed as: 1×10^8^ (150 uL), 3.2×10^8^ (150 uL) and 5×10^8^ (300 uL) (unit: vg/uL). The dose escalation study results showed that all of the above three concentrations had therapeutic effect without obvious toxicities. The recommended Phase 3 concentration of Luxturna was 5×10^8^ vg/uL (300 uL), which was the maximum concentration tested in vivo.

Referring to the clinical study data of RPGR gene replacement therapy drug published in Nature Medicine in February 2020, the dose escalation concentrations were designed as six doses: 5×10^7^ (40-100 uL), 1×10^8^ (40-100 uL), 5×10^8^ (30-100 uL), 1×10^9^ (60-100 uL), 2.5×10^9^ (50-150 uL) and 5×10^9^ (30-80 uL) (unit: vg/uL). The results showed that 5×10^7^ vg/uL and 1×10^8^ vg/uL groups had no significant improvement, 5×10^8^ vg/uL group had significant increase, and 7 of 9 subjects in the three high concentration groups showed mild inflammatory reaction, which could be effectively managed after hormone therapy.

Based on the preclinical results of the investigational drug and comparison with similar drugs, and referring to the dose design for Phase 1 dose escalation and recommended clinical dose of the similar marketed drug Luxturna, the concentration gradient selected in this project is: 1×10^8^ vg/uL, 5×10^8^ vg/uL, 1×10^9^ vg/uL and 3×10^9^ vg/uL, and the injection dose is 150 uL. The concentration selected for this preliminary experiment is 5×10^8^ vg/uL, 150 uL, that is, each subject is injected with a total of 7.5×10^10^ vg of investigational drug.

## 3.3 Definition of End of Study

The study ends when the last subject completes follow-up and the investigator completes all assessments and visit records.

**Required by Investigator**

The investigator may not be able to continue the study for some reasons. The investigator must inform the subject, sponsor, Ethics Committee and clarify the reason for terminating a clinical study.

**Required by Sponsor**

Reasons for sponsor's requirement for early termination of the study or early termination of the study in one study site:

1) Funding reasons;

2) Administrative reasons;

3) The investigator cannot follow the protocol;

4) The investigator cannot recruit enough subjects;

5) Safety considerations.

The sponsor must inform the investigator, Ethics Committee in writing and clarify the reasons before terminating a clinical study.

# 4. Study Population

## 4.1 Inclusion Criteria

Subjects who meet all of the following inclusion criteria are considered eligible for participation in this study.

1) Age ≥ 18 years (inclusive) at the time of informed consent;

2) Patients with clinically confirmed Bietti's crystalline dystrophy (BCD);

3) Carrying two causative mutations in CYP4V2 gene confirmed by genetic testing;

4) Meet selection criteria for target eye;

5) The subject and his/her spouse agree to take effective contraceptive measures during the study and within 1 year after administration;

6) Voluntarily participate in this clinical study and have signed informed consent form.

## 4.2 Exclusion Criteria

Subjects meeting any of the following criteria will not be eligible to participate in this study.

1) The patient lacks sufficient viable retinal photoreceptor cells, has less than 1 disc area of retina photoreceptor cells or has a retinal thickness of less than 100 μm.

2) Active choroidal neovascularisation (CNV) lesion secondary to BCD in the target eye judged by the investigator; or other ocular disorders that would preclude surgery or interfere with interpretation of the study endpoints;

3) Medications within 3 months prior to screening that may affect study observation (e.g., Lucentis, Avastin, aflibercept, conbercept, triamcinolone acetonide, steroids, etc);

4) The target eye has received the following intraocular surgical treatments (PDT, vitrectomy, cataract surgery and retinal laser therapy required during the clinical study, periocular vascular bypass surgery, etc.);

5) Currently taking or may require systemic medications that can cause ocular toxicity, such as Psoralen, Risedronate, or Tamoxifen;

6) Hypersensitivity to any component of the investigational drug, or allergic constitution (e.g., allergy to two or more drugs or food);

7) Abnormal findings of physical examination, vital signs, laboratory tests (hematology, urinalysis, blood biochemistry, coagulation function, immunological examination, female blood pregnancy test), 12-lead ECG, chest X-ray judged by the investigator as clinically significant;

8) Having any past or present medical history that may affect the safety of the study or the metabolic process of the drug, especially the medical history of cardiovascular, hepatic, renal, endocrine, gastrointestinal, pulmonary, neurological, hematological, oncologic, immunological or metabolic disorders and others that are thought clinically significant by the investigator;

9) Participation in any clinical study of drug or medical device within 3 months prior to screening;

10) Patients with rAAV neutralizing antibody titer greater than 1:1000 detected by immunogenicity test;

11) Females who are pregnant or lactating;

12) Subjects who are not suitable for participation in this clinical study due to other reasons judged by the investigator.

## 4.3 Withdrawal Criteria

1) Subject is unwilling or unable to continue to participate in the study (withdrawal of informed consent, non-adverse event reasons);

2) Subject loss to follow-up;

3) Serious non-compliance with study requirements;

4) The subject presents any adverse event (clinical adverse event, laboratory test abnormality) that precludes continuation of the clinical study, diseases or other conditions found during the study;

5) The investigator judges that the subject should withdraw from the study;

6) Other reasons.

The date and reason for subject withdrawal will be recorded on the Case Report Form (CRF). Withdrawer due to adverse events should be followed up until recovery or stabilization.

# 5. Study Intervention

## 5.1 Investigational Drug

### 5.1.1 Investigational drug information

**Name:** rAAV2/8-hCYP4V2 Ophthalmic Injection (the internal code is ZVS101e in Chigenovo Co., Ltd., and C035 Injection in OBiO Technology (Shanghai) Corp., Ltd.)

**Strength:** 1.0×10^12^ vg/mL, 0.3 mL/vial.

**Route of Administration:** Subretinal injection.

**Storage Conditions:** Long-term storage at ≤ -70°C, short-term storage at 2-8°C. Stored in the original carton in the dark until use.

**R&D Unit/Supplier:** Chigenovo Co., Ltd.

**Manufacturer:** OBiO Technology (Shanghai) Corp., Ltd.

### 5.1.2 Dosing regimen

In this clinical study, additional 9 subjects are planned to be enrolled, and a total of 1 dose group, 7.5×10^10^ vg, is set. After one subject is enrolled first and no significant toxic and side effects are observed within 14 days after administration, the study in the second subject may be initiated. After the second subject completes administration and no significant toxic and side effects are observed within 14 days, the study in the third subject may be initiated; and so on. While conducting safety study, lacrimal fluid and blood samples are collected from subjects to detect DNA of rAAV2/8 virus and immune responses against the therapeutic vector.

### 5.1.3 Packaging and labeling

Packaging: The investigational drug is packaged by the cooperative unit.

Labeling: The label should be indicated with product name, batch number, strength, shelf life, storage condition, entrusting party, manufacturer, "For clinical study use only", etc.

### 5.1.4 Drug storage and management

The investigational drug will be uniformly transported by the sponsor, and directly distributed to the study site by a specially-assigned person. According to the complete drug receipt procedures of the study site, there is a specially-assigned person to accept the drug and sign the drug receipt form.

The study site has established a strict drug management system (specially-assigned person, special cabinet and locked storage) to be responsible for the storage and distribution of investigational drug and establish the registration system. The study site should ensure that the storage environmental conditions (temperature, light, humidity, etc.) of investigational drug are compliant with regulations, and it should be recorded and stored. If the storage conditions are found beyond the specified range, contact the sponsor immediately.

Use of medications should be recorded on the appropriate log sheet.

At the end of the study, all investigational drugs, including all unused, partially used study drug and all drug packages returned, with completed and signed drug return log, at the investigator should be returned to the sponsor.

## 5.2 Concomitant Therapies

**5.2.1 Permitted concomitant therapies**

The following concomitant therapies are permitted during the study, including the screening period. All concomitant medications received by the subjects and reasons for medication (generic name, purpose of administration, dose, administration time, etc.) should be completely recorded in the original data, eCRF.

1) Routine medications for eye operation: Fluorescein Sodium, Indocyanine Green, local anesthetics (including retrobulbar anesthetics for avoiding eye movement), local antibiotics, mydriatic agents;

2) Medications for preventing nausea caused by injection of Fluorescein Sodium and Indocyanine Green;

3) Medications or therapies required for other past diseases;

4) Other disease-related supportive therapies;

5) The investigator may give appropriate treatment for adverse events occurring during the study.

**5.2.2 Prohibited concomitant therapies**

The following concomitant therapies are prohibited during the study, including the screening period.

1) Other medications for BCD (including Lucentis and Avastin, etc.);

2) Drugs with toxicity to lens, retina, and optic nerve, including Desferrioxamine, Chloroquine/Hydroxychloroquine, Tamoxifen, Phenothiazine, Ethambutol, etc.;

3) Surgery: Retinal laser therapy, periocular vascular bypass surgery, photodynamic therapy, macular foveal laser photocoagulation, vitrectomy, submacular surgery, cataract surgery, or other surgical intervention.

**5.2.3 Overdose**

Referring to the clinical study data of Luxturna (produced by Spark, intended for LCA patients carrying RPE65 gene mutation), the only gene replacement therapy marketed in the field of inherited retinal degeneration, and RPGR gene replacement therapy drug published in Nature Medicine in February 2020, overdose has the risk of causing local mild inflammatory reactions in the retina and corticosteroids can be given if necessary for effective control.

Overdose of investigational drug due to any condition should be truthfully recorded, its causes should be analyzed, taking corresponding actions to avoid recurrence.

**5.2.4 Rescue therapy**

There is no specific rescue medication or therapeutic measure for the investigational drug. In case of emergency in clinical practice, it can be closely observed and symptomatic treatment can be given according to the applicable clinical diagnosis and treatment process.

**5.2.5 Recording of concomitant medications**

Concomitant medications refer to all medications (including traditional Chinese medicines) administered, except for investigational drug and are recorded as follows: Generic name, indication, dose, usage, start and end time of treatment, etc.

# 6. Study Flow and Study Procedure

## 6.1 Study Flow

### 6.1.1 Screening baseline visit

The following contents should be completed during the screening period.

### (V1, 1 week pre-op)

1) Sign informed consent form

It must be performed before all screening tests; informed consent form must be signed before the subject is enrolled in the study and before any protocol-specified procedures are performed. Each subject will be given a unique subject identification number (subject screening number) when obtaining the informed consent form.

2) Collect demographic data:

Data including gender, age, ethnicity and other information should be collected during the screening period. The study personnel must photocopy the subject's identification card, record the mailing address, contact number, emergency contact person and contact number, and these original records shall be retained at the study site only.

3) Collect medical history and treatment history

Detailed information should be obtained during the screening period about the subject's history of ophthalmological diseases, treatment history, medical history of other major organs, including past medical history, menopause history, surgical history, smoking and drinking history, and allergy history, and the use of all medications within 3 months prior to screening should be recorded.

4) Verification of inclusion and exclusion criteria

5) Physical examination

6) Vital signs

7) ECG and chest X-ray

8) Laboratory tests: Blood routine/urine routine/blood chemistry; Serum virology tests; coagulation function; serum pregnancy test;

9) Biological sample collection and testing: Collection of tear, peripheral blood leukocytes and peripheral blood mononuclear cells, detection of rAAV vector neutralizing antibodies in peripheral blood plasma and CYP4V2 protein antibodies in peripheral blood serum

10) General ophthalmological examination: Including intraocular pressure, slit-lamp examination and ophthalmoscopy

11) BCVA (best-corrected visual acuity) assessment

12) Color fundus photography and FAF

13) OCT (optical coherence tomography)

14) Color vision test

15) Macular microperimetry

16) Contrast sensitivity test

17) mfERG examination

18) MLMT behavioral test

### 6.1.2 Observation period

V2 (D-3), the following items should be completed:

1) Prednisone is administered orally at a dose of 1 mg/kg/day;

2) Adverse events, concomitant medications, treatments

V3 (D0), the following items should be completed:

1) Vital signs (within 1 h pre-dose and 1 h ± 15 min, 3 h ± 30 min post-dose)

2) Prednisone is administered orally at a dose of 1 mg/kg/day

3) Subretinal injection

4) ECG (3 h ± 30 min post-dose)

5) Biological sample collection and testing (3 h ± 30 min post-dose): Collection of tear and peripheral blood leukocytes

6) Adverse events, concomitant medications, treatments

V4 (D1), the following items should be completed:

1) Physical examination

2) Vital signs

3) ECG

4) Laboratory tests: Blood routine/urine routine/blood chemistry; Serum virology tests; coagulation function;

5) Biological sample collection and testing: Collection of tear and peripheral blood leukocytes

6) General ophthalmological examination: Including intraocular pressure, slit-lamp examination and ophthalmoscopy

7) BCVA assessment

8) Color fundus photography and FAF

9) OCT (optical coherence tomography)

10) Prednisone is administered orally at a dose of 0.5mg/kg/day

11) Adverse events, concomitant medications, treatments

V5 (D2), the following items should be completed:

1) Vital signs

2) Biological sample collection: Collection of lacrimal fluid

3) General ophthalmological examination: Including intraocular pressure, slit-lamp examination and ophthalmoscopy

4) BCVA (best-corrected visual acuity) assessment

5) Color fundus photography and FAF

6) OCT (optical coherence tomography)

7) Prednisone is administered orally at a dose of 0.5 mg/kg/day

8) Adverse events, concomitant medications, treatments

V6 (D3), the following items should be completed:

1) Vital signs

2) Biological sample collection and testing: Collection of tear

3) General ophthalmological examination: Including intraocular pressure, slit-lamp examination and ophthalmoscopy

4) BCVA (best-corrected visual acuity) assessment

5) Color fundus photography and FAF

6) OCT (optical coherence tomography)

7) Prednisone is administered orally at a dose of 0.5mg/kg/day

8) Adverse events, concomitant medications, treatments

V7 (D7), the following items should be completed:

1) Prednisone is administered orally at a dose of 0.5 mg/kg/day, last dose

2) Vital signs

3) ECG

4) Laboratory tests: Blood routine/urine routine/blood chemistry; Serum virology tests; coagulation function;

5) Biological sample collection and testing: Collection of tear and peripheral blood leukocytes

6) Adverse events, concomitant medications, treatments

V8 (D14 ± 2d), the following items should be completed:

1) Vital signs

2) ECG

3) Laboratory tests: Blood routine/urine routine/blood chemistry;

4) Biological sample collection and testing: Collection of peripheral blood mononuclear cells, detection of rAAV vector neutralizing antibodies in peripheral blood plasma and CYP4V2 protein antibodies in peripheral blood serum

5) General ophthalmological examination: Including intraocular pressure, slit-lamp examination and ophthalmoscopy

6) BCVA (best-corrected visual acuity) assessment

7) Color fundus photography and FAF

8) OCT (optical coherence tomography)

9) Adverse events, concomitant medications, treatments

V9 (D28 ± 5d), the following items should be completed:

1) Physical examination

2) Vital signs

3) ECG and chest X-ray

4) Laboratory tests: Blood routine/urine routine/blood chemistry; Serum virology tests; coagulation function;

5) Biological sample collection and testing: Collection of peripheral blood mononuclear cells, detection of rAAV vector neutralizing antibodies in peripheral blood plasma and CYP4V2 protein antibodies in peripheral blood serum

6) General ophthalmological examination: Including intraocular pressure, slit-lamp examination and ophthalmoscopy

7) BCVA (best-corrected visual acuity) assessment

8) Color fundus photography and FAF

9) OCT (optical coherence tomography)

10) Color vision test

11) Macular microperimetry

12) Contrast sensitivity test

13) mfERG examination

14) MLMT behavioral test

15) Adverse events, concomitant medications, treatments

V10 (D60 ± 5d), the following items should be completed:

1) Vital signs

2) ECG

3) Laboratory tests: Blood routine/urine routine/blood chemistry

4) General ophthalmological examination: Including intraocular pressure, slit-lamp examination and ophthalmoscopy

5) BCVA (best-corrected visual acuity) assessment

6) Color fundus photography and FAF

7) OCT (optical coherence tomography)

8) Color vision test

9) Macular microperimetry

10) Contrast sensitivity test

11) mfERG examination

12) MLMT behavioral test

13) Adverse events, concomitant medications, treatments

V11 (D90 ± 5d), the following items should be completed:

1) Physical examination

2) Vital signs

3) ECG

4) Biological sample collection and testing: Detection of rAAV vector neutralizing antibodies in peripheral blood plasma and CYP4V2 protein antibodies in peripheral blood serum

5) General ophthalmological examination: Including intraocular pressure, slit-lamp examination and ophthalmoscopy

6) BCVA (best-corrected visual acuity) assessment

7) Color fundus photography and FAF

8) OCT (optical coherence tomography)

9) Adverse events, concomitant medications, treatments

V12 (D180 ± 30d), the following items should be completed:

1) Vital signs

2) ECG

3) Laboratory tests: Blood routine/urine routine/blood chemistry

4) General ophthalmological examination: Including intraocular pressure, slit-lamp examination and ophthalmoscopy

5) BCVA (best-corrected visual acuity) assessment

6) Color fundus photography and FAF

7) OCT (optical coherence tomography)

8) Color vision test

9) Macular microperimetry

10) Contrast sensitivity test

11) mfERG examination

12) MLMT behavioral test

13) Adverse events, concomitant medications, treatments

V13 (D270 ± 30d), the following items should be completed:

1) Vital signs

2) ECG

3) General ophthalmological examination: Including intraocular pressure, slit-lamp examination and ophthalmoscopy

4) BCVA (best-corrected visual acuity) assessment

5) Color fundus photography and FAF

6) OCT (optical coherence tomography)

7) Adverse events, concomitant medications, treatments

V14 (D365 ± 30d), the following items should be completed:

1) Physical examination

2) Vital signs

3) ECG and chest X-ray

4) Laboratory tests: Blood routine/urine routine/blood chemistry; Serum virology tests; coagulation function;

5) Biological sample collection and testing: Collection of tear, peripheral blood leukocytes and peripheral blood mononuclear cells, detection of rAAV vector neutralizing antibodies in peripheral blood plasma and CYP4V2 protein antibodies in peripheral blood serum

6) General ophthalmological examination: Including intraocular pressure, slit-lamp examination and ophthalmoscopy

7) BCVA (best-corrected visual acuity) assessment

8) Color fundus photography and FAF

9) OCT (optical coherence tomography)

10) Color vision test

11) Macular microperimetry

12) Contrast sensitivity test

13) mfERG examination

14) MLMT behavioral test

15) Adverse events, concomitant medications, treatments

### 6.1.3 Follow-up period

V15 (15 months ± 30 days after the end of observation period), the following items should be completed:

1) Physical examination

2) Vital signs

3) ECG and chest X-ray

4) Laboratory tests: Blood routine/urine routine/blood chemistry; Serum virology tests; coagulation function;

5) Biological sample collection and testing: Collection of tear, peripheral blood leukocytes and peripheral blood mononuclear cells, detection of rAAV vector neutralizing antibodies in peripheral blood plasma and CYP4V2 protein antibodies in peripheral blood serum

6) General ophthalmological examination: Including intraocular pressure, slit-lamp examination and ophthalmoscopy

7) BCVA (best-corrected visual acuity) assessment

8) Color fundus photography and FAF

9) OCT (optical coherence tomography)

10) Color vision test

11) Macular microperimetry

12) Contrast sensitivity test

13) mfERG examination

14) MLMT behavioral test

15) Adverse events, concomitant medications, treatments

In case of premature termination of the study, the following items should be completed:

1) Vital Signs and Physical Examination

2) Laboratory tests: Blood routine/urine routine/blood chemistry

3) 12-Lead ECG and chest X-ray

4) General ophthalmological examination

5) Color fundus photography and FAF

6) OCT

7) Macular microperimetry, contrast sensitivity, mfERG, MLMT

8) Biological sample collection and testing: Collection of tear, peripheral blood leukocytes and peripheral blood mononuclear cells, detection of rAAV vector neutralizing antibodies in peripheral blood plasma and CYP4V2 protein antibodies in peripheral blood serum

9) Adverse events, concomitant medications, treatments.

Comments:

1) The test items during the study are detailed in Appendix 3.

2) Sample collection, processing and shipment procedures during the study are detailed in Sample Management Manual.

3) Biological sample collection time windows are detailed in Sample Management Manual.

4) During the study, in order to protect the safety of subjects, the investigator may increase the safety-related study procedure or examination number of such measures according to the actual situation, instead of limiting to the examination frequency specified in the study flow, and the examination results will be recorded in the subject's original documents.

## 6.2 Study Procedure

### 6.2.1 Sign informed consent form

Informed consent form must be signed before the subject is enrolled in the study and before any protocol-specified procedures are performed. Each subject will be given a unique subject identification number (subject screening number) when obtaining the informed consent form.

Before signing the informed consent form, the investigator or designated study personnel should give the subject sufficient time and opportunity to understand the details of the study and answer all questions related to the study raised by the subject in detail.

During the informed consent process, the investigator must comply with regulatory requirements of the drug regulatory authority, the Declaration of Helsinki and local regulations.

During the informed consent process, if the subject or his/her legal representative lacks reading ability, an impartial witness must assist and witness the informed consent.

No written or oral information related to the study shall be used in any language that causes the subject and his/her legal representative to waive his/her legal rights and interests, nor shall it contain any language that exempts the investigator and his/her medical institution, sponsor and his/her agent from their responsibilities.

The investigator should explain the contents of the informed consent form and other written materials to the subject or his/her legal representative and witness in detail. If the subject or his/her legal representative orally agrees to participate in the study, he/she should sign the informed consent form, and the witness must sign and date the informed consent form to prove that the subject or his/her legal representative has accurately explained the informed consent form and other written materials by the investigator, understands the relevant contents, and agrees to participate in the clinical study.

The subject or his/her legal representative shall receive a copy of signed and dated informed consent form and other written materials provided to the subject; during the study, the subject or his/her legal representative shall receive a copy of the updated informed consent form signed and dated, as well as the revised text of other written materials.

When the legal representative gives informed consent on behalf of the subject, the subject should be informed and helped understand the relevant information of the clinical study as far as possible, and the subject should personally sign and date the informed consent form as far as possible.

### 6.2.2 Collection of demographic data

Data including gender, age, ethnicity and other information of subjects should be collected during the screening period. The study personnel must photocopy the subject's identification card, record the mailing address, contact number, emergency contact person and contact number, and these original records shall be retained at the study site only.

### 6.2.3 Collection of medical history and treatment history

Collect the ophthalmic medical history, ophthalmologic treatment history, systemic and other vital organ medical history and surgical history, smoking and drinking history and allergic history of subjects, and record the use of all drugs within 6 months prior to screening.

### 6.2.4 Verification of inclusion and exclusion criteria

Collect the clinical data and genetic diagnosis results of patients, and confirm that patients meet the inclusion criteria of this study according to the inclusion criteria and exclusion criteria, and there are no conditions listed in the exclusion criteria.

### 6.2.5 See Appendix 3 for the test items during the study.

### 6.2.6 See Sample Management Manual for sample collection, processing and shipment procedures during the study in details.

### 6.2.7 See Sample Management Manual for biological sample collection time windows in details.

### 6.2.8 Documentation of concomitant medications

Concomitant medications refer to all medications (including traditional Chinese medicines) administered, except for investigational drug and are recorded as follows: generic name, indication, dose, usage, start and end time of treatment, etc.

At the screening visit, the investigator should ask the subject in detail about the treatment for ophthalmic diseases prior to screening and the use of all medications or therapies within 3 months prior to screening to ensure that the subject meets all inclusion and none of the exclusion criteria, and record the information obtained in the eCRF. Also, long-term medications taken by the subject at screening for chronic disease should be recorded.

During the observation period and at subsequent visits during the observation period, the investigator should carefully ask and record the recent medications or therapies received by the subject. If there is any change in dose or other of long-term medication taken by a subject, the reason for the change and details should be recorded in the eCRF. If a subject needs to take any new medication, he/she should consult the investigator as timely as possible before taking the medication. If the subject fails to reach the investigator in time for special reasons, he/she should inform the investigator faithfully at the next visit at the latest.

# 7. Study Evaluation

## 7.1. Safety Evaluation

Safety evaluation includes adverse events, vital signs, physical examination, ophthalmological examination, laboratory examination, ECG and immunogenicity.

1) Adverse events: Ocular hypertension (excluding slightly high intraocular pressure within 24 hours postoperative), endophthalmitis, vitreous hemorrhage, subretinal hemorrhage, retinal detachment (excluding within 14 hours postoperative), macular hole;

2) Ophthalmological examination: Visual acuity test, intraocular pressure test (intraocular pressure in both eyes), slit-lamp examination, ophthalmoscopy, color fundus photography and OCT;

3) Vital signs, physical examination, chest X-ray and ECG;

4) General laboratory tests: Blood routine and urine routine, liver function and renal function tests, serum virology tests, coagulation function test.

5) Drug-time curves and mean drug-time curves are plotted for each subject based on rAAV vector DNA concentrations measured in lacrimal fluid and peripheral blood leukocytes at each time point.

6) Immune-time curves are plotted for each subject based on cellular and humoral immune data measured for the therapeutic vector in the blood of subjects in the study.

In addition to observation of systemic adverse events/reactions, the focus is on ocular adverse events/reactions. Criteria for grading ocular adverse events are shown in Appendix 1. Definition of serious (sight-threatening) ocular adverse events is detailed in Appendix 2. Ocular adverse events should be assessed as injection-related complications or drug-related.

## 7.2 Preliminary Efficacy Exploratory Measures

1) Functional measures:

A. BCVA: Early Treatment Diabetic Retinopathy Study Visual Acuity Scale (ETDRS Chart) is used to examine and compare the changes in visual acuity at each time point at pre-dose (baseline period), D1, D2, D3, D14, D28, D60, D90, D180, D270 and D365 post-dose, and 15 months after the end of observation period;

B. Color vision test: Atlas for colorblindness is used to compare the changes in color vision at each time point at pre-dose (baseline period), D28, D60, D180 and D365 post-dose, and 15 months after the end of observation period;

C. Dark-adapted contrast sensitivity test: This test is used to compare the changes in scotopic contrast sensitivity at each time point at pre-dose (baseline period), D28, D60, D180 and D365 post-dose, and 15 months after the end of observation period;

D. Macular microperimetry and mfERG examination: This examination is used to compare the changes in macular retinal function and macular microperimetry at each time point at pre-dose (baseline period), D28, D60, D180 and D365 post-dose, and 15 months after the end of observation period;

2) Behavioral measures:

MLMT test is used to compare the behavioral changes of subjects at each time point at pre-dose (baseline period), D28, D60, D180 and D365 post-dose, and 15 months after the end of observation period;

3) Morphological measures:

Color fundus photography, FAF and OCT results are used to examine and compare the changes in retinal morphology and changes in retinal cells in each layer at each time point at pre-dose (baseline period), D1, D2, D3, D14, D28, D60, D90, D180, D270 and D365 post-dose, and 15 months after the end of observation period;

# 8. Adverse Events and Serious Adverse Events

## 8.1 Adverse Events

Definition: An adverse event refers to any undesirable experience occurred by a patient during the study, whether or not related to the study. Adverse events include all unforeseen signs, symptoms, or aggravation of accompanying symptoms or complications.

**Adverse events assessments**

**Severity:** Adverse events are divided into mild, moderate and severe

1) Mild: Symptomatic but not interfering with usual activities;

2) Moderate: Symptomatic and having a slight impact on activities of daily life;

3) Severe: Serious impact on activities of daily life.

**Relationship to the observed drug:** The relationships between adverse events and the observed drug are as follows:

1) Definitely not related: The adverse event is related to other factors such as primary disease, complication, concomitant medication or treatment without temporal relationship to the observed drug.

2) Unlikely related: There is no clear temporal relationship to the observed drug, or it may be related to other factors such as primary disease, complications, concomitant medication or treatment, and the possibility that the observed drug caused it cannot be completely ruled out.

3) Possibly related: There is clear temporal relationship to the observed drug. It is highly suspected that the adverse event is related to the observed drug, and other factors such as primary disease, complications, concomitant medication or treatment cannot be ruled out.

4) Probably related: There is clear temporal relationship to the observed drug. Other factors such as primary disease, complications, concomitant medication or treatment can almost be ruled out.

5) Definitely related: There is clear temporal relationship to the observed drug. Others, such as primary disease, complications, concomitant medication or treatment, may be completely ruled out. The adverse event reappeared after re-administration.

The above items 3-5 should be considered as drug-related adverse reactions and handled as adverse drug reactions. When belonging to items 1-2, it should be detailed in the case report form.

**Method of recording adverse events:**

The investigator should record any undesirable symptoms, diseases or signs experienced by the subject during treatment with the observed drug in the adverse event item of the case report form. The following items need to be recorded:

1) Name and symptoms;

2) Severity;

3) Time of appearance and disappearance;

4) Impact on investigational drug dose:

a: Dose unchanged b: Dose increased c: Dose decreased d: Dose interrupted e: Drug withdrawn.

5) Action taken for the adverse event:

a: No; b: Yes (record concomitant medications and treatments if yes)

6) Outcome after the end of the study:

a: Disappeared; b: Improved; c: Unchanged; d: Unknown.

7) Causal relationship to the observed drug.

Any adverse event that does not disappear after the end of treatment should be followed up until stabilization and safety ensured.

## 8.2 Serious Adverse Event

Definition: Fatal; probably life-threatening; requires hospitalization or prolonged hospitalization; results in serious illness or permanent impairment; results in congenital anomaly.

# 9. Statistical Considerations

## 9.1 Statistical Hypothesis and Sample Size Estimation

### 9.1.1 Statistical hypothesis

This study is an exploratory study on the trend of safety and efficacy, without statistical hypothesis.

### 9.1.2 Sample size estimation

It is estimated to add 9 subjects based on 3 subjects in the preliminary study, 12 subjects in total, with 1 dose group.

## 9.2. Analysis Populations

The statistical analysis data sets for this study are defined as follows.

Full Analysis Set (FAS): According to the intention-to-treat (ITT) principle, all subjects who sign informed consent form and are eligible for screening will be included in the full analysis set of this study, except for major GCP violations.

Safety Set (SS): Including subjects in FAS who use the investigational drug and have safety assessment.

Per-Protocol Set: Including subjects in the FAS who have no major protocol deviations that may significantly affect the primary efficacy endpoint of the study, so subjects with major protocol deviations must be excluded from this analysis population (dose adjustment or discontinuation due to toxicity is not a protocol deviation). The definition of major protocol deviations is discussed and determined prior to the data review meeting. At this meeting, compliance and other protocol violations are used to identify major protocol violations.

All analysis sets will be decided at a data review meeting.

## 9.3 Statistical Analysis

### 9.3.1 General methods

Pharmacokinetic parameters are calculated using WinNonlin 6.4 software, and other statistical analyses are programmed using SAS software. Statistical tests are performed using two-sided tests, with a P value < 0.05 of being considered statistically significant difference. A 95% confidence level is used for confidence interval.

### 9.3.2 Description of baseline conditions

Descriptive statistical analysis will be performed for subjects' age, ethnicity, gender, height, weight, body mass index and other data. Measurement variables will be described by mean, standard deviation, minimum, median and maximum, and enumeration variables will be described by number of cases and percentage.

### 9.3.3 Safety

The incidences of overall and various adverse events at this dose level are statistically analyzed, and descriptive statistical analysis is performed for safety measures such as laboratory tests, vital signs, ophthalmological examination, and ECG.

Descriptive statistical analysis for rAAV vector DNA concentrations in tear and peripheral blood leukocytes after a single dose at each dose level is performed.

Descriptive statistical analysis is performed for the positive rate of anti-drug antibody at this dose level.

Anti-CYP4V2 protein antibody concentration, rAAV2/8 virus neutralizing antibody concentration, cellular immune responses to rAAV2/8 capsid protein and CYP4V2 protein.

### 9.3.4 Efficacy

All efficacy data will be analyzed based on the Full Analysis Set and Per-Protocol Set.

Best-corrected visual acuity, color fundus photography, FAF, OCT, color vision, macular microperimetry, contrast sensitivity, mfERG, and MLMT are tabulated, and the change values from baseline at each test time point for each measure are listed.

Descriptive statistical analysis will be performed for each efficacy measure, and the change in efficacy measures at each visit after administration from baseline will be calculated. The analysis of covariance model will be used to test whether there is significant difference in the changes in best-corrected visual acuity (BCVA), FAF, OCT, color vision, macular microperimetry, contrast sensitivity, mfERG, and MLMT from baseline at different time points after administration.

# 10. Data Management

In order to ensure the safety of subjects and accuracy, completeness and reliability of data throughout the study, the investigator retains laboratory test reports, clinical records and patient medical records as the study source documents in the patient files.

# 11. Study Quality Control and Assurance

In order to ensure that this study is carried out in strict accordance with the clinical study protocol, the clinical investigator, sponsor and clinical monitor should operate in strict accordance with the requirements of GCP throughout the study, and it should make sure to achieve standardized study procedure, accurate study data and reliable study conclusions. All valid medical records must pass final data review before they can be confirmed as valid.

# 12. Ethical Principles

This study strictly follows the Declaration of Helsinki and the principles of GCP in China.

# 13. References

[1] Li, A., X. Jiao, F. L. Munier, D. F. Schorderet, W. Yao, F. Iwata, M. Hayakawa, A. Kanai, M. Shy Chen, R. Alan Lewis, J. Heckenlively, R. G. Weleber, E. I. TrabouLsi, Q. Zhang, X. Xiao, M. Kaiser-Kupfer, Y. V. Sergeev and J. F. Hejtmancik (2004). "Bietti crystalline corneoretinal dystrophy is caused by mutations in the novel gene CYP4V2." Am J Hum Genet 74(5): 817-826.

[2] Mansour, A. M., S. H. Uwaydat and C. C. Chan (2007). "Long-term follow-up in Bietti crystalline dystrophy." Eur J Ophthalmol 17(4): 680-682.

[3] Lin, J., K. M. Nishiguchi, M. Nakamura, T. P. Dryja, E. L. Berson and Y. Miyake (2005). "Recessive mutations in the CYP4V2 gene in East Asian and Middle Eastern patients with Bietti crystalline corneoretinal dystrophy." J Med Genet 42(6): e38.

[4] Zanger, U. M. and M. Schwab (2013). "Cytochrome P450 enzymes in drug metabolism: reguLation of gene expression, enzyme activities, and impact of genetic variation." Pharmacol Ther 138(1): 103-141.

[5] Yanai, R., L. MuLki, E. Hasegawa, K. Takeuchi, H. Sweigard, J. Suzuki, P. Gaissert, D. G. Vavvas, K. H. Sonoda, M. Rothe, W. H. Schunck, J. W. Miller and K. M. Connor (2014). "Cytochrome P450-generated metabolites derived from omega-3 fatty acids attenuate neovascuLarization." Proc Natl Acad Sci U S A 111(26): 9603-9608.

[6] Lockhart, C. M., M. Nakano, A. E. Rettie and E. J. Kelly (2014). "Generation and characterization of a murine model of Bietti crystalline dystrophy." Invest Ophthalmol Vis Sci 55(9): 5572-5581.

[7] Nakano, M., E. J. Kelly and A. E. Rettie (2009). "Expression and characterization of CYP4V2 as a fatty acid omega-hydroxylase." Drug Metab Dispos 37(11): 2119-2122.

[8] Nakano, M., E. J. Kelly, C. Wiek, H. Hanenberg and A. E. Rettie (2012). "CYP4V2 in Bietti's crystalline dystrophy: ocuLar localization, metabolism of omega-3-polyunsaturated fatty acids, and functional deficit of the p.H331P variant." Mol Pharmacol 82(4): 679-686.

[9] Hata, M., H. O. Ikeda, S. Iwai, Y. Iida, N. Gotoh, I. Asaka, K. Ikeda, Y. Isobe, A. Hori, S. Nakagawa, S. Yamato, M. Arita, N. Yoshimura and A. Tsujikawa (2018). "Reduction of lipid accumuLation rescues Bietti's crystalline dystrophy phenotypes." Proc Natl Acad Sci U S A 115(15): 3936-3941.

[10] Simo, R., M. Villarroel, L. Corraliza, C. Hernandez and M. Garcia-Ramirez (2010). "The retinal pigment epithelium: something more than a constituent of the blood-retinal barrier--implications for the pathogenesis of diabetic retinopathy." J Biomed Biotechnol 2010: 190724.

[11] Hartong, D. T., E. L. Berson and T. P. Dryja (2006). "Retinitis pigmentosa." Lancet 368(9549): 1795-1809.

[12] Tian, R., S. R. Wang, J. Wang and Y. X. Chen (2015). "Novel CYP4V2 mutations associated with Bietti crystalline corneoretinal dystrophy in Chinese patients." Int J Ophthalmol 8(3): 465-469.

[13] Lee, K. Y., A. H. Koh, T. Aung, V. H. Yong, K. Yeung, C. L. Ang and E. N. Vithana (2005). "Characterization of Bietti crystalline dystrophy patients with CYP4V2 mutations." Invest Ophthalmol Vis Sci 46(10): 3812-3816.

[14] Xiao, X., G. Mai, S. Li, X. Guo and Q. Zhang (2011). "Identification of CYP4V2 mutation in 21 families and overview of mutation spectrum in Bietti crystalline corneoretinal dystrophy." Biochem Biophys Res Commun 409(2): 181-186.

[15] Jiao, X., A. Li, Z. B. Jin, X. Wang, A. Iannaccone, E. I. TrabouLsi, M. B. Gorin, F. Simonelli and J. F. Hejtmancik (2017). "Identification and popuLation history of CYP4V2 mutations in patients with Bietti crystalline corneoretinal dystrophy." Eur J Hum Genet 25(4): 461-471.

[16] Bainbridge, J. W., A. J. Smith, S. S. Barker, S. Robbie, R. Henderson, K. Balaggan, A. Viswanathan, G. E. Holder, A. Stockman, N. Tyler, S. Petersen-Jones, S. S. Bhattacharya, A. J. Thrasher, F. W. Fitzke, B. J. Carter, G. S. Rubin, A. T. Moore and R. R. Ali (2008). "Effect of gene therapy on visual function in Leber's congenital amaurosis." N Engl J Med 358(21): 2231-2239.

[17] Bainbridge, J. W., M. S. Mehat, V. Sundaram, S. J. Robbie, S. E. Barker, C. Ripamonti, A. Georgiadis, F. M. Mowat, S. G. Beattie, P. J. Gardner, K. L. Feathers, V. A. Luong, S. Yzer, K. Balaggan, A. Viswanathan, T. J. de Ravel, I. Casteels, G. E. Holder, N. Tyler, F. W. Fitzke, R. G. Weleber, M. Nardini, A. T. Moore, D. A. Thompson, S. M. Petersen-Jones, M. Michaelides, L. I. van den Born, A. Stockman, A. J. Smith, G. Rubin and R. R. Ali (2015). "Long-term effect of gene therapy on Leber's congenital amaurosis." N Engl J Med 372(20): 1887-1897.

[18] Jacobson, S. G., A. V. Cideciyan, A. J. Roman, A. Sumaroka, S. B. Schwartz, E. Heon and W. W. Hauswirth (2015). "Improvement and decline in vision with gene therapy in childhood blindness." N Engl J Med 372(20): 1920-1926.

[19] AyalasomayajuLa, S. P. and U. B. Kompella (2002). "Induction of vascuLar endothelial growth factor by 4-hydroxynonenal and its prevention by glutathione precursors in retinal pigment epithelial cells." Eur J Pharmacol 449(3): 213-220.

[20] Fleming, I. (2014). "The pharmacology of the cytochrome P450 epoxygenase/soluble epoxide hydrolase axis in the vascuLature and cardiovascuLar disease." Pharmacol Rev 66(4): 1106-1140.

[21] Gao, J., J. Z. Cui, E. To, S. Cao and J. A. Matsubara (2018). "Evidence for the activation of pyroptotic and apoptotic pathways in RPE cells associated with NLRP3 inflammasome in the rodent eye." J Neuroinflammation 15(1): 15.

[22] Lin, J., K. M. Nishiguchi, M. Nakamura, T. P. Dryja, E. L. Berson and Y. Miyake (2005). "Recessive mutations in the CYP4V2 gene in East Asian and Middle Eastern patients with Bietti crystalline corneoretinal dystrophy." J Med Genet 42(6): e38.
